# Healthcare Services and Burden of the Top Two Mental Disorders Among Women of Childbearing Age Across 204 Countries and Territories, 1990–2021

**DOI:** 10.1155/da/8872094

**Published:** 2026-04-01

**Authors:** Chen Chen, Ning Huang, Xinglin Feng, Bingqian Wang, Qi Jiang, Ban Hu, Mingyu Zhang, Zheng Liu, Jing Guo

**Affiliations:** ^1^ Department of Health Policy and Management, School of Public Health, Peking University, 38 Xueyuan Road, Beijing, 100191, China, pku.edu.cn; ^2^ Department of Maternal and Child Health, School of Public Health, Peking University, 38 Xueyuan Road, Beijing, 100191, China, pku.edu.cn

**Keywords:** anxiety disorders, depressive disorders, Global Burden of Disease Study, women of childbearing age

## Abstract

**Background:**

Mental health is a global health concern, particularly the burden among women of childbearing age (WCBA). We aimed to investigate the patterns and trends of the top two common mental disorders among WCBA from 1990 to 2021.

**Methods:**

Data were retrieved from the Global Burden of Disease Study 2021. The temporal trends were examined using joinpoint regression analysis. We utilized local regression smoothing models to fit the curves for correlations between age‐standardized rates (ASRs) and sociodemographic index (SDI) and Spearman correlation analysis to examine the correlations with health‐related indexes.

**Findings:**

The top two common mental disorders were depressive and anxiety disorders. The ASRs worldwide fluctuated and increased from 1990 to 2021. Annual percentage changes of ASRs of depressive disorders indicated increasing trends in high SDI regions, while those of anxiety disorders increased in regions with middle SDI, low–middle SDI, and low SDI. The 45–49 age group showed the highest global prevalence rate for depressive disorders in 2021. Joinpoint analysis revealed upward trends in depressive disorders’ ASRs, with the most notable increase during the 2019–2021 period, consistent with anxiety disorders. The associations between ASRs and SDI exhibited different patterns. The incidence of depressive and anxiety disorders showed significant correlations with various health‐related indexes.

**Conclusions:**

The global burdens of depressive and anxiety disorders among WCBA showed rising trends, and patterns differed in age and time period, underscoring the urgent need for targeted prevention and healthcare strategies to mitigate the burden among WCBA worldwide.

## 1. Introduction

Mental health in women of childbearing age (WCBA) has become a growing public health concern worldwide [1, 2]. According to the World Health Organization definition, the 15–49‐year age group was defined as WCBA [3]. Among the 12 main mental disorders, the most common mental disorders in WCBA were depressive and anxiety disorders, and the combination of depressive and anxiety disorders was the most common comorbidity [4, 5]. Compared to men, women are twice as likely to suffer from depressive and anxiety disorders [6–8]. Taking into account the intricate global socioeconomic development trends, including the phenomenon of declining birth rates and the intensified competitive landscape within the female labor market [9, 10], the burden of mental disorders is rising in WCBA [2]. However, the emotional stability and resilience in WCBA may be directly associated with the ability to provide a nurturing and supportive environment for the next generation, thereby influencing their cognitive, emotional, and social development [11]. Therefore, it is imperative to have a comprehensive understanding of the global mental health among WCBA to develop the targeted and efficacious implementation.

There are significant disparities across regions and countries in the burden of depressive and anxiety disorders in females. A systematic review found that mental disorders were more prevalent, particularly among women living in poverty in low‐ and middle‐income countries (such as Brazil, Chile, India, Zimbabwe, etc.) [12]. Notably, women living in low‐income circumstances in developed countries also have a high prevalence rate of depressive disorders [13], highlighting a correlation between late‐stage depressive disorder diagnosis and lower socioeconomic status. Insufficient healthcare services and inadequate medical resources may lead to delayed mental disorder screening and treatment, thereby increasing the incidence and mortality rates of these diseases. Female disadvantage persists in political, economic, social, and health‐related areas [14]. For example, South Asia has major gender inequities and high rates of gender‐based violence, with about 30% of women affected by perinatal depressive disorders [15, 16]. Similarly, prevalence rates of anxiety disorders may be as equally high or higher [17]. Notably, the burden of mental disorders among WCBA in different regions and countries needs comprehensive research for developing more targeted prevention and control strategies.

The Global Burden of Diseases, Injuries, and Risk Factors Study (GBD) offers a comprehensive epidemiological dataset to assess the burden of mental disorders in 21 regions and 204 countries and territories, providing a unique opportunity to understand the potential burden trends from 1990 to 2021. WCBA represents a crucial population group for reproductive health and family planning [18]. It is crucial to provide effective interventions for depressive and anxiety disorders during the reproductive age period, contributing to improving population fertility issues and women’s health globally. Therefore, this study focused on the top two major mental disorders in 2021. Using the global disease burden database, we aimed to estimate (1) the contribution of major mental disorders among WCBA in 2021, (2) the global, regional, and national burdens of the top two mental disorders, (3) the age patterns and temporal trends of their prevalence, incidence, and disability‐adjusted life‐years (DALYs) among WCBA, and (4) the associations between disease burdens and health‐related variables at the country‐level, to provide insights for policies and strategies to different regions or countries regarding prevention, screening, and treatment, ultimately benefiting women’s fertility health.

## 2. Methods

### 2.1. Study Data Acquisition and Download

In this research, we utilized data on two mental health disorders from the GBD 2021. The scope of these data have been elaborated in previously published material [18]. GBD 2021 provided estimates of the burden of nine major mental disorders. Based on the analysis of the components of mental disorders, we performed a detailed analysis on the epidemiological trends of mental disorders with the top two burdens. We extracted repeated cross‐sectional data, encompassing numbers and rates of the prevalence, incidence, and DALYs of mental disorders and their 95% uncertainty interval (UI) within the age range of 15–49 years from the GBD 2021 through the GBD Results Tool (https://vizhub.healthdata.org/gbd-results/).

### 2.2. The Sociodemographic Index (SDI)

The SDI, a composite index of sociodemographic development status, is calculated as the geometric mean of 0–1 indices on the following metrics: total fertility rate among individuals under 25 years old, the average education level for those aged 15 and above, and lag‐distributed income per capita. The 204 countries in GBD are categorized into five quintiles as low, low–middle, middle, high–middle, and high, based on their country‐level SDI estimates.

### 2.3. The Health‐Related Variables Among WCBA in 2021

We obtained the data of health‐related variables on human resources for nursing and midwifery (the proportion of male nursing personnel [19] and nursing and midwifery personnel (per 10,000) [20]), the environmental, social, and governance (ESG) index [21], the healthcare index [22], and the quality of life (QoL) index [23] online, which were multidimensional structural factors potentially associated with mental health among WCBA [24]. These variables were selected to reflect access to reproductive and maternal health services, healthcare system capacity, broader socioenvironmental contexts, and overall living conditions. Human resources for nursing and midwifery includes the proportion of male nursing personnel and nursing and midwifery personnel per 10,000 population. The ESG index is an efficient tool to measure ESG risk exposure, reflecting the environment, human rights, and health and safety in a specific country. The score for a country is displayed on a scale ranging from 0 to 100, with 0 representing the lowest risk and 100 indicating the highest risk. The healthcare index reflects a country’s overall level of healthcare services, encompassing factors such as the skill and competency of medical staff, speed in completing examinations and reports, and equipment for modern diagnosis and treatment. To estimate the overall well‐being and life satisfaction within a country, the QoL index applies an empirical formula that combines the purchasing power index, pollution index, house price to income ratio, cost of living index, safety index, health care index, traffic commute time index, and climate index.

### 2.4. Statistical Analysis

The age‐standardized rates (ASRs) were computed per 100,000 individuals of WCBA from 15 to 49 years, using the formula:

(1) ASR=∑i=1Aaiwi∑i=1Awi×100000.,

In the equation, *a*_*i*_ signifies the age‐specific rate in the *i*th age group, while *w*_*i*_ denotes the count of people in the corresponding *i*th age group as per the GBD 2021 standard population. *A* is the total number of age groups.

In this study, the joinpoint regression analysis model was conducted to assess temporal trends in disease prevalence, incidence, or DALYs. The core idea of this statistical model is to establish segmented regression according to the temporal characteristics of disease distribution, enabling a more detailed evaluation of the distinct characteristics of disease changes across various segments of the overall time period [25]. The model computes the annual percent change (APC), the average APC (AAPC) and the accompanying 95% confidence interval (CI) to delineate prevalence trends across delineated time frames and perform a holistic appraisal of the observed trends spanning the study period [26, 27]. An APC or AAPC estimate, accompanied by a 95% CI lower bound surpassing zero, indicates an upward trend within the specified interval. Conversely, if the 95% CI upper bound of an APC or AAPC estimate is below zero, it signifies a downward trend in the specific period. It suggests a stable trend if the 95% CI for the APC or AAPC includes zero.

We employed local regression smoothing models to fit the correlation between the burdens of mental disorders and SDI across 21 regions among WCBA. Additionally, Spearman correlation analysis was used to compute the *r* indices and *p* values for the relationships between age‐standardized incidence rates (ASIRs) and health‐related variables among WCBA in 2021. *p* < 0.05 is regarded as statistically significant. All statistical analyses were conducted using R software (Version 4.3.2).

### 2.5. Ethics Statement

The Institutional Review Board of the University of Washington has reviewed and approved a waiver of informed consent online (https://www.healthdata.org/research-analysis/gbd).

## 3. Results

### 3.1. The Contribution of Major Mental Disorders Among WCBA

Figure S1 presents the proportions of mental disorders in WCBA caused by specific etiologies at the global and regional levels from the years 1990 to 2021. Overall, the most commonly occurring mental disorders were depressive (23.8%–50.8%) and anxiety disorders (26.4%–53.8%) in each region. The proportion of anxiety disorders prevalent cases (37.1%) was more than that of depressive disorders (34.4%) in 2021 globally. In low SDI and low–middle SDI regions, the proportion of prevalent cases of depressive disorders exceeded anxiety disorders.

### 3.2. Global, Regional, and National Burden of Depressive and Anxiety Disorders Among WCBA

#### 3.2.1. Global Trends

The global burden of depressive and anxiety disorders among WCBA worldwide fluctuated and rose from 1990 to 2021. Overall, there were 77,722,841 estimated incident cases of depressive disorders in WCBA (95% UI 93,343,266–65,414,928) in 1990 and 133,248,593 prevalent cases (95% UI 162,189,361–108,943,181) in 2021. Additionally, anxiety disorders accounted for 10,728,548 estimated incident cases (95% UI 14,217,155–8,142,949) in 1990 and 18,962,131 cases (95% UI 24,805,469–14,505,459) in 2021.

In 2021, the overall ASIR of depressive disorders was 6837.27 per 100,000 population (95% UI 5590.11–8322.29), with an age‐standardized prevalence rate (ASPR) of 6220.98 per 100,000 population (95% UI 5371.96–7308.26) (Table 1). The overall age‐standardized DALYs rate (ASDR) of depressive disorders was 1079.73 per 100,000 population (95% UI 722.08–1491.21) (Table 1). By contrast, higher ASPR was found in anxiety disorders (7097.14, 95% UI 5780.74–8756.62), and lower ASIR (972.99, 95% UI 744.31–1272.82) and ASDR (844, 95% UI 565.54–1170.47) were recorded (Table 1). The ASPR, ASIR, and ASDR of anxiety disorders increased by 0.18%, 0.21%, and 0.17% from 1990 to 2021, respectively (Table 1).

**Table 1:** Age‐standardized prevalence, incidence, and disability‐adjusted life years rates of depressive and anxiety disorders in 2021 and annual percentage change from 1990 to 2021 among women of childbearing age.

Regions	Depressive disorders (95% uncertainty interval)						Anxiety disorders					
	ASPR per 100,000 in 2021	ASIR per 100,000 in 2021	ASDR per 100,000 in 2021	Annual percentage change in ASPR, 1990–2021	Annual percentage change in ASIR, 1990–2021	Annual percentage change in ASDR, 1990–2021	ASPR per 100,000 in 2021	ASIR per 100,000 in 2021	ASDR per 100,000 in 2021	Annual percentage change in ASPR, 1990–2021	Annual percentage change in ASIR, 1990–2021	Annual percentage change in ASDR, 1990–2021
Overall (female)	6220.98 (5371.96, 7308.26)	6837.27 (5590.11, 8322.29)	1079.73 (722.08, 1491.21)	−0.02% (−0.17, 0.13)	−0.11% (−0.32, 0.1)	−0.06% (−0.24, 0.12)	7097.14 (5780.74, 8756.62)	972.99 (744.31, 1272.82)	844 (565.54, 1170.47)	0.18% (0.02, 0.33)	0.21% (0.06, 0.36)	0.17% (0.02, 0.32)
High SDl	7629.55 (6650.03, 8819.17)	8908.82 (7550.29, 10781.01)	1377.95 (956.5, 1895.89)	0.33% (0.16, 0.49)	0.49% (0.27, 0.71)	0.4% (0.21, 0.59)	9546.35 (7856.62, 11802.74)	1173.34 (892.38, 1539.72)	1128.97 (750.42, 1585.82)	0.12% (−0.1, 0.34)	0.21% (0.03, 0.39)	0.1% (−0.11, 0.32)
High–middle SDl	5694.63 (4928.95, 6722.86)	5852.38 (4794.51, 7168.22)	963.27 (646.17, 1335.82)	−0.06% (−0.22, 0.1)	−0.24% (−0.47, −0.01)	−0.15% (−0.34, 0.05)	6808.23 (5499.25, 8438.74)	897.32 (671.27, 1182.5)	815.12 (537.46, 1138.8)	0% (−0.16, 0.17)	0% (−0.16, 0.16)	0% (−0.16, 0.16)
Middle SDl	5537.56 (4819.56, 6439.61)	5834.02 (4797.94, 7082.59)	941.03 (626.82, 1284.75)	0.03% (−0.13, 0.18)	−0.12% (−0.35, 0.11)	−0.05% (−0.24, 0.14)	7345.81 (6043.27, 8927.01)	1003.16 (771.39, 1308.09)	875.98 (594.12, 1213.9)	0.37% (0.2, 0.53)	0.31% (0.15, 0.48)	0.35% (0.19, 0.52)
Low–middle SDl	6530.56 (5578.11, 7818)	7456.08 (6015.56, 9276.55)	1149.02 (766.03, 1592.11)	−0.29% (−0.48, −0.1)	−0.5% (−0.75, −0.24)	−0.37% (−0.59, −0.15)	6491.66 (5308.28, 8043.48)	951.03 (729.36, 1251.87)	769.56 (521.17, 1080.95)	0.47% (0.33, 0.62)	0.41% (0.25, 0.56)	0.48% (0.34, 0.63)
Low SDI	6529.33 (5523.71, 7920.21)	7218.52 (5840.87, 9072.01)	1130.08 (748.96, 1576.53)	−0.22% (−0.34, −0.1)	−0.33% (−0.5, −0.16)	−0.24% (−0.38, −0.1)	5800.54 (4578.89, 7371.19)	851.63 (641.36, 1129.34)	688.35 (445.16, 997.03)	0.2% (0.07, 0.32)	0.18% (0.06, 0.31)	0.23% (0.11, 0.35)
Andean Latin America	5381.39 (4343.22, 6721.89)	6333.45 (4875.19, 8186.22)	970.01 (610.5, 1379.98)	0.18% (−0.11, 0.47)	0.14% (−0.25, 0.53)	0.16% (−0.18, 0.5)	12159.24 (8686.41, 16799.21)	1544.28 (1072.2, 2252.43)	1453.43 (893.81, 2130.89)	0.48% (0.19, 0.78)	0.41% (0.11, 0.71)	0.49% (0.19, 0.78)
Australasia	8370.39 (6744.14, 10337.36)	10017.34 (7654.79, 13028.17)	1536.23 (1018.03, 2201.02)	0.17% (0.03, 0.3)	0.19% (0.03, 0.35)	0.19% (0.04, 0.33)	11308 (8179.07, 15029.56)	1261.84 (892.23, 1775.26)	1332.77 (840.92, 1984.27)	0.23% (0.14, 0.32)	0.17% (0.04, 0.3)	0.23% (0.14, 0.32)
The Caribbean	6541.12 (5292.75, 8343.63)	8060.5 (6209.91, 10582.1)	1201.97 (776.64, 1713.29)	−0.33% (−0.54, −0.13)	−0.44% (−0.7, −0.18)	−0.41% (−0.64, −0.18)	8645.95 (6511.67, 11713.94)	1166.66 (850.98, 1607.97)	1024.39 (650.15, 1488.87)	0.27% (0.12, 0.42)	0.22% (0.07, 0.37)	0.26% (0.11, 0.41)
Central Asia	5290.44 (4324.48, 6473.68)	5635.93 (4469.96, 7110.86)	910.07 (596.5, 1303.44)	0.23% (0.09, 0.36)	0.22% (0.03, 0.41)	0.23% (0.07, 0.39)	3975.14 (2990.44, 5252.02)	610.91 (444.39, 819.81)	476.01 (301.87, 694.22)	0.18% (0.01, 0.35)	0.16% (−0.01, 0.33)	0.18% (0.01, 0.35)
Central Europe	4918.48 (4152.19, 5860.12)	4944.3 (3941.48, 6166.23)	819.58 (540.88, 1138.53)	−0.15% (−0.35, 0.06)	−0.38% (−0.7, −0.06)	−0.25% (−0.51, 0.01)	6869.13 (5445.2, 8830.16)	982.33 (738.29, 1289.65)	821.67 (539.51, 1178.95)	0.29% (0.08, 0.5)	0.28% (0.07, 0.5)	0.3% (0.09, 0.51)
Central Latin America	6326.59 (5352.16, 7575.82)	7912.6 (6379.21, 9727.81)	1173.59 (764.69, 1628.9)	0.97% (0.8, 1.15)	1.16% (0.94, 1.38)	1.07% (0.87, 1.26)	8099.51 (6395.58, 10178.11)	1138.27 (847.46, 1527.04)	966.26 (633.59, 1409.19)	0.74% (0.49, 0.99)	0.71% (0.5, 0.92)	0.73% (0.48, 0.97)
Central Sub‐Saharan Africa	9031.63 (7215.44, 11573.59)	10977 (8357.78, 14645.92)	1640.54 (1056.02, 2327.58)	−0.01% (−0.1, 0.08)	−0.02% (−0.14, 0.1)	0.02% (−0.08, 0.13)	5858.25 (4270.68, 8084.96)	837.36 (584.15, 1173.82)	691.86 (433.97, 1062.91)	0.13% (0.02, 0.24)	0.13% (0.02, 0.25)	0.17% (0.06, 0.28)
East Asia	4070.97 (3545.24, 4745.6)	3141.52 (2610.33, 3773.86)	607.38 (414.73, 824.47)	−0.51% (−0.67, −0.35)	−1.14% (−1.41, −0.88)	−0.8% (−1.01, −0.6)	4965.08 (4065.43, 6017.17)	698.97 (537.14, 910.94)	602.17 (412.61, 836.97)	−0.64% (−0.81, −0.48)	−0.52% (−0.67, −0.36)	−0.64% (−0.8, −0.48)
Eastern Europe	6464.51 (5471.4, 7561.7)	7247.24 (5802.43, 8989.45)	1132.7(750.71, 1583.79)	0.07% (−0.14, 0.27)	−0.02% (−0.3, 0.25)	0.04% (−0.2, 0.28)	6732.69 (5539.68, 8110.92)	959.68 (733.43, 1232.23)	801.23 (541.09, 1132.71)	0.28% (0.06, 0.5)	0.25% (0.03, 0.47)	0.28% (0.07, 0.5)
Eastern Sub‐Saharan Africa	7079.99 (5959.38, 8533.45)	7644.99 (6167.8, 9672.17)	1216.24 (796.74, 1697.91)	−0.17% (−0.28, −0.06)	−0.24% (−0.41, −0.08)	−0.17% (−0.31, −0.04)	6647.14 (5104.09, 8458.1)	986.97 (734.04, 1299.71)	791.43 (511.43, 1157.39)	0.09% (−0.05, 0.23)	0.08% (−0.06, 0.22)	0.13% (−0.01, 0.27)
High‐income Asia Pacific	4220.09 (3652.65, 4924.04)	4818.33 (4065.52, 5729.38)	762.49 (516.06, 1055.81)	0.22% (0.05, 0.4)	0.36% (0.16, 0.56)	0.3% (0.11, 0.49)	5286.94 (4167.47, 6608.63)	750.12 (562.62, 985.09)	636.09 (418.44, 890.69)	−0.17% (−0.33, −0.01)	−0.23% (−0.4, −0.07)	−0.17% (−0.33, −0.01)
High‐income North America	10350.16 (9070.74, 11785.55)	12472.28 (10616.52, 14803.62)	1900.89 (1345.95, 2573.86)	0.45% (0.22, 0.67)	0.8% (0.46, 1.15)	0.61% (0.34, 0.89)	12143.89 (9985.35, 14628.95)	1543.35 (1164.6, 1997.68)	1423.93 (980.24, 2004.08)	0.13% (−0.23, 0.49)	0.28% (0.03, 0.54)	0.1% (−0.25, 0.46)
North Africa and Middle East	8344.27 (6906.15, 10236.44)	10054.39 (7900.96,12848.8)	1518.75 (986.69, 2163.34)	0.31% (0.18, 0.45)	0.3% (0.13, 0.46)	0.3% (0.16, 0.45)	9985.72 (7695.7, 12949.81)	1142.19 (840.68, 1568.69)	1181.6 (750.73, 1693.89)	0.3% (0.17, 0.43)	0.31% (0.17, 0.46)	0.28% (0.15, 0.41)
Oceania	4438.45 (3576.58, 5542.11)	4139.42 (3130.04,5514.26)	717.16 (444.54, 1027.31)	−0.01% (−0.05, 0.03)	−0.15% (−0.22, −0.08)	−0.07% (−0.12, −0.01)	6242.65 (4457.83, 8622.02)	863.18 (602.31, 1239.53)	748.82 (454.43, 1146.74)	0.1% (0.02, 0.18)	0.11% (0.03, 0.19)	0.11% (0.03, 0.18)
South Asia	6313.76 (5378.48, 7440.85)	7199.62 (5921.01,8834.23)	1105.56 (738.45, 1523.45)	−0.57% (−0.8, −0.34)	−0.87% (−1.19, −0.55)	−0.69% (−0.97, −0.42)	5606.74 (4586.66, 6745.54)	902.58 (701.89, 1153.21)	662.42 (457.11, 909.75)	0.49% (0.29, 0.7)	0.45% (0.27, 0.64)	0.51% (0.31, 0.72)
South‐East Asia	4344.39 (3724.45, 5073.59)	3830.61 (3146.63,4729.35)	688.09 (462.97, 957.68)	0.2% (0.09, 0.3)	0.01% (−0.2, 0.22)	0.13% (−0.03, 0.28)	6735.72 (5454.33, 8291.61)	1009.32 (779.5, 1293.88)	810.83 (540.35, 1147.14)	0.32% (0.15, 0.49)	0.26% (0.09, 0.44)	0.32% (0.16, 0.49)
Southern Latin America	6473.18 (5282.94, 7974.35)	8039.93 (6441.34,10181.38)	1210.42 (804.24, 1696.65)	−0.23% (−0.46, −0.01)	−0.21% (−0.46, 0.04)	−0.22% (−0.45, 0.02)	11628.33 (8489.44, 15579.73)	1310 (918.91, 1847.28)	1380.12 (871.04, 1972.78)	0.04% (−0.17, 0.25)	0.11% (−0.06, 0.29)	0.04% (−0.17, 0.24)
Southern Sub‐Saharan Africa	7656.95 (6568.26, 8965.9)	8703.44 (7175.05,10705.81)	1327.02 (922.92, 1817.69)	0.44% (0.23, 0.64)	0.51% (0.23, 0.8)	0.44% (0.19, 0.69)	7140.76 (5709.16, 8785.09)	1018.93 (776.9, 1344.65)	835.03 (568.35, 1170.15)	0.3% (0.1, 0.5)	0.26% (0.06, 0.47)	0.26% (0.06, 0.46)
Tropical Latin America	7798.27 (6655.7, 9122.37)	9905.88 (8160.98,12041.45)	1446.23 (962.22, 1978.83)	−0.22% (−0.54, 0.1)	−0.34% (−0.72, 0.04)	−0.28% (−0.63, 0.06)	16057.67 (13026.4, 19310.09)	1833.87 (1399.12, 2404.22)	1891.15 (1288.02, 2631.97)	1.47% (0.95, 2)	1.06% (0.74, 1.39)	1.47% (0.96, 1.99)
Western Europe	8325.44 (7095.16, 10015.26)	10105.09 (8322.02,12694.88)	1533.8 (1037.16, 2135.97)	0.19% (0.03, 0.35)	0.23% (0.02, 0.43)	0.2% (0.02, 0.39)	10939.02 (8640.46, 13902.45)	1197.36 (891.24, 1594.86)	1298.07 (857.11, 1829.73)	0.23% (0.07, 0.39)	0.28% (0.13, 0.42)	0.22% (0.07, 0.38)
Western Sub‐Saharan Africa	5619.07 (4766.78, 6809)	5811.74 (4686.62,7362.16)	942.73 (631.27, 1320.02)	−0.16% (−0.23, −0.08)	−0.27% (−0.38, −0.15)	−0.18% (−0.27, −0.08)	4568.04 (3643.66, 5773.89)	633.42 (471.14, 843.14)	544.62 (356.37, 766.38)	0.15% (0.05, 0.25)	0.17% (0.02, 0.32)	0.18% (0.08, 0.28)

*Note:* ASDR, age‐standardized disability‐adjusted life years rates.

Abbreviations: ASIR, age‐standardized incidence rates; ASPR, age‐standardized prevalence rates.

#### 3.2.2. Regional Trends

In 2021, studies conducted at regional levels found that the ASRs of both depressive and anxiety disorders were highest in regions with a high SDI (Table 1). Geographically, the ASPR, ASIR, and ASDR of depressive disorders were highest in high‐income North America, at 10350.16 per 100,000 persons (95% UI 9070.74–11785.55), 12472.28 per 100,000 persons (95% UI 10616.52–14803.62), and 1900.89 per 100,000 persons (95% UI 1345.95–2573.86), followed by Central Sub‐Saharan Africa (Table 1; Figure 1A). Tropical Latin America demonstrated higher ASPR, ASIR, and ASDR of anxiety disorders, at 16057.67 per 100,000 persons (95% UI 13026.4–19310.09), 1833.87 per 100,000 persons (95% UI 1399.12–2404.22), and 1891.15 per 100,000 persons (95% UI 1288.02–2631.97), followed by Andean Latin America (Table 1; Figure 1A).

Figure 1Burden of depressive and anxiety disorders in women of childbearing age, from 1990 to 2021. (A) Maps showing (A1) age‐standardized prevalence rate, (A2) age‐standardized incidence rate, and (A3) age‐standardized DALYs rate of depressive and anxiety disorders in 204 countries and territories between 1990 and 2021. (B) Global temporal trends in age‐group‐specific age‐standardized prevalence rate, age‐standardized incidence rate, and age‐standardized DALYs rate for depressive (B1–B3) and anxiety (B4–B6) disorders, 1990–2021. DALYs, disability‐adjusted life‐years.

**Figure:**
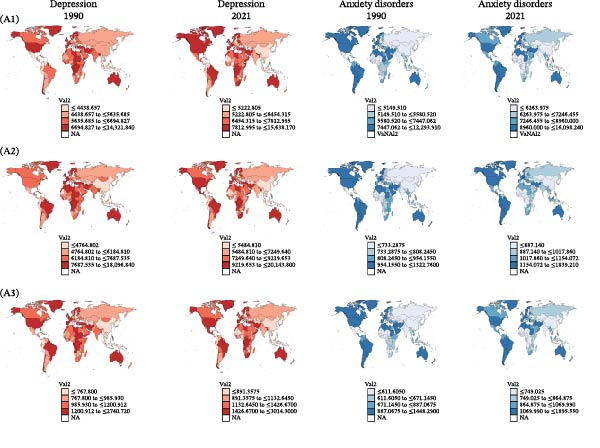
(A)

**Figure:**
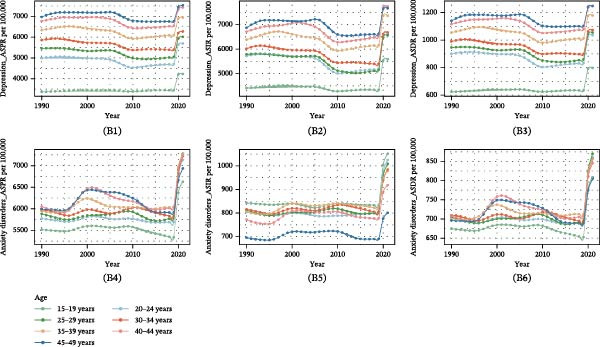
(B)

During the period from 1990 to 2021, the trend of changes in the ASRs of depressive and anxiety disorders varied across different world regions. For depressive disorders, increasing trends were observed in regions with a high SDI, at 0.33% for ASPR, 0.49% for ASIR, and 0.40% for ASDR (Table 1), while the other four SDI regions showed decreasing trends. Geographically, Central Latin America showed the most increasing change rate at 0.97% for ASPR, 1.16% for ASIR, and 1.07% for ASDR (Table 1; Figure 1A). By contrast, regions with a middle SDI, low–middle SDI, and low SDI showed an increasing change trend in ASRs of anxiety disorders (Table 1). Tropical Latin America also exhibited the most significant upward trend, at 1.47% for ASPR, 1.06% for ASIR, and 1.47% for ASDR (Table 1; Figure 1A).

#### 3.2.3. National Trends

In 2021, Greenland, Greece, and Lesotho recorded the highest ASPR of depressive disorders among all countries, with Greenland leading at 15638.17 per 100,000 population (95% UI 12258.72–19916.72), followed by Greece with 10867.51 per 100,000 population (95% UI 8014.92–14608.04) and Lesotho with 10798.47 per 100,000 population (95% UI 8396.93–13983.26), as shown in Table S1 and Figure 1A. Qatar experienced the most significant increase in prevalence cases, surging by 634.34% from 6060.4125 cases in 1990 to 44503.963 in 2021 (Table S2; Figure S2a). Similarly, in 2021, Greenland, Greece, and Lesotho also had the highest ASIR and ASDR of depressive disorders (Table S1). Moreover, Qatar showed the most increase in incidence cases and DALYs (Table S2; Figure S2b, c). The DALYs of depressive disorders in Cuba dropped from 46862.96 in 1990 to 30798.07 in 2021 (Table S2; Figure S2c).

In terms of anxiety disorders, Brazil reported the highest ASPR with 16098.24 cases per 100 000 individuals (95% UI 13070.46–19305.15), closely followed by Portugal (15047.65 per 100 000, 95% UI 10401.09–20397.11) and Paraguay (14802.02 per 100 000, 95% UI 10367.85–20058.43) in 2021 (Table S3; Figure 2B). Qatar witnessed the most significant increase in incident cases, a 636.64% rise from 749.75 in 1990 to 5523.00 in 2021 (Table S4; Figure S2b). In contrast, the United States Virgin Islands and Georgia saw a notable reduction in incidence cases of more than 20% from 1990 to 2021 (Table S4). Over a 32‐year period, Qatar and the United Arab Emirates both experienced DALY growth rates of more than 400% (Table S4; Figure S2c).

Figure 2Joinpoint regression analysis of global depressive and anxiety disorders burdens temporal trends, 1990–2021. (A) Age‐standardized prevalence rates for depressive disorders; (B) age‐standardized incidence rates for depressive disorders; (C) age‐standardized DALYs rates for depressive disorders; (D) age‐standardized prevalence rates for anxiety disorders; (E) age‐standardized incidence rates for anxiety disorders; (F) age‐standardized DALYs rates for anxiety disorders. APC, annual percentage change; DALYs, disability‐adjusted life‐years. An asterisk (*) indicates that the APC is significantly different from zero (*p* < 0.05). Points represent observed values, and lines represent fitted trends from the Joinpoint regression model.

**Figure:**
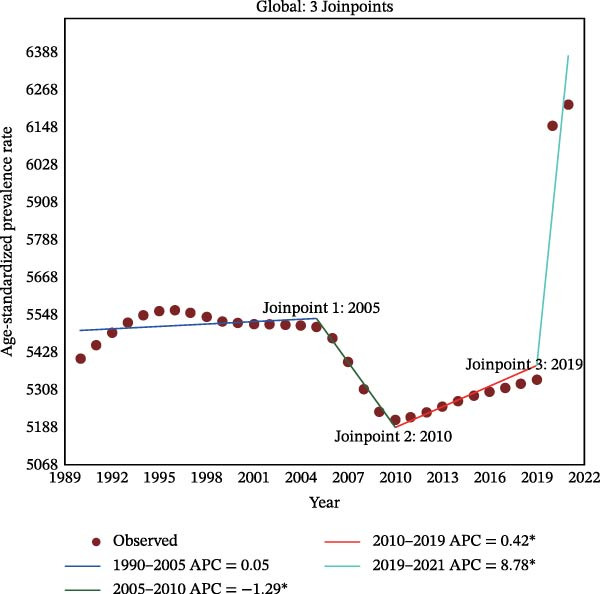
(A)

**Figure:**
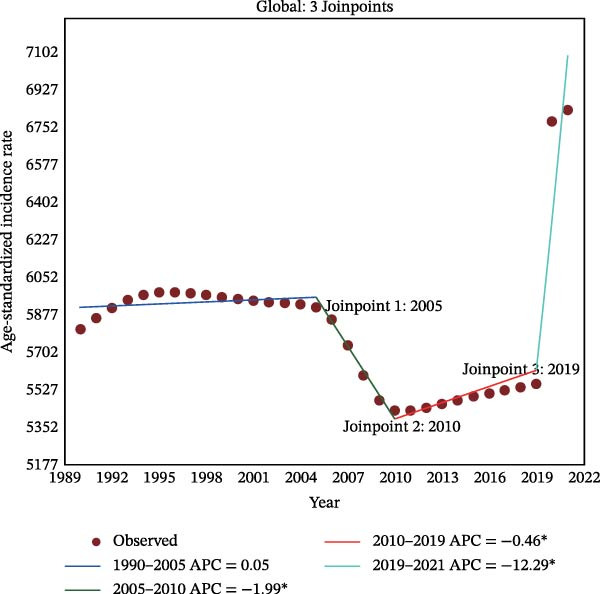
(B)

**Figure:**
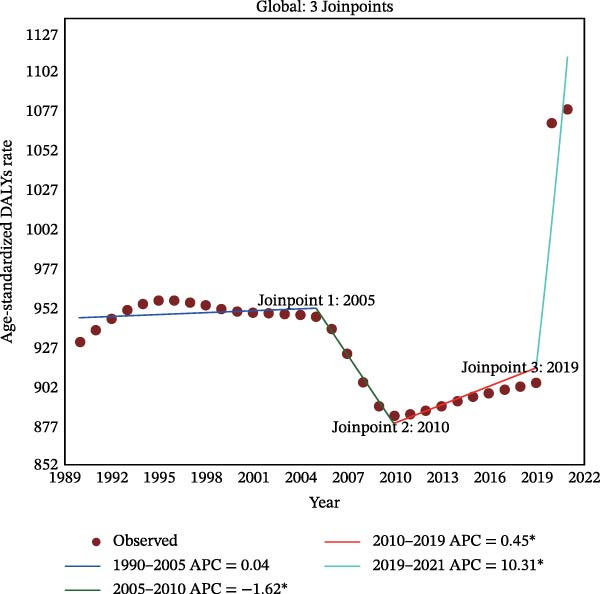
(C)

**Figure:**
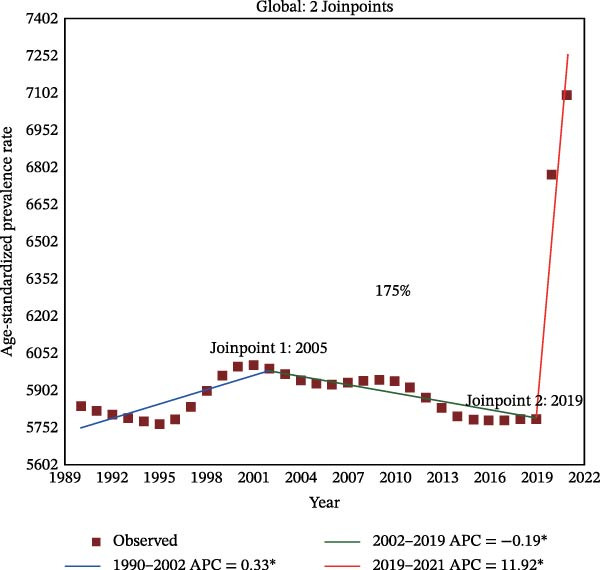
(D)

**Figure:**
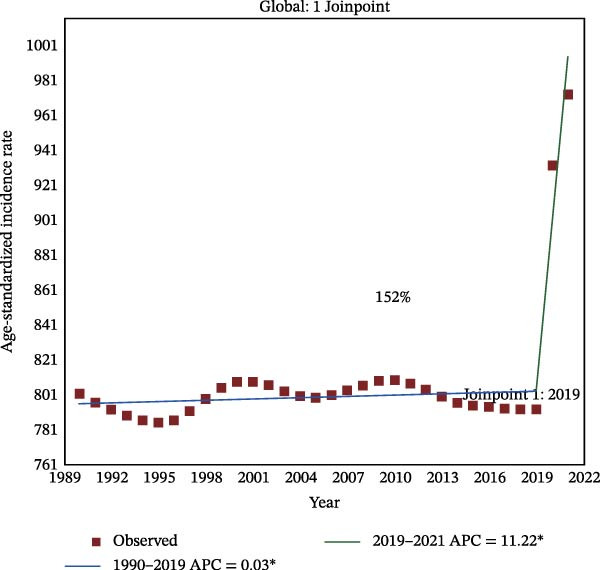
(E)

**Figure:**
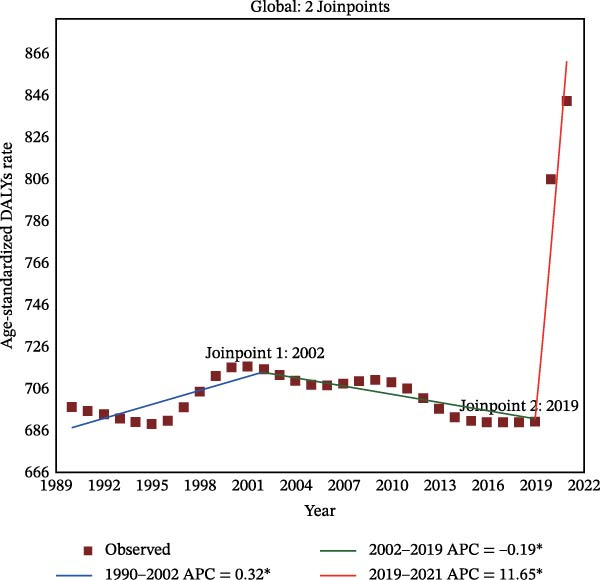
(F)

#### 3.2.4. Age Patterns and Overall Temporal Trends in Age Structures

For depressive disorders, the highest global ASPR was observed among WCBA aged 45–49 years, increasing with age in 2021 (Table S5; Figure 1B‐B1). Specifically, the ASIR and ASDR were highest in the 40–44‐year age group in 2021 (Table S5; Figure 1B‐B1,B2). The ASPR, ASIR, and ASDR of depressive disorders for all age groups initially experienced minor fluctuations from 1990 to 2005, then performed a decreasing trend from 2005 to 2010, and ultimately followed an increasing trend from 2019 to 2021 (Figure 1B‐B1–B3). From 2012 to 2018, the ASIRs in the 20–24‐year age group were higher than the ASIRs in the 25–29‐year age group (Figure 1B‐B2).

In 2021, the ASPR and ASDR of anxiety disorders were highest in the 25–29 age group (Table S6). Notably, the 15–19 age group showed the highest ASIR decreasing with age in 2021 (Table S6). From 1990 to 2021, the ASPRs and ASDRs for the 35–39, 40−44, and 45–49 age groups initially showed an upward trend, followed by a decline, but ultimately exhibited a significant increasing trend (Figure 1B‐B4, B6). Conversely, the ASIRs in all age groups demonstrated minor fluctuations from 1990 to 2019 (Figure 1B‐B5).

### 3.3. Temporal Joinpoint Analysis

Joinpoint regression analysis revealed that the ASPR, ASIR, and ASDR of depressive disorders exhibited global upward trends (AAPC_ASPR_ = 0.479%, 95% CI 0.329%–0.629%; AAPC_ASIR_ = 0.584%, 95% CI 0.422%–0.746%; AAPC_ASDR_ = 0.524%, 95% CI 0.376%–0.673%), with the most notable increase during the 2019–2021 period (APC_ASPR_ = 8.782%, 95% CI 6.756%–10.848%; APC_ASIR_ = 12.290%, 95% CI 10.035%–14.590%; APC_ASDR_ = 10.308%, 95% CI 8.380%–12.271%) (Figure 2A–C and Tables S7–S9). The joinpoint regression analysis of anxiety disorders ASRs showed a trend similar to that of depressive disorders, with an upward global trend (AAPC_ASPR_ = 0.752%, 95% CI 0.597%–0.907%; AAPC_ASIR_ = 0.719%, 95% CI 0.553%–0.884%; AAPC_ASDR_ = 0.734%, 95% CI 0.584%–0.884%), and the fastest increase occurring between 2019 and 2021 (APC_ASPR_ = 11.916%, 95% CI 9.393%–14.498%; APC_ASIR_ = 11.217%, 95% CI 8.365%–14.145%; APC_ASDR_ = 11.653%, 95% CI 9.232%–14.128%) (Figure 2D–F and Tables S10–S12). However, in contrast to other temporal trends, only a minor increasing trend was observed in the ASIR of anxiety disorders from 1990 to 2021 (APC = 0.032%, 95% CI −0.009%–0.074%) (Figure 2E and Table S11).

The top region with the highest burden of depressive disorders in 2021, high‐income North America, showed a periodic downward trend in ASPR from 1997 to 2019 (APC_ASPR_ = −0.175%, 95% CI −0.222% to −0.128%;), and in ASIR and ASDR from 1999 to 2019 (APC_ASIR_ = −0.407%, 95% CI −0.582% to −0.231%; APC_ASDR_ = −0.322%, 95% CI −0.442% to −0.202%) (Tables S7–S9). For anxiety disorders, Tropical Latin America, with the highest disease burden in 2021, showed a periodic downward trend in burden from 2010 to 2015 (APC_ASPR_ = −4.347%, 95% CI −4.928% to −3.763%; APC_ASIR_ = −2.955%, 95% CI −3.413% to −2.496%; APC_ASDR_ = −4.315%, 95% CI −4.865% to −3.762%), and a minor or upward trend in other time periods (Tables S10–S12).

### 3.4. The Associations Between SDI and ASRs

**Figure 3:**
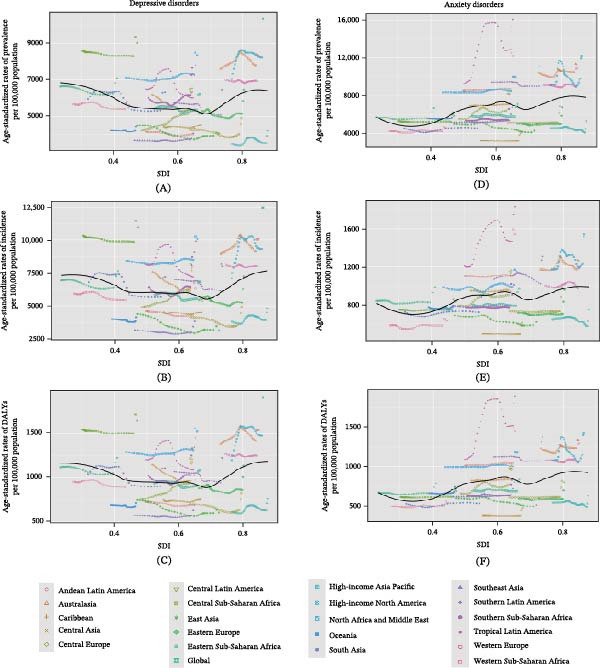
Age‐standardized rates of prevalence, incidence, and DALYs of depression and anxiety disorders globally and for 21 GBD regions, by SDI, from 1990 to 2021. Age‐standardized prevalence rates of depression (A), anxiety disorders (D), by SDI. Age‐standardized incidence rates of depression (B), anxiety disorders (E), by SDI. Age‐standardized DALYs rates of depression (C), anxiety disorders (F), by SDI. Expected values with 95% CI, based on SDI and disease rates in all regions, are shown as a solid line and shaded area; 32 points are plotted for each region and show the observed age‐standardized prevalence, incidence, or DALYs rates for each year from 1990 to 2021. DALYs, disability‐adjusted life‐years; GBD, Global Burden of Diseases, Injuries, and Risk Factors Study; SDI, sociodemographic index.

From 1990 to 2021, across 21 regions, the overall ASPR, ASIR, and ASDR of depressive disorders initially declined, then slightly increased at SDI of 0.45 and declined at SDI of 0.85 with rising SDI, approximately (Figure 3A–C). In ASRs of depressive disorders, Central Asia, Central Latin America, and Southern Latin America closely followed expected trends over the study period from 1990 to 2021. Overall, ASRs of depressive disorders exhibited similar patterns resembling “W”‐shaped curves.

Meanwhile, the overall ASRs of anxiety disorders initially increased with rising SDI, then declined around an SDI of 0.6 and increased around an SDI of 0.7 (Figure 3D–F). The Caribbean, Western Europe, Southern Latin America, Central Latin America, and East Asia closely followed expected trends in ASRs of anxiety disorders. Notably, Tropical Latin America remained well above expected levels for anxiety disorders throughout the study period, with large fluctuations in ASRs. Overall, a similar pattern resembling an “M”‐shaped curve was found in ASRs of anxiety disorders.

### 3.5. The Association Between Health‐Related Variables Among WCBA and ASIRs in 2021

Tables S13, S14 show the health‐related variables among WCBA across the top 20 and bottom 20 countries and territories in 2021 (sorted by 2021 ASIR of depressive and anxiety disorders). The United States of America had a higher nursing and midwifery personnel rate among the top 20 countries and territories, when no matter countries were sorted by the 2021 ASIR of depressive or anxiety disorders (131.5 per 10,000, Table S13). Sweden had a higher ESG Index (171.4), while Taiwan (Province of China) exhibited a higher healthcare index (86.4, Table S13). When countries were sorted by 2021 ASIR of anxiety disorders, New Zealand had the highest QoL Index (175.8) in the top 20 countries and territories, and Chad had the most proportion of male nursing personnel (64.9%) in the bottom 20 countries and territories (Table S14).

**Figure 4:**
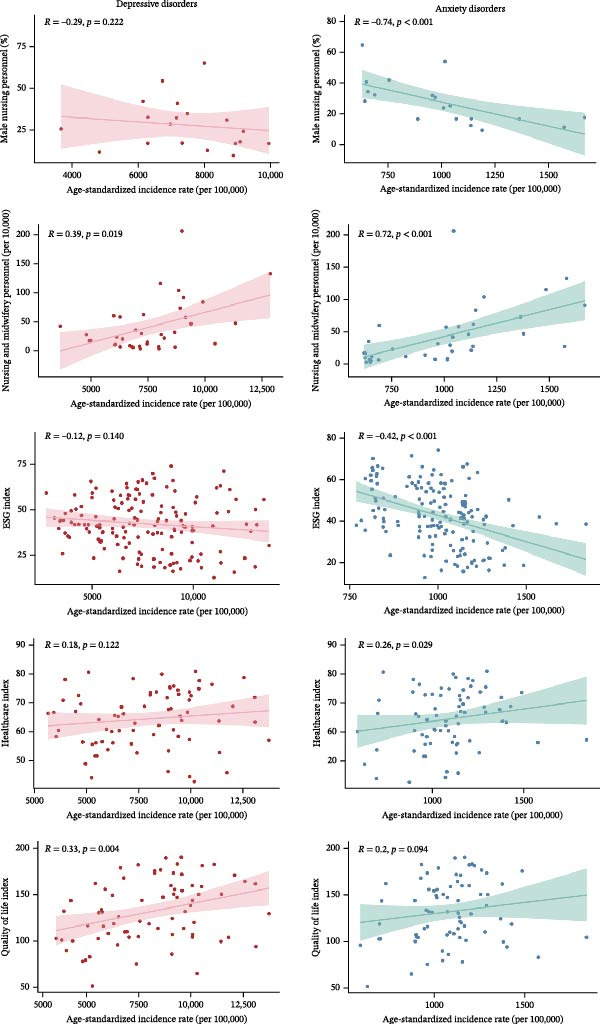
Associations of age‐standardized incidence rates for depressive and anxiety disorders with health‐related variables among women of childbearing age, in 2021.

Figure 4 and Table S15 show the associations between ASIRs and health‐related variables among WCBA in 2021. The nursing and midwifery personnel rate (*r*_*s*_ = 0.385, *p* = 0.019) and QoL Index (*r*_*s*_ = 0.330, *p* = 0.004) were positively related to ASIR of depressive disorders. In terms of anxiety disorders, the ASIR was significantly correlated with the proportion of male nursing personnel, nursing and midwifery personnel rate (per 10,000), the ESG index, and the healthcare index, and the Spearman correlation coefficients were −0.741, 0.723, 0.418, and 0.256, respectively. However, there was no significant association between the ASIR of anxiety disorders and the QoL Index.

## 4. Discussion

Depressive and anxiety disorders remain a significant public health challenge worldwide, with their impact on prevalence, incidence, and DALYs drawing substantial research and society attention. Our study offers a comprehensive estimation and detailed portrayal of the burdens and temporal trends of the two major mental disorders during 32 years, covering a wide geographic scope that includes six continents, 204 countries, and seven age groups among WCBA. In particular, we introduce an innovative assessment of temporal trends in the burden of depressive and anxiety disorders and health‐related variables and indexes associated with increasing burdens, identifying critical junctures of significant shifts in varied metrics that have occurred for the first time.

The global rises in the ASRs of mental disorders among WCBA from 1990 to 2021are mainly attributable to the increases in depressive and anxiety disorders. This finding aligns with previous evidence [8, 28, 29], suggesting the urgent need for heightened attention to depressive and anxiety disorders burden during women’s reproductive years. The individual and global economic and social situation may have an essential effect on women’s mental health in anxiety specifically. The southern countries of Europe, where the unemployment rates are pretty high, have a higher prevalence of anxiety disorders than men (18.8% women vs. 9.4% men) [30]. Other studies also reported the important role of social roles in the result of mental disorders under struggling economical situations [31, 32]. In addition, our findings revealed that rapid rises in the burden of depressive and anxiety disorders among WCBA from 2019 to 2021 globally may potentially reflect the mental health consequences of the COVID‐19 pandemic. According to the social sphere, a growing body of evidence has reported that the COVID‐19 pandemic has substantially had a more severe effect on mental health, especially women’s mental health, and contributed to higher levels of anxiety and other anxiety‐related variables such as depressive disorders [33]. Indeed, social conditions such as labor, the market economy, and environmental degradation are pointed out as major challenges to improving women’s mental health, which may worsen with socioeconomic crises, as occurred during the COVID‐19 situation [34]. Thus, the rapid growth in the WCBA mental disorder burden could stem, at least partially, from the psychological distress imposed on reproductive age women by the traumatic COVID‐19 crisis across regions.

In 2021, the global estimated incidence of depressive disorders was reported as 77,722,841 cases, while anxiety disorders accounted for 18,962,131 estimated incident cases among WCBA. However, these numbers should be interpreted with caution due to the wide UI (93,343,266–65,414,928 for depressive disorders and 24,805,469–14,505,459 for anxiety disorders), which suggests substantial uncertainty in these estimates. The observed increase in ASRs from high SDI countries among WCBA may reflect demographic changes and inequities, such as population migration and racial discrimination, although these factors require further investigation to reveal their influence on the epidemiology of depressive and anxiety disorders [35, 36]. Recently, in some countries, there have been investments in mother and baby units, community mental health services, and specialist outpatient among WCBA, such as Melbourne in Australia [36]. Although these measurements have been conducted, challenges remain in mental health interventions among WCBA. These observations emphasize the need to continue enhancing and optimizing the investment structure of WCBA’s mental health intervention strategies to ensure increased intervention effectiveness.

Female disadvantage persists in social, cultural, and economic areas, which may cause a huge burden on WCBA mental health. Despite progress in gender equality, traditional gender norms, which assign women the primary role of caring for the family, persist in many regions (e.g., Italy) [37]. Meanwhile, with the advancement of society, there has been a significant shift in the role of women, with increased access to education, employment opportunities, and political participation. This situation may lead to a “double burden” phenomenon among WCBA [38], where women are given both work and family responsibilities (e.g., childbearing and parenting), leading to elevated levels of stress. In addition, cultural and educational factors may play a key role in WCBA mental health. Areas with more patriarchal cultural norms and lower levels of female education, such as the Chaoshan region in China [39] and Nepal [40], tend to have a higher incidence of gender‐based discrimination, and women have limited decision‐making power on reproductive health‐related issues, which may lead to mental health vulnerability. Conversely, areas (e.g., European countries) that promote gender equality and comprehensive sexuality education have been found to be associated with lower levels of depressive and anxiety disorders among WCBA [41]. Therefore, in order to reduce the burden of depressive and anxiety disorders among WCBA, there is a need for a comprehensive approach that addresses the underlying social, cultural, and economic underpinnings problems of mental health, depending on various regions.

Regionally, most low and low–middle SDI regions showed declining ASRs in depressive disorders, whereas stably increasing trends were revealed in anxiety disorders. This pattern is consistent with previous evidence that ASRs of depressive and anxiety disorders differ substantially between geographic regions [42, 43]. The declines of depressive disorders ASRs in lower‐income regions may stem from the settings with unavailable mental health services, along with lower detection rates and barriers, such as South Asia and Western and Eastern Sub‐Saharan Africa. Many crucial predisposing factors for anxiety disorders, such as gender inequity, poverty, and intimate partner violence, are common in low‐income countries, which are combined with reduced availability to mental health care [44, 45]. These situations often mean that women living in low‐income countries are at higher risk of developing anxiety disorders in WCBA [46]. Therefore, it’s justified to have an increased focus on depressive and anxiety disorders among WCBA living in low‐income countries [47].

Our age patterns analysis found that the 45–49 age group showed the highest global ASPR for depressive disorders increasing with age, while the 15–19 age group showed the highest ASIRs for anxiety disorders decreasing with age in 2021. Globally, more unfavorable period effects emerged before 2000 and unfavorable cohort effects in younger generations for depressive disorders. Most women experience menopause between the ages of 45 and 55 years as a natural component of the biological aging process [48]. There may be significant psychosocial factors, such as changes in the family structure and possibly retirement, which may occur at the moment that a woman reaches menopause [49]. A population‐based prospective study revealed a two‐fold increased risk of obvious depressive symptoms in women with perimenopause [50]. Besides, the heightened incidence in young women aged 15–19 years likely stems from puberty [51], representing a vulnerable developmental window for the development of anxiety disorders. Female puberty brings the initiation of menarche, accompanied by monthly steroid hormone fluctuations in the ovarian. Puberty may also be a time of increased psychosocial stress, such as university‐prompt achievement pressures and comparison with peers, which may contribute to the occurrence of anxiety symptoms [52]. For instance, in countries like South Korea, the phenomenon of “education anxiety” is particularly pronounced among adolescents and young adults [53]. Consequently, it is imperative to discern the high‐incidence age ranges with various mental disorders among WCBA, thereby facilitating the targeted and efficacious implementation. Policies, in order to enhance mental health services and welfare benefits specifically for WCBA, could serve as a vital step towards mitigating this burden.

With rising SDI, similar patterns resembling “W”‐shaped curves were shown in the ASRs of depressive disorders, and resembling “M”‐shaped curves were found in anxiety disorders among WCBA. Depressive disorders had the highest ASRs in higher SDI regions, while the highest ASRs in middle SDI regions existed in anxiety disorders. These regions and countries need to adapt their preventing or treating strategies in various mental disorders based on their specific ASRs. Furthermore, the ASRs for anxiety disorders demonstrated the most rapid increases in lower SDI regions. This suggests that lower SDI countries should focus on primary prevention as a cost‐effective strategy for long‐term mental disorder control.

Regarding the relationship between health‐related indexes and depressive and anxiety disorders, nursing and midwifery personnel rate (per 10,000) was positively associated with the age‐standardized incidence. This correlation suggests that enhanced access to healthcare resources may facilitate the early identification and intervention of WCBA depressive and anxiety disorders. Additionally, the negative correlation between incidence and the proportion of male nursing personnel confirms the importance of carefully optimizing the gender ratio among nursing staff, especially in the relevant departments of WCBA. It is noteworthy that the percentage of male nurses has increased by 59% over the past 10 years [54]. Although the numerical representation of the global incidence of anxiety among WCBA decreases as the proportion of male healthcare professionals increases, this may indicate an adverse effect of reduced early identification capabilities for anxiety. In the healthcare setting, a possible reason for this phenomenon could be that women of reproductive age may refuse to express symptoms of anxiety. The correlations between incidence and the healthcare, QoL, and ESG index confirm that mental health issues become more pronounced when a country has a lower combined risk in relation to the environment, human rights, and health and safety and fundamental living conditions are addressed.

However, our study has several limitations inherent to GBD studies. First, the estimations of mental disorders were more reliant on the quality and availability of the GBD 2021 data, lacking access to the original data for some low‐income countries and eventually limiting GBD from recording the estimates. Second, this study exclusively focused on describing the burdens of the top two mental disorders in detail: depressive and anxiety disorders, excluding other types. Third, the diagnostic criteria and detection protocols for mental disorders in the GBD system across countries may change over time, and the consistency across cultural contexts is uncertain. Potential data source biases and variable instrumentation may influence the ascertainment of patients. Given the above uncertainties, caution is needed in interpreting the burden trends in mental disorders identified in this study among WCBA. However, the GBD system compiled an extensive array of data sources, still making great progress in quantifying burdens of global diseases.

In conclusion, depression and anxiety disorders among WCBA pose a public health challenge globally. The ASPR, ASIR, and ASDR of depression and anxiety disorders among WCBA worldwide fluctuate and rise from 1990 to 2021, and rapid rises are found from 2019 to 2021. Despite downward trends in the global ASRs for depressive disorders in low‐income regions from 1990 to 2021, sociogeographic disparities in rates and trajectories persist. We found that the higher incidence burden of anxiety disorders was concentrated among younger age groups in WCBA. These insights highlight that healthcare providers should realize that social factors related to globalization may result in increasing burdens of WCBA being at risk for mental disorders. It was urgently needed for paying more attention to high‐risk demographic groups and implementing tailored primary prevention, secondary prevention, care innovations, and healthcare strategies. Moreover, it is indispensable to address the needs of WCBA based on disease type, age, region, and cultural context to reverse adverse patterns, particularly in aging societies.

## Funding

This study was supported by the Beijing Municipal Social Science Foundation (Grant 22JYA002).

## Disclosure

The founders had no role in the study design, data collection and analysis, decision to publish, or preparation of the manuscript.

## Ethics Statement

Ethical approval and informed consent were waived because the GBD is publicly available, and no identifiable information was included in the analyses. In accordance with the measures for ethical review of research in life sciences and medicine involving human beings, studies using legally obtained or publicly accessible human data could be exempt from ethical review if they pose no harm to individuals, do not involve sensitive, personal, or commercial information. This exemption is designed to reduce administrative burdens on researchers, while facilitating ethically sound human subjects research.

## Conflicts of Interest

The authors declare no conflicts of interest.

## Supporting Information

Additional supporting information can be found online in the Supporting Information section.

## Supporting information


**Supporting Information** Table S1 and Table S3 provide the information on the disease burden of mental disorders in WCBA at the national level. Table S2 and Table S4 provide additional details on changes in the burden of the top two mental disorders in WCBA in 204 countries. Table S5 and Table S6 provide the information on the global age structure of the top two mental disorders burden among WCBA in 2021. Table S7 and Table S10 provide additional details on temporal joinpoint analysis of the top two mental disorders ASPR from 1990 to 2021. Table S8 and Table S11 provide additional details on temporal joinpoint analysis of the top two mental disorders ASIR from 1990 to 2021. Table S9 and Table S12 provide additional details on temporal joinpoint analysis of the top two mental disorders ASDR from 1990 to 2021. Table S13 and Table S14 provide additional details on the health‐related variables in WCBA across the top 20 and bottom 20 countries and territories in 2021, sorted by 2021 ASIR of the top two mental disorders. Table S15 provides additional details on correlation between ASIR of depression and anxiety disorders and health‐related variables among WCBA in 2021. Figures S1 and S2 provide additional details on contributions and change in burden of mental disorders.

## Data Availability

The data that support the findings of this study are available from the corresponding author upon reasonable request.
