# Supplementary material for: Healthcare Services and Burden of the Top Two Mental Disorders Among Women of Childbearing Age Across 204 Countries and Territories, 1990–2021
Source: Depress Anxiety. 2026 Apr 1;2026:8872094. doi: 10.1155/da/8872094 (PMC13042365; doi:10.1155/da/8872094)
Supplement: Supplementary file 1 — Supporting Information Table S1 and Table S3 provide the information on the disease burden of mental disorders in WCBA at the national level. Table S2 and Table S4 provide additional details on changes in the burden of the top two mental disorders in WCBA in 204 countries. Table S5 and Table S6 provide the information on the global age structure of the top two mental disorders burden among WCBA in 2021. Table S7 and Table S10 provide additional details on temporal joinpoint analysis of the top two mental disorders ASPR from 1990 to 2021. Table S8 and Table S11 provide additional details on temporal joinpoint analysis of the top two mental disorders ASIR from 1990 to 2021. Table S9 and Table S12 provide additional details on temporal joinpoint analysis of the top two mental disorders ASDR from 1990 to 2021. Table S13 and Table S14 provide additional details on the health‐related variables in WCBA across the top 20 and bottom 20 countries and territories in 2021, sorted by 2021 ASIR of the top two mental disorders. Table S15 provides additional details on correlation between ASIR of depression and anxiety disorders and health‐related variables among WCBA in 2021. Figures S1 and S2 provide additional details on contributions and change in burden of mental disorders. [file DA-2026-8872094-s001.zip › Supplementary materials.docx]

**Table S1.** Prevalence, incidence and disability-adjusted life years of depressive disorders in women of child-bearing age at the national level, from 1990 to 2021.

|  | 1990 | | | | | 2021 | | | | |
| --- | --- | --- | --- | --- | --- | --- | --- | --- | --- | --- |
| Location | ASPR per 100 000  (95% uncertainty interval) | | ASIR per 100 000  (95% uncertainty interval) | | ASDR per 100 000  (95% uncertainty interval) | ASPR per 100 000  (95% uncertainty interval) | | ASIR per 100 000  (95% uncertainty interval) | | ASDR per 100 000  (95% uncertainty interval) |
| Afghanistan | 7679.79 (6259.54,9436.55) | 9341.51 (7360.57,12215.53) | | 1392.38 (899.9,1947.64) | | 9035.01 (6853.43,11791.33) | 11345.72 (8386.63,15574.45) | | 1671.5 (1049.55,2475.06) | |
| Albania | 3803.37 (3171.83,4720.54) | 3582.91 (2811.99,4638.06) | | 616.79 (410.7,876.15) | | 5081.76 (3982.93,6541.33) | 5307.71 (3753.55,7364.88) | | 866.77 (560.23,1318.48) | |
| Algeria | 6657.04 (5374.84,8445.08) | 7779.33 (5955.51,10409.62) | | 1200.3 (766.86,1744.58) | | 7663.55 (5956.17,10290.61) | 8992.85 (6496.65,12652.46) | | 1378.98 (819.19,2191.04) | |
| American Samoa | 3558.26 (2962.17,4348.83) | 2985.34 (2375.37,3843.55) | | 549.67 (359.16,769.92) | | 3995.62 (3176.44,5035.14) | 3393.49 (2469.5,4538.37) | | 614.74 (398.46,884.26) | |
| Andorra | 6608.08 (5529.46,8170.65) | 7540.3 (5962.53,9832.45) | | 1186.72 (781.87,1688.25) | | 7837.3 (5988.22,10390.09) | 9322.83 (6615.18,13163.46) | | 1429.67 (869.26,2191.19) | |
| Angola | 9001.82 (7289.59,11319.52) | 10928.11 (8463.89,14141.78) | | 1637.93 (1068.5,2340.89) | | 9779.78 (7552.76,13036.51) | 12119.07 (8810.48,16935.35) | | 1802.59 (1114.35,2671.56) | |
| Antigua and Barbuda | 4528.77 (3686.6,5691.16) | 5120.53 (3892.27,6843.33) | | 798.48 (502.72,1177.52) | | 5755.74 (4379.96,7657.29) | 6839.47 (4764.08,9824.07) | | 1039.7 (643.21,1635.19) | |
| Argentina | 4674.37 (3837.26,5777.94) | 5476.07 (4323.46,7121.27) | | 845.93 (556.38,1199.2) | | 5520.32 (4398.95,6833.14) | 6669.37 (5024.82,8496.85) | | 1015.66 (642.32,1445.85) | |
| Armenia | 3972.49 (3303.69,4896.19) | 3754.7 (2992.58,4815.1) | | 646.1 (427.39,935.87) | | 5271.38 (4083.2,6716.39) | 5520.48 (3935.1,7643.8) | | 899.94 (567.89,1371.63) | |
| Australia | 7788.83 (6785.36,8965.15) | 9233.93 (7784.53,10898.54) | | 1419.61 (983.07,1907.66) | | 8628.89 (6736.59,10954.38) | 10406.32 (7720.72,13813.7) | | 1590.49 (1042.76,2284.57) | |
| Austria | 6341.87 (5366.09,7606.08) | 7179.39 (5902.1,8928.22) | | 1129.2 (756.53,1552.7) | | 6391.11 (4901.48,8110.39) | 7231.03 (5231.26,9739.43) | | 1133.25 (707.68,1663.17) | |
| Azerbaijan | 3874.02 (3211.59,4787.17) | 3696.68 (2907.54,4838.32) | | 632.21 (412.81,911.05) | | 5047.65 (3995.09,6378.74) | 5233.36 (3675.99,7187.67) | | 856.67 (522.47,1279.48) | |
| Bahamas | 4523.56 (3690.61,5726.77) | 5135.03 (3967.1,6668.94) | | 798.46 (515.31,1150.44) | | 5858.55 (4398.6,7689.61) | 7052.45 (5026.53,9788.59) | | 1063.98 (637.83,1594.21) | |
| Bahrain | 8259.64 (6694.07,10385.6) | 9995.72 (7843.11,13175.11) | | 1519.33 (993.63,2167.26) | | 8984.47 (6745.07,11685.41) | 10967.08 (7725.58,15069.85) | | 1648.98 (1037.53,2468.65) | |
| Bangladesh | 7228.93 (5955.74,8863.15) | 8758.19 (7001.42,11634.16) | | 1315.97 (859.68,1860.06) | | 8265.4 (6366.85,10689.22) | 10171.1 (7398.47,13657.42) | | 1515.91 (921.56,2251.15) | |
| Barbados | 4577.44 (3730.04,5781.69) | 5153.92 (3962.2,6810.9) | | 807.04 (522.6,1167.41) | | 6045.79 (4631.16,8026.63) | 7262.82 (5145.84,10038.94) | | 1097.65 (662.87,1660.61) | |
| Belarus | 5640.33 (4692.88,6793.68) | 6188.13 (4996.08,7781.75) | | 984.52 (661.98,1393.44) | | 8244.04 (6377.48,10602.69) | 9946.47 (7123.64,13368.32) | | 1505.13 (932.66,2192.77) | |
| Belgium | 5703.73 (5144.66,6445.62) | 6474.95 (5781.12,7262.86) | | 1014.62 (721.33,1351.35) | | 7409.84 (5772.94,9320.97) | 9003.93 (6847.37,11924.16) | | 1359.69 (831.69,1936.46) | |
| Belize | 4420.81 (3622.71,5680.53) | 5097.38 (3928.34,6983.5) | | 787.13 (485.85,1151.85) | | 5672.62 (4342.7,7341.8) | 6852 (4873.07,9609.33) | | 1033.36 (665.94,1500.93) | |
| Benin | 5889.24 (4828.27,7350.95) | 6173.8 (4879.39,8199.61) | | 989.44 (644.18,1421.07) | | 6294.41 (4955.76,8252.6) | 6823.55 (4865.28,9580.91) | | 1082.2 (680.58,1656.1) | |
| Bermuda | 5520.31 (4501.37,6781.87) | 6418.1 (5024.83,8274.45) | | 997.55 (647.29,1393.26) | | 6042.1 (4531.65,7842.2) | 7181.9 (4862.9,10166.37) | | 1098.17 (657.92,1712.75) | |
| Bhutan | 5393.84 (4420.65,6643.44) | 6055.16 (4755.22,7872.12) | | 937.21 (617.37,1331.53) | | 5596.09 (4347.19,7261.11) | 6135.5 (4363.21,8556.43) | | 965.55 (595.94,1468.34) | |
| Bolivia (Plurinational State of) | 5383.58 (4456.64,6762.75) | 6447.15 (5142.26,8332.75) | | 978.43 (651.24,1403.89) | | 7046.26 (5369.46,9335.69) | 8869.97 (6470.95,12616.59) | | 1313 (803.08,1953.8) | |
| Bosnia and Herzegovina | 5168.76 (4281.14,6278.73) | 5475.77 (4416.31,6961.33) | | 893.82 (589.78,1248.37) | | 5609.55 (4322.21,7162.01) | 6019.16 (4290.02,8198.98) | | 966.66 (611.6,1473.25) | |
| Botswana | 5709.77 (4674.8,7134.21) | 5981.95 (4736.98,7923.87) | | 965.38 (642.73,1375.63) | | 7669.38 (5980.97,9938.14) | 8676.96 (6307.62,12181.2) | | 1333.88 (830.92,1935.66) | |
| Brazil | 6519.72 (5632.42,7631.32) | 8175.9 (6903.6,9768.49) | | 1202.75 (812.03,1654.03) | | 7816.37 (6664.34,9150.56) | 9932.83 (8192.6,12081.64) | | 1449.51 (963.31,1986.19) | |
| Brunei Darussalam | 2806.96 (2303.01,3533.57) | 2869.23 (2234.01,3819.95) | | 478.85 (308.5,700.98) | | 3160.63 (2499.2,4129.54) | 3354.11 (2389.66,4683.45) | | 548.68 (333.69,817.04) | |
| Bulgaria | 4631.01 (3858.14,5652.12) | 4611.01 (3753.63,5793.54) | | 770.97 (517.66,1076.01) | | 5793.67 (4542.75,7378.42) | 6259.78 (4476.1,8723.7) | | 998.68 (617.98,1441.55) | |
| Burkina Faso | 5737.02 (4688.87,7088.28) | 5962.41 (4749.75,7710.81) | | 962.68 (630.75,1375.47) | | 5798.57 (4540.02,7446.38) | 6075.12 (4449.68,8568.29) | | 982.91 (619.23,1505.21) | |
| Burundi | 7338 (6042.76,9100.57) | 7972.48 (6249.5,10276.39) | | 1266.37 (831.04,1795.79) | | 7117.88 (5377.17,9220.87) | 7690.85 (5299.01,10846.75) | | 1228.23 (747.86,1864.41) | |
| Cabo Verde | 5603.49 (4548.14,7087.68) | 5823.64 (4552.94,7883.33) | | 950.52 (623.21,1364.93) | | 7484.86 (5721.11,9665.07) | 8434.26 (6020.57,11647.97) | | 1323.29 (840.13,1998.42) | |
| Cambodia | 4541.02 (3797.76,5695.72) | 4419.59 (3487.32,5821.13) | | 748.85 (499.67,1054.36) | | 4706.26 (3674.08,5855.15) | 4464.69 (3230.77,5968.16) | | 771.46 (505.45,1132.18) | |
| Cameroon | 6067.69 (4955.29,7500.09) | 6476.65 (5171.8,8563.13) | | 1031.05 (681.7,1466.63) | | 6545.6 (5104.29,8502.95) | 7172.07 (5215.81,10220.49) | | 1132.04 (706.4,1666.52) | |
| Canada | 6239.93 (5346.67,7265.93) | 6469.96 (5545.24,7818.38) | | 1067.61 (741.31,1437.85) | | 7882.65 (6110.58,10170.66) | 8858.42 (6513.66,12034.06) | | 1403.65 (890.04,2060.16) | |
| Central African Republic | 8981.41 (7295.8,11412.03) | 10885.01 (8460.36,14088.49) | | 1619.67 (1038.67,2324.52) | | 9400.22 (7090.02,12346) | 11521.98 (8198.88,15957.05) | | 1714.96 (1087.16,2504.74) | |
| Chad | 6890.5 (5626.45,8600.54) | 7692.17 (6034.37,10169.15) | | 1202.91 (789.67,1735.79) | | 7034.38 (5296.13,9347.59) | 7992.65 (5709.27,10908.63) | | 1241.72 (776.85,1885.58) | |
| Chile | 8256.18 (7341.48,9438.53) | 10643.88 (9259.29,12566.52) | | 1586.6 (1107.04,2161.29) | | 8776.98 (6626.05,11766.81) | 11342.44 (8295.01,15672.04) | | 1680.87 (1047.5,2474.57) | |
| China | 4536.92 (3985.95,5211.11) | 4419.75 (3746.77,5272.36) | | 753.79 (510.88,1040.57) | | 4071.55 (3547.6,4728.99) | 3136.68 (2603.14,3769.61) | | 607.01 (415.92,823.82) | |
| Colombia | 3292.18 (2708.92,4060.3) | 3582.93 (2771.65,4665.87) | | 568.35 (375.63,804.25) | | 3243.33 (2536.97,4047.04) | 3419.7 (2432.18,4667.16) | | 550.72 (343.11,823.36) | |
| Comoros | 5791.17 (4721.33,7133.78) | 5707 (4483.24,7592.14) | | 953.24 (626.09,1350.55) | | 6601.1 (5180.67,8577.94) | 6785.55 (4832.91,9496.04) | | 1112.3 (700.19,1674.42) | |
| Congo | 8801.66 (7083.35,11152.62) | 10703.33 (8213.59,14008.96) | | 1597.13 (1037.15,2286.29) | | 9724.38 (7364.8,12942.97) | 11908.5 (8516.51,16732.42) | | 1776.95 (1115.45,2651.82) | |
| Cook Islands | 4263.5 (3477.58,5320.55) | 4003.04 (3006.67,5374.23) | | 694.48 (451.67,1020.26) | | 4857.16 (3792.99,6332.92) | 4630.77 (3235.96,6658.58) | | 795.55 (501.02,1211.63) | |
| Costa Rica | 4429.02 (3607.89,5478.78) | 5170.07 (3952.16,6811.4) | | 798.8 (507.5,1165.35) | | 5949.29 (4412.92,7858.76) | 7320.36 (5217.79,10296.76) | | 1100.55 (688.68,1653.79) | |
| Cite d'Ivoire | 5287.18 (4333.21,6556.75) | 5319.18 (4149.32,7097.72) | | 864.55 (581.89,1239.1) | | 5582.67 (4348.45,7007.25) | 5673.58 (4136.69,7844.38) | | 927.89 (580.54,1346.01) | |
| Croatia | 5208.45 (4340.61,6219.73) | 5468.88 (4435.02,6835.3) | | 894.2 (603.92,1225.73) | | 5323.34 (4174.2,6672.42) | 5593.6 (4125.74,7680.88) | | 908.62 (594.61,1321.66) | |
| Cuba | 8050.57 (6791.4,9601.85) | 10301.17 (8467.58,12708.57) | | 1529.41 (1042.38,2200.8) | | 6721.88 (5319.79,8844.28) | 8272.88 (6158.14,11540.2) | | 1237.43 (816.68,1822.37) | |
| Cyprus | 5667.86 (4694.61,7072.24) | 6181.49 (4856.97,8194.45) | | 993.19 (639.08,1430.21) | | 6566.37 (5070.67,8630.82) | 7422.55 (5262.5,10542.73) | | 1168.89 (712.25,1771.25) | |
| Czechia | 5144.56 (4297.12,6115.64) | 5388.19 (4341.62,6661.83) | | 876.97 (584.66,1223.67) | | 5608.72 (4379.45,7122.07) | 5955.79 (4304.47,7984.56) | | 960.71 (613.84,1386.92) | |
| Democratic People's Republic of Korea | 3937.95 (3286.17,4772.21) | 3474.34 (2830.94,4394.18) | | 625.03 (410,864.71) | | 4008.53 (3208.69,5030.73) | 3320.5 (2458.67,4416.66) | | 616.52 (397.48,881.13) | |
| Democratic Republic of the Congo | 8443.42 (6925.99,10500.25) | 10105.72 (7868.49,13179.17) | | 1502.77 (991.22,2171.12) | | 8677.9 (6639.23,11273.47) | 10448.95 (7535.79,14116.61) | | 1565.46 (989.08,2231.02) | |
| Denmark | 8104.13 (6784.07,9544.92) | 9782.39 (7977.99,11873.94) | | 1492.64 (1014.38,2068.87) | | 7284.77 (5647.48,9384.83) | 8600.35 (6401.12,11584.75) | | 1319.32 (831.45,1936.06) | |
| Djibouti | 6001.66 (4850.45,7534.31) | 6018.43 (4673.71,7987.13) | | 1000.39 (639.84,1448.87) | | 7042.2 (5502.74,8899.26) | 7332.06 (5287.17,9988.89) | | 1196.44 (735.41,1754.44) | |
| Dominica | 4426.13 (3605.97,5656.79) | 5052.43 (3903.75,6811.94) | | 781.9 (499.06,1137.46) | | 5728.5 (4281.13,7612.92) | 6888.37 (4893.06,9600.84) | | 1039.12 (622.44,1640.85) | |
| Dominican Republic | 5510.08 (4466.12,7000.13) | 6671.41 (5133.65,9054.46) | | 1011.06 (635.26,1474.38) | | 6638.74 (4931,8776.93) | 8229.48 (5780.76,11524.79) | | 1230.74 (727.74,1905.06) | |
| Ecuador | 4920.11 (4045.44,6053.9) | 5728.01 (4520.45,7342.07) | | 889.4 (585.47,1266.75) | | 6363.03 (4963.32,8187.71) | 7761.94 (5663.91,10417.22) | | 1176.75 (719.78,1725.56) | |
| Egypt | 6227.68 (5048.03,7740.98) | 7048.93 (5451.31,9407.99) | | 1100.57 (718.54,1594.87) | | 7514.5 (5701.74,9814.76) | 8904.9 (6313.95,12203.19) | | 1362.27 (843.59,2012.33) | |
| El Salvador | 6089.71 (4964.06,7477) | 7659.22 (6020.17,9765.8) | | 1144.99 (749.34,1634.74) | | 6461.05 (4909.22,8563.78) | 8088.73 (5886.45,11113.72) | | 1213.27 (734.23,1866.57) | |
| Equatorial Guinea | 9083.41 (7421.09,11459.63) | 11042.87 (8597.18,14392.32) | | 1639.5 (1054.63,2352.79) | | 9608.97 (7428.94,12685.68) | 11804.79 (8782.27,16340.51) | | 1759.16 (1121.2,2674.8) | |
| Eritrea | 6598.64 (5446.02,8026.89) | 6880.95 (5347.12,8683.51) | | 1110.85 (709.87,1580.39) | | 7044.31 (5516.08,9006.26) | 7487.99 (5480.45,10340.33) | | 1209.59 (729.2,1777.05) | |
| Estonia | 7008.07 (5845.76,8410.61) | 8159.31 (6571.02,10127.03) | | 1265.35 (846.29,1765.41) | | 6948.87 (5391.56,8880.01) | 8049.93 (5794.27,10719.08) | | 1242.43 (798.15,1811.24) | |
| Eswatini | 5672.07 (4644.93,6930.78) | 5929.56 (4658.58,7626.86) | | 962.84 (635.86,1375.57) | | 8533.02 (6475.21,11160.57) | 10133.06 (7137.6,14027.13) | | 1520.27 (932.87,2280.99) | |
| Ethiopia | 6459.03 (5563.17,7633.77) | 6538.82 (5406.57,8108.06) | | 1072.53 (718.21,1498.17) | | 6447.58 (5447.67,7711.15) | 6723.33 (5355.64,8487.21) | | 1090.44 (729.1,1544.84) | |
| Fiji | 3995.05 (3321.88,4819.44) | 3552.31 (2882.32,4601.32) | | 632.34 (419.26,887.63) | | 4633.33 (3684.12,5848.39) | 4331.24 (3203.69,5891.97) | | 750.74 (486.97,1131.91) | |
| Finland | 8829.73 (7457.2,10452.77) | 10459.28 (8569.76,12932.72) | | 1623.25 (1099.63,2245.79) | | 9163.62 (7264.56,11545.38) | 11020.19 (8148.23,14753.34) | | 1690.72 (1130.43,2465.61) | |
| France | 7960.01 (7217,8822.7) | 9534.1 (8524.94,10709.26) | | 1463.58 (1030.19,1967.45) | | 8396.09 (6508.99,10849.3) | 10237.1 (7634.06,13970.63) | | 1548.65 (1006.53,2251.85) | |
| Gabon | 8179.21 (6634.18,10200.82) | 9738.84 (7510.56,12755.93) | | 1467.56 (939.58,2108.69) | | 9199.61 (6987.85,12041.6) | 11172.48 (8089.09,15286.46) | | 1670.13 (1030.56,2564.26) | |
| Gambia | 8945.86 (7327.75,11184.71) | 10769.68 (8488.67,14285.87) | | 1634.03 (1067.4,2357.49) | | 9470.39 (7223.38,12257.12) | 11554.49 (8090.56,16006.6) | | 1738.38 (1030.27,2585.86) | |
| Georgia | 4366.93 (3630.01,5352.93) | 4303.3 (3390.92,5541.31) | | 725.76 (480.72,1043.44) | | 5589.06 (4281.39,7125.62) | 6010.29 (4283.9,8242.15) | | 963.87 (607.09,1416.14) | |
| Germany | 5247.22 (4573.82,6067.22) | 5682.61 (4883.37,6735.22) | | 908.07 (629.06,1220.89) | | 6999.86 (5404.19,9227.5) | 8354.19 (6112.69,11243.09) | | 1271.89 (814.19,1854.57) | |
| Ghana | 5902.31 (4863.52,7350.39) | 6197.17 (4757.86,7987.96) | | 996.57 (634.96,1443.01) | | 6477.29 (5063.46,8364.26) | 6970.43 (5013.46,9725.46) | | 1112.72 (715,1733.9) | |
| Greece | 8721.5 (7037.27,11062.61) | 10657.62 (8357.39,13969.96) | | 1628.18 (1017.73,2357.6) | | 10867.51 (8014.92,14608.04) | 13752.23 (9556.08,19457.66) | | 2056.87 (1242.93,3121.65) | |
| Greenland | 14321.84 (11791.88,17634.68) | 18096.84 (14525.33,22616.78) | | 2740.72 (1880.87,3881.59) | | 15638.17 (12258.72,19916.72) | 20143.8 (15104.28,26617.65) | | 3014.3 (1956.14,4524.11) | |
| Grenada | 4504.07 (3656.19,5772.32) | 5163.33 (3969.15,6964.36) | | 798.13 (497.97,1148.59) | | 5744.88 (4396.7,7337.03) | 6887.82 (4831.77,9367.64) | | 1042.39 (659.62,1594.19) | |
| Guam | 4117.42 (3387.4,5008.6) | 3670.08 (2990.21,4604.21) | | 658.6 (441.87,926.05) | | 4819.67 (3920.9,5998.78) | 4540.07 (3418.54,6180.84) | | 788.78 (520.67,1134.25) | |
| Guatemala | 5225.53 (4230.39,6739.76) | 6428.22 (4959.19,8535.9) | | 952.2 (607.03,1377.07) | | 6591.44 (4911.49,9090.74) | 8397.12 (6076.08,12161.6) | | 1231 (766.77,1919.4) | |
| Guinea | 6126.1 (5048.16,7579.71) | 6465.01 (5099.08,8498.15) | | 1036.87 (664.26,1463.22) | | 6602.89 (5223.84,8534.27) | 7264.92 (5297.83,10153.76) | | 1145.58 (719.52,1726.89) | |
| Guinea-Bissau | 6097.65 (4995.66,7684.95) | 6528 (5160.31,8568.48) | | 1039.08 (664.03,1494.46) | | 6760.37 (5238.83,8654.1) | 7479.23 (5380.56,10290.86) | | 1172.72 (727.93,1769.53) | |
| Guyana | 6828.58 (5541.03,8195.63) | 8596.44 (6738.67,10717.62) | | 1274.5 (847.1,1824.76) | | 9981.22 (7623.71,13289.62) | 13148.63 (9710.99,18438.78) | | 1904.6 (1190.54,2839.01) | |
| Haiti | 5634.61 (4559.86,7207.74) | 6821.77 (5322.35,9112.99) | | 1016.24 (654.92,1459.74) | | 6524.39 (4953.99,8557.09) | 8078.91 (5689.64,10990.36) | | 1191.63 (734.07,1791.82) | |
| Honduras | 4185.92 (3419.91,5340.38) | 4890.7 (3775.64,6436.62) | | 749.2 (484.83,1096.97) | | 5700.83 (4235.88,7430.88) | 7102.86 (4976.47,9718.87) | | 1053.46 (625.3,1566.35) | |
| Hungary | 5372.18 (4573.74,6444.06) | 5710.17 (4647.8,7065.47) | | 921.23 (617.45,1287.21) | | 5212.11 (4132.55,6514.93) | 5401.59 (3953.37,7117.7) | | 880.71 (548.64,1277.59) | |
| Iceland | 5904.83 (4960.98,7094.53) | 6556.71 (5325.02,8314.03) | | 1043.65 (691.72,1465.45) | | 5789.07 (4471.05,7329.75) | 6348.68 (4541.52,8705.07) | | 1014.31 (645.86,1470.01) | |
| India | 5832.45 (5076.22,6779.12) | 6621.22 (5521.99,8058.32) | | 1011.3 (681.11,1394.93) | | 6036.84 (5198.69,7020.24) | 6763.66 (5627.25,8221.86) | | 1046.72 (711.21,1435.86) | |
| Indonesia | 3389.44 (2929.13,3948.83) | 2721.14 (2238.53,3323) | | 513.09 (350.46,710.99) | | 4235.79 (3638.56,4949.06) | 3655.59 (2975.11,4517.08) | | 664.3 (449.36,930.26) | |
| Iran (Islamic Republic of) | 8385.13 (7104.38,10297) | 10224.13 (8222.6,12877.42) | | 1537.5 (1018.18,2201.72) | | 10123.93 (8509.47,12287.18) | 12341.05 (9856.49,15351.78) | | 1853.6 (1217.27,2675.24) | |
| Iraq | 6475.03 (5329.33,7883.1) | 7521.04 (5927.41,9559) | | 1154.57 (761.91,1626.13) | | 7167.29 (5572.88,9268.11) | 8431.91 (6188.89,11877.04) | | 1280.08 (808.49,1912.06) | |
| Ireland | 6798.45 (5816.53,7967.21) | 8030.99 (6709.38,9711.69) | | 1236.46 (836.92,1702.64) | | 9120.29 (7029.27,11621.66) | 11372.9 (8554.76,15060.21) | | 1703.06 (1096.33,2523.24) | |
| Israel | 7433.08 (6107.33,9184.65) | 8689.18 (6882.92,11172.98) | | 1356.11 (895.48,1909.91) | | 8290.31 (6340.07,10836.06) | 9932.98 (7242.26,13812.4) | | 1530.14 (950.73,2330.93) | |
| Italy | 6643.54 (5684.66,7829.44) | 7685.99 (6326.86,9536.03) | | 1193.83 (791.6,1684.44) | | 8155.51 (6916.93,9687.98) | 9837.48 (7964.04,12230.92) | | 1500.62 (985.49,2134.96) | |
| Jamaica | 4450.17 (3617.25,5644.12) | 5092.94 (3876.23,6849.53) | | 790.29 (507.13,1174.75) | | 5868.79 (4390.02,7784.21) | 7078.76 (4962.41,10046.16) | | 1070.84 (656.66,1656.6) | |
| Japan | 3441.73 (3046.43,3883.28) | 3758.26 (3246.86,4407.12) | | 605.1 (414.33,829.51) | | 4379.19 (3847.01,5058.9) | 5086.27 (4357.15,5973.17) | | 798.17 (545.5,1084.59) | |
| Jordan | 7321.36 (5903.64,9241.9) | 8821.61 (6922.38,11660.94) | | 1340.65 (876.12,1944.78) | | 7895.86 (6008.98,10627.61) | 9490.74 (6857.12,13502.89) | | 1437.88 (892.81,2229.89) | |
| Kazakhstan | 4687.59 (3886.45,5626.8) | 4822.67 (3939.19,5914.67) | | 794.09 (527.98,1098.87) | | 5708.4 (4425.01,7006.92) | 6205.12 (4572.26,8132.82) | | 994.89 (662.67,1468.83) | |
| Kenya | 6348.81 (5494.84,7439.13) | 6689.19 (5607.83,8178.15) | | 1077.92 (732.11,1476.36) | | 6734.46 (5781.37,7872.02) | 7141.81 (5898.94,8763.22) | | 1147.38 (762.93,1588.85) | |
| Kiribati | 4102.01 (3401.76,4970.05) | 3750.36 (3001.31,4853.83) | | 654.93 (437.44,913.39) | | 4169.24 (3292.69,5185.23) | 3733.73 (2782.08,5007.48) | | 658.18 (438.2,958.35) | |
| Kuwait | 6866.81 (5556.38,8626.81) | 7945.7 (6130.9,10492.84) | | 1234.06 (785.89,1819.92) | | 7254.47 (5507.41,9602.42) | 8221.05 (5713.82,11902.98) | | 1281.21 (781.42,1984.43) | |
| Kyrgyzstan | 4704.04 (3863.9,5675.74) | 4960.79 (3976.38,6223.99) | | 806.6 (539.8,1135.96) | | 5710.03 (4484.61,7167.83) | 6332.12 (4719,8380.07) | | 1005.24 (618.59,1484.45) | |
| Lao People's Democratic Republic | 3849.64 (3228.54,4733.64) | 3432.72 (2729.62,4425.86) | | 608.94 (407.92,866.42) | | 3894.58 (3074.41,4914.29) | 3304.62 (2373.53,4498.24) | | 607.79 (382.18,873.06) | |
| Latvia | 6193.87 (5113.15,7475.14) | 6973.28 (5584.92,8733.93) | | 1094.59 (730.93,1509.62) | | 7218.57 (5498.02,9336.41) | 8466.41 (5974.98,11527.89) | | 1296.62 (790.94,1913.18) | |
| Lebanon | 6820.64 (5691.25,8353.17) | 7885.39 (6305.53,10327.17) | | 1213.3 (799.77,1753.96) | | 10416.51 (7825.02,13597.53) | 13084.24 (9180.35,17818.15) | | 1931.23 (1186.7,2897.64) | |
| Lesotho | 8587.97 (7178.19,10673.26) | 10119.81 (8076.55,13012.13) | | 1556.04 (1020.71,2230.28) | | 10798.47 (8396.93,13983.26) | 13484.98 (10055.58,18236.7) | | 1977.19 (1225.3,2883.87) | |
| Liberia | 7202.53 (5919.12,8998.37) | 8147.06 (6421.6,10663.64) | | 1238.87 (813.39,1776.03) | | 7591.76 (5810.51,9808.8) | 8696.41 (6406.81,12225) | | 1321.5 (808.59,2015.79) | |
| Libya | 6753.53 (5459.02,8579.32) | 7980.79 (6201.45,10744.98) | | 1225.68 (787.62,1784.51) | | 8080.83 (6284.8,10463.73) | 9623.16 (6864.28,13320.19) | | 1457.18 (908.39,2247.45) | |
| Lithuania | 6261.91 (5243.84,7461.73) | 7105.25 (5812.28,8956.87) | | 1113.18 (764.14,1533.65) | | 8191.06 (6325.87,10375.54) | 9924.65 (7354.6,13505.44) | | 1499.53 (939.86,2213.81) | |
| Luxembourg | 6981.89 (5821.97,8399.14) | 8069.69 (6451.16,10190.35) | | 1259.5 (836.5,1733.88) | | 6550.34 (5107.72,8151.27) | 7442.21 (5521.67,9591.5) | | 1165.76 (748.04,1678.6) | |
| Madagascar | 6384.66 (5214.05,7877.16) | 6583.58 (5258.28,8628.25) | | 1070.9 (676.55,1529.02) | | 7026.3 (5432.09,8985.29) | 7528.41 (5324.07,10522.08) | | 1207.08 (724.67,1825.24) | |
| Malawi | 5884.35 (4757.75,7187.07) | 5832.07 (4504.6,7608.96) | | 952.43 (623.88,1336.84) | | 6276.11 (4882.94,8081.02) | 6464.29 (4646.65,8944.9) | | 1050.75 (678.06,1551.29) | |
| Malaysia | 4158.94 (3452.53,5116.58) | 3798.32 (3052.04,4895.37) | | 670.61 (446.84,947.98) | | 4902.33 (3854.5,6203.63) | 4722.72 (3349.75,6499.8) | | 812.11 (514.78,1202.13) | |
| Maldives | 5407.58 (4520.97,6645.33) | 5863 (4703.49,7544.48) | | 944.59 (627.3,1323.92) | | 5095.42 (4117,6409.05) | 4853.42 (3555.51,6607.29) | | 834.16 (547.11,1220.74) | |
| Mali | 5195.83 (4259.49,6454.61) | 5132.69 (4065.16,6628.38) | | 846.45 (554.29,1207.82) | | 5181.65 (4052.62,6655.33) | 5198.65 (3767.24,7169.56) | | 854.05 (546.73,1259.62) | |
| Malta | 5677.88 (4635.54,7024.93) | 6174.91 (4757.47,7959.2) | | 991.74 (643.01,1454.51) | | 6463.67 (4976.36,8425.95) | 7292.11 (5183.73,10139.07) | | 1146.94 (714.39,1709.54) | |
| Marshall Islands | 3832.16 (3190.36,4709.24) | 3503.54 (2784.55,4668.1) | | 614.14 (406.93,885.08) | | 4235.06 (3374.82,5256.59) | 3809.17 (2806.69,5153.13) | | 670.12 (420.16,975.9) | |
| Mauritania | 4875.5 (4059.41,6043.34) | 4680.21 (3711.05,6216.24) | | 787.88 (524.92,1125.04) | | 5091.43 (3967.98,6527.09) | 5007.38 (3638.53,7072.06) | | 837.88 (531.49,1268.29) | |
| Mauritius | 6778.98 (5616.74,8124.67) | 7462.65 (5998.84,9324.07) | | 1211.86 (795.77,1721.53) | | 7293.97 (5606.49,9310.87) | 8008.13 (5802.33,10844.51) | | 1297.51 (803.58,1929.6) | |
| Mexico | 4345.2 (3749,5110.46) | 5031.46 (4156.02,6132.86) | | 773.35 (525.73,1076.32) | | 7772.01 (6598.98,9153.64) | 10014.53 (8172.59,12124.46) | | 1464.09 (976.61,2022.38) | |
| Micronesia (Federated States of) | 3929.58 (3246.63,4810.23) | 3628.31 (2895.08,4744.28) | | 632.2 (413,895.8) | | 4161.62 (3286.01,5287.79) | 3773.71 (2800.1,5025.16) | | 664.86 (428.34,968.3) | |
| Monaco | 7266.8 (5732.26,9178.03) | 8450.32 (6297.3,11264.95) | | 1316.05 (849.54,1960.96) | | 8836.15 (6590.49,11767.32) | 10830.51 (7536.47,15562.01) | | 1637.25 (977.79,2445.53) | |
| Mongolia | 5616.09 (4634.24,6859.57) | 6394.18 (5105.02,8178.14) | | 1002.01 (646.53,1424.21) | | 6006.35 (4823.88,7745.78) | 6651.11 (4888.69,9040.47) | | 1058.03 (675.6,1548.44) | |
| Montenegro | 4458.35 (3710.41,5314.97) | 4442.35 (3591.24,5563.71) | | 745.51 (486.21,1050.48) | | 5754.23 (4486.68,7423.7) | 6284.28 (4548.06,8638.25) | | 999.32 (637.16,1491.41) | |
| Morocco | 7978.37 (6501.02,9977.74) | 9686.37 (7683.75,12635.06) | | 1464.43 (947.38,2095.09) | | 9463.34 (7353.16,12411.16) | 11733.34 (8531.25,15968.75) | | 1746.54 (1119.15,2558.69) | |
| Mozambique | 6373.6 (5237.92,7859.27) | 6521.95 (5150.76,8654.88) | | 1051.58 (681.05,1487.43) | | 7283.57 (5642.7,9571.04) | 7980.01 (5594.73,11486.72) | | 1231.55 (785.39,1860.84) | |
| Myanmar | 2838.85 (2268.6,3484.45) | 1921.57 (1491.63,2492.68) | | 396.79 (262.2,567.91) | | 3544.08 (2834.63,4417.7) | 2721.47 (1966.01,3781.24) | | 525.38 (344.54,776.98) | |
| Namibia | 5015.36 (4128.62,6174.75) | 4934.62 (3930.9,6470.02) | | 821.08 (533.24,1157.16) | | 6621.47 (5139.94,8514.13) | 7222.63 (5234.88,10109.03) | | 1139.94 (732.99,1705.49) | |
| Nauru | 4246.22 (3427.44,5310.17) | 3961.63 (2945.99,5318.75) | | 687.21 (454.85,1002.56) | | 4709.74 (3657.78,6059.01) | 4611.94 (3229.21,6582.26) | | 779.62 (490.82,1157.8) | |
| Nepal | 5932.98 (4896.65,7235.64) | 6759.86 (5329.98,8633) | | 1032.21 (685.55,1463.99) | | 8166.63 (6214.44,10577.27) | 10091.67 (7108.17,13772.77) | | 1497.15 (940.07,2296.71) | |
| Netherlands | 6569.67 (5898.73,7362.42) | 7664.25 (6792,8680.17) | | 1190.82 (835.51,1595.79) | | 7611.16 (5903.83,9888.03) | 9185.26 (6661.82,12483.33) | | 1402.13 (887.4,2095.16) | |
| New Zealand | 6656.55 (5638.48,8062.78) | 7505.43 (6054.69,9524.49) | | 1170.23 (773.89,1652.08) | | 7066.92 (5685.38,8807.39) | 8055.97 (6131.59,10607.99) | | 1262.64 (834.75,1795.96) | |
| Nicaragua | 4793.99 (3940.36,5984.94) | 5776.79 (4551.43,7614.74) | | 876 (566.34,1262.08) | | 5987.26 (4544.28,7677.48) | 7411.34 (5246.44,10054.96) | | 1112.08 (733,1647.72) | |
| Niger | 5873.14 (4801.99,7212.34) | 6198.85 (4870.75,7930.89) | | 994.06 (644.45,1416.21) | | 5663.34 (4471.68,7265.26) | 5984.75 (4304.44,8126.73) | | 965.46 (642.85,1472.59) | |
| Nigeria | 5490.03 (4705.02,6482.91) | 5733.21 (4706.17,7166.2) | | 915.82 (609.2,1274.36) | | 5018.93 (4318.1,5854.67) | 4940.76 (4021.97,6104.69) | | 820.2 (548.26,1141.78) | |
| Niue | 4340.73 (3546.35,5415.12) | 3977.25 (2988.01,5214.1) | | 699.4 (465.65,1014.84) | | 4864.26 (3790.37,6227.65) | 4601.21 (3242.69,6358.56) | | 791.7 (501.52,1179.59) | |
| North Macedonia | 4215.69 (3535.57,5139.03) | 4076.37 (3307.63,5149.26) | | 690.43 (461.35,960.3) | | 5428.83 (4283.64,6875.35) | 5743.13 (4205.49,7703.11) | | 929.74 (597.33,1329.2) | |
| Northern Mariana Islands | 3507.63 (2821.59,4303.93) | 2823.96 (2251.05,3723.05) | | 532.36 (352.41,765.56) | | 4378.41 (3502.91,5504.48) | 3777.47 (2793.7,5278.76) | | 685.05 (445.24,1011.93) | |
| Norway | 6352.24 (5468.21,7495.95) | 7105.63 (5845.79,8739.55) | | 1128.86 (744.28,1548.5) | | 7649 (6367.38,9129.95) | 8984.66 (7128.64,11239.5) | | 1394.31 (914.46,1959.75) | |
| Oman | 6501.93 (5272.69,8094.04) | 7479.1 (5838.76,9678) | | 1159.39 (743.81,1703.5) | | 7928.14 (6069.12,10566.72) | 9398.95 (6458.57,13344.93) | | 1433.76 (865.96,2123.05) | |
| Pakistan | 5636.76 (4843.41,6715.96) | 6358.9 (5174.2,7921.54) | | 978.48 (644.79,1385.09) | | 6286.18 (5263.33,7601.98) | 7236.46 (5703.67,9267.01) | | 1103.25 (713.93,1577.88) | |
| Palau | 4303.24 (3506.7,5335.24) | 3985.47 (3002.81,5303.37) | | 695.5 (455.71,1006.48) | | 5006.8 (3945.42,6545.21) | 4611.04 (3255.93,6379.67) | | 803.92 (512.75,1221.67) | |
| Palestine | 8641.92 (6986.97,10962.69) | 10729.1 (8224.42,14243.45) | | 1610.83 (1042.41,2352.09) | | 10067.64 (7622.61,13290.53) | 12747.11 (9150.79,17869.97) | | 1894.27 (1152.18,2858.48) | |
| Panama | 4217.76 (3425.17,5314.25) | 4876.98 (3739.75,6373.1) | | 754.57 (477.58,1086.12) | | 5406.24 (4014.59,7064.97) | 6570.74 (4546.04,9099.34) | | 991.06 (619.27,1505.12) | |
| Papua New Guinea | 4361.16 (3567.49,5431.46) | 4198.28 (3313.44,5520.11) | | 713.33 (471.53,1003.47) | | 4435.25 (3534.54,5560.15) | 4140.38 (3034.34,5652.36) | | 716.79 (438.73,1042.58) | |
| Paraguay | 5305.87 (4371.99,6589.35) | 6274.18 (5016.72,8258.13) | | 957.06 (623.81,1356.83) | | 7238.18 (5516.54,9531.9) | 9071.65 (6562.46,12574.91) | | 1344.7 (833.49,1994.46) | |
| Peru | 3468.88 (2845.33,4276.71) | 3600.12 (2807.08,4634.57) | | 584.01 (388.65,816.05) | | 4360.47 (3390.43,5548.28) | 4810.92 (3475.93,6663.86) | | 757.48 (481.88,1127.87) | |
| Philippines | 4042.31 (3473.72,4734.24) | 3758.74 (3085.66,4618.42) | | 650.71 (441.09,906.14) | | 4649.58 (3993.17,5489.09) | 4471.7 (3678.58,5561.74) | | 763.26 (513.44,1064.59) | |
| Poland | 3390.44 (2923.9,3947.05) | 2752.6 (2231.87,3390.21) | | 509.49 (347.27,700.99) | | 4012.66 (3445.55,4707.08) | 3532.65 (2777.31,4348.03) | | 627.78 (417.74,885.11) | |
| Portugal | 9232.08 (7654.43,11191.31) | 11407.12 (9171.45,14322.71) | | 1730.04 (1146.76,2402.16) | | 10380.37 (7807.85,13855.54) | 13067.76 (9520.23,18448.87) | | 1953.71 (1191.8,2908.59) | |
| Puerto Rico | 3929.14 (3263.62,4752.57) | 4180.24 (3297.57,5335.7) | | 671.33 (448.8,959.52) | | 4700.99 (3665.57,6047.94) | 5263.47 (3777.09,7330.64) | | 822.88 (494.68,1219.53) | |
| Qatar | 7664.9 (6269.96,9412.55) | 9066.29 (7112.28,11627.95) | | 1386.97 (878.27,2015.85) | | 8021.93 (6127.86,10606.95) | 9415.42 (6648.95,13312.04) | | 1441.97 (895.78,2216.9) | |
| Republic of Korea | 3492.03 (2998.83,4009.21) | 3737.67 (3159.46,4416.07) | | 608.98 (409.28,830.98) | | 3987.11 (3189.87,4933.13) | 4377.76 (3283.92,5643.59) | | 705.97 (451.66,1015.22) | |
| Republic of Moldova | 5335.35 (4444.42,6349.4) | 5741.99 (4552.34,7176) | | 920.53 (622.07,1290.89) | | 6008.84 (4645.11,7758.91) | 6613.15 (4710.41,9257.02) | | 1044.49 (658.46,1546.53) | |
| Romania | 4127.57 (3424.91,4948.27) | 3952.78 (3143.35,4994.43) | | 671.23 (436.51,943.72) | | 5226.37 (4096.1,6612.44) | 5445.42 (3970.15,7265.31) | | 884.84 (569.54,1282.96) | |
| Russian Federation | 4724.97 (4036.26,5584.63) | 4793 (3860,5934.41) | | 787.35 (519.64,1105.9) | | 5995.06 (5100.7,7047.7) | 6528.29 (5253.17,7989.13) | | 1035.41 (686.49,1450.26) | |
| Rwanda | 7916.21 (6542.9,9971.93) | 8871.42 (7008.63,11855.26) | | 1394.66 (900.09,1985.44) | | 8128.17 (6317.08,10467.65) | 9130.08 (6519.84,12335.53) | | 1435.54 (882.46,2198.22) | |
| Saint Kitts and Nevis | 5504.67 (4345.32,7256.37) | 6666.29 (4934.83,9210.73) | | 1004.19 (638.13,1504.61) | | 7088.05 (5174.62,9720.4) | 8829.62 (5923.54,12854.92) | | 1317.11 (811.49,1962.39) | |
| Saint Lucia | 4501.02 (3661.21,5673.08) | 5175.32 (3994.22,6821.29) | | 797.13 (515.84,1154.43) | | 6269.95 (4736.82,8308.69) | 7602.25 (5413.2,10849.18) | | 1141.59 (699.75,1704.57) | |
| Saint Vincent and the Grenadines | 4440.61 (3625.14,5641.2) | 5107.68 (3942.26,6878.76) | | 789.32 (490.28,1136.93) | | 5924.63 (4506.2,7771.92) | 7132.98 (5048.06,10126.22) | | 1074.85 (667.16,1626.33) | |
| Samoa | 3794.44 (3166.08,4588.47) | 3455.05 (2752.86,4502.87) | | 608.89 (404.19,866.65) | | 4049.84 (3129.21,5085.92) | 3648.96 (2653.71,5045.51) | | 645.66 (406.95,955.82) | |
| San Marino | 7162.03 (5785.47,9220.16) | 8406.26 (6345.44,11333.22) | | 1305.01 (836.69,1948.15) | | 8947.82 (6639.46,12010.67) | 11001.4 (7561.83,15750.7) | | 1663.3 (995.1,2729.67) | |
| Sao Tome and Principe | 5311.17 (4386.25,6609.79) | 5394.32 (4189.55,7031.78) | | 890.88 (588.52,1274.01) | | 5864.14 (4665.32,7376.23) | 6094.9 (4387.48,8370.39) | | 995.94 (621.55,1465.06) | |
| Saudi Arabia | 6504.86 (5255.8,8237.71) | 7511.01 (5744.48,9943.38) | | 1161.94 (734.43,1705.41) | | 7811.87 (6045.6,10237) | 9171.29 (6331.32,12797.03) | | 1405.2 (878.94,2097.45) | |
| Senegal | 5116.13 (4199.24,6332.7) | 5077.02 (3995.12,6832.14) | | 835.74 (557.08,1177.66) | | 5957.66 (4677.14,7748.36) | 6287.78 (4642.51,8804.17) | | 1007.39 (624.19,1470.79) | |
| Serbia | 4774.67 (4035.41,5748.34) | 4869.03 (3989.42,6130.75) | | 806.3 (540.83,1101.19) | | 5236.11 (4111.21,6669.34) | 5497.94 (3951.04,7706.69) | | 893.53 (571.6,1344.49) | |
| Seychelles | 3453.19 (2809.09,4219.89) | 2843.28 (2266.21,3675.07) | | 531.24 (354,747.12) | | 4392.48 (3482.35,5522.99) | 3796.75 (2693.65,5307.27) | | 690.36 (434.4,1079.14) | |
| Sierra Leone | 5861.84 (4800.5,7251.29) | 6111.16 (4798.29,7948.05) | | 987.34 (650.29,1400.73) | | 6225.99 (4876.54,8042.82) | 6703.41 (4846.47,9220.7) | | 1070.83 (665.07,1659.48) | |
| Singapore | 5293.72 (4601.53,6111.55) | 6553.64 (5561.72,7772.89) | | 1008.27 (689.51,1374.93) | | 3448.16 (2725.13,4374.47) | 3874.88 (2935.26,5115.96) | | 621.28 (401.19,908.05) | |
| Slovakia | 4490.66 (3749.24,5364.65) | 4460.43 (3584,5621.26) | | 745.8 (504.41,1063.25) | | 5581.35 (4340.14,7299.19) | 5941.41 (4298.07,8302.76) | | 958.43 (606.22,1460.89) | |
| Slovenia | 5984.68 (5044.36,7099.17) | 6623.05 (5419.47,8036.45) | | 1058.02 (711.03,1461.77) | | 5978.4 (4620.33,7611.06) | 6519.14 (4761.68,8842.13) | | 1039.07 (680,1594.19) | |
| Solomon Islands | 4137.67 (3421.56,5164.7) | 4002.2 (3146.4,5264.79) | | 680.6 (445.98,970.99) | | 4445.55 (3523.19,5610.69) | 4202.69 (3066.04,5767.55) | | 722.91 (466.15,1031.01) | |
| Somalia | 6772.36 (5587.86,8267.05) | 7103.72 (5548.18,9225.17) | | 1137.58 (743.89,1599.3) | | 7893.2 (6182.81,10115.18) | 8908.52 (6387.41,12045.86) | | 1385.93 (851.59,2037.41) | |
| South Africa | 6379.37 (5571.01,7317.25) | 6967.5 (5903.18,8378.54) | | 1095.64 (753.82,1496.93) | | 8248.65 (7094.41,9591.96) | 9555.03 (7870.78,11712.52) | | 1440.27 (1001.65,1989.88) | |
| South Sudan | 6371.55 (5182.42,7849.33) | 6588.1 (5207.57,8708.03) | | 1064.99 (683.26,1529.08) | | 6902.35 (5352.94,8928.99) | 7372.22 (5276.35,10352.22) | | 1177.08 (731.71,1732.12) | |
| Spain | 7140.36 (6464.03,8019.61) | 8147.4 (7235.79,9197.3) | | 1288.32 (898.84,1743.16) | | 10132.18 (8026.06,12693.62) | 12546.99 (9234.03,16712.24) | | 1895.68 (1259.37,2749.84) | |
| Sri Lanka | 5019.44 (4233.8,5926.38) | 4989.53 (4102.23,6064.42) | | 844.49 (579.74,1179.43) | | 4912.24 (3983.02,6110) | 4614.58 (3468.15,6209.29) | | 804.86 (541.15,1156.35) | |
| Sudan | 6924.55 (5642.73,8849.96) | 8137.1 (6452.42,10872.56) | | 1243.64 (812.2,1833.45) | | 7522.99 (5741.97,9882.61) | 8987.85 (6333.86,12715.94) | | 1361.6 (839.33,2012.27) | |
| Suriname | 6762.46 (5604.94,8350.49) | 8435.87 (6651.31,10689.16) | | 1260.69 (848.3,1770.85) | | 9662.8 (7199.21,12606.06) | 12620.32 (9219.42,17140.33) | | 1842.36 (1141.63,2758.67) | |
| Sweden | 8439.42 (7357.61,9632.62) | 10062.91 (8469.79,11852) | | 1551.58 (1066.59,2112.55) | | 9812.54 (7950.46,11969.58) | 12026.22 (9416.1,15136.56) | | 1829.79 (1225.48,2586.67) | |
| Switzerland | 7968.71 (6736.43,9343.81) | 9329.44 (7628.79,11309.78) | | 1447.59 (985.56,2014.78) | | 7971.45 (6065.91,10327.59) | 9377.08 (6932.27,12751.13) | | 1443.01 (926.32,2086.69) | |
| Syrian Arab Republic | 6355.77 (5128.68,8096.15) | 7346.59 (5688.68,9778.98) | | 1135.28 (721.17,1683.48) | | 7611.24 (5656.35,10171.27) | 9081.55 (6463.07,13098.15) | | 1375.33 (856.39,2120.63) | |
| Taiwan (Province of China) | 3272.24 (2668.23,4020.32) | 2467.89 (2020.19,3141.64) | | 483.74 (325.86,683.72) | | 4110.92 (3256.09,5077.43) | 3206.42 (2396.03,4268.79) | | 617.48 (383.12,882.58) | |
| Tajikistan | 4124.17 (3381.38,5081.16) | 4177.63 (3290.4,5380.17) | | 691.86 (456.65,973.58) | | 4912.75 (3844.35,6266.15) | 5186.74 (3757.77,7142.47) | | 841.26 (533.48,1258.67) | |
| Thailand | 4035.55 (3335.4,4944.28) | 3563.78 (2905.38,4578.38) | | 640.41 (426.58,901.78) | | 4591.17 (3592.82,5774.41) | 3964.16 (2881.7,5449.55) | | 721.38 (458.35,1054.68) | |
| Timor-Leste | 4276.91 (3566.04,5283.94) | 3973.87 (3172.74,5139.22) | | 693.6 (458.82,971.23) | | 4182.79 (3318.67,5489.29) | 3958.35 (2836.82,5428.3) | | 688.54 (424.7,1047.57) | |
| Togo | 5918.08 (4877.34,7302.85) | 6257.17 (4985.04,8081.81) | | 1002.87 (659.59,1418.53) | | 6402.49 (4919.55,8135.02) | 6860.81 (4734.44,9316.26) | | 1095.44 (688.42,1642.67) | |
| Tokelau | 4270.76 (3488.07,5298.79) | 3976.16 (3000.32,5281.41) | | 690.37 (451.37,1006.34) | | 4810.87 (3787.1,6261) | 4600.64 (3194.74,6475.44) | | 789.07 (488.72,1196.97) | |
| Tonga | 3730.81 (3098.47,4546.91) | 3304.46 (2648.23,4251.43) | | 590.62 (390.17,835.17) | | 4033.61 (3258.02,5159.19) | 3572.1 (2586.05,4969.75) | | 636.75 (423.3,931.69) | |
| Trinidad and Tobago | 6098.05 (4980.69,7296.68) | 7411.12 (5888.47,9134.38) | | 1121.49 (728.63,1564) | | 7655.8 (5597.61,10073.32) | 9623.63 (6770.56,13133.9) | | 1426.67 (855,2229.98) | |
| Tunisia | 7691.54 (6235.92,9716.06) | 9234.79 (7315.66,12134.04) | | 1410.76 (893.7,2045.53) | | 10281.82 (7708.41,13562.23) | 12844.18 (9080.25,17916.98) | | 1907.65 (1167.28,2891.84) | |
| Türkiye | 6799.41 (5981.94,7701.57) | 8032.18 (7002.55,9248.03) | | 1233.13 (861.76,1672.49) | | 8194.53 (6215.54,10706.47) | 9904.48 (7025.24,13992.91) | | 1499.73 (901.55,2341.58) | |
| Turkmenistan | 4382.49 (3615.01,5302.55) | 4506.46 (3594.9,5652.62) | | 740.57 (487.2,1043.23) | | 5054.35 (3954.14,6341.62) | 5367.72 (3880.72,7255.84) | | 867.29 (546.01,1270.68) | |
| Tuvalu | 4368.22 (3572.83,5456.97) | 3970.1 (2999.14,5264.59) | | 700.19 (453.1,1022.61) | | 4692.62 (3653.85,6130.71) | 4562.97 (3280.72,6532.95) | | 777.25 (487.82,1183) | |
| Uganda | 8610.99 (7070.68,10806.45) | 9951.85 (7834.35,13026.4) | | 1528.77 (992.81,2171.4) | | 9413.75 (7279.17,12236.19) | 11155.12 (8077.48,15504.4) | | 1710.17 (1067.17,2574.11) | |
| Ukraine | 6159.94 (5217.97,7365.46) | 6990.55 (5657.73,8671.07) | | 1085.32 (721.88,1518.93) | | 7565.11 (5907.62,9550.26) | 8926.63 (6547.5,11980.21) | | 1358.43 (873.21,1958.54) | |
| United Arab Emirates | 6675.26 (5442.02,8387.61) | 7693.62 (6036.48,10280.12) | | 1186.12 (768.88,1696.62) | | 7804.7 (5951,10214.07) | 9049.04 (6365.27,12817.72) | | 1383.29 (835.03,2096.35) | |
| United Kingdom | 7838.76 (6731.82,9283.74) | 9354.17 (7670.8,11532.37) | | 1430.93 (944.22,1993.94) | | 8533.92 (7222.06,10182.46) | 10382.27 (8448.54,12826.77) | | 1568.2 (1042.97,2209.15) | |
| United Republic of Tanzania | 6361.91 (5192.17,7746.56) | 6578.87 (5165.49,8578.34) | | 1049.57 (687.54,1492.95) | | 6915.19 (5432.39,8939.85) | 7356.74 (5242.65,10305.8) | | 1176.45 (726.71,1717.19) | |
| United States of America | 7131.37 (6245.93,8090.46) | 7159.65 (6117.93,8549.02) | | 1205.76 (837.14,1652.9) | | 10619.56 (9311.22,12094.68) | 12866.86 (10994.4,15213.7) | | 1955.18 (1373.76,2651.74) | |
| United States Virgin Islands | 4804.13 (3879.98,5966.73) | 5445.34 (4162.99,7060.64) | | 849.41 (544.63,1225.42) | | 5891.83 (4564.88,7547.92) | 7016.51 (5086.69,9621.91) | | 1065.85 (643.9,1543.52) | |
| Uruguay | 4988.11 (4128.9,6198.54) | 5921.23 (4723.53,7722.61) | | 910.83 (604.19,1291.22) | | 7043.52 (5336.18,9087.43) | 8923.64 (6546.32,11982.93) | | 1329.51 (827.44,1963.5) | |
| Uzbekistan | 4471.42 (3625.31,5404.45) | 4659.51 (3695.3,6012.71) | | 758.29 (507.72,1073.02) | | 5110 (4006.34,6540.44) | 5368.47 (3906.55,7300.89) | | 870.69 (554.33,1281.09) | |
| Vanuatu | 4176.23 (3445.68,5084.28) | 3933.85 (3069.75,5127.18) | | 681.16 (447.48,969.58) | | 4401.06 (3489.35,5478.22) | 4140.87 (3034.51,5643.61) | | 713.44 (454.86,1033.58) | |
| Venezuela (Bolivarian Republic of) | 4432.8 (3627.72,5629.35) | 5195.47 (4028.56,6893.31) | | 796.68 (506.19,1156.85) | | 5190.12 (3918.85,6792.59) | 6188.16 (4440.33,8516.19) | | 940.32 (569.68,1416.75) | |
| Viet Nam | 3742.93 (3105.48,4526.49) | 3298.75 (2661.79,4101.85) | | 594.48 (397.6,840.12) | | 4253.95 (3431.45,5261.76) | 3585 (2644.04,4817.47) | | 663.52 (428.95,961.51) | |
| Yemen | 8189.59 (6600.03,10425.17) | 10013.87 (7853.54,13005.74) | | 1483.52 (959.54,2146.77) | | 8192.84 (6232.74,10674.89) | 9976.34 (7195.85,14187.64) | | 1482.68 (911.05,2171.15) | |
| Zambia | 5486.86 (4505.88,6776.42) | 5318.22 (4150.24,7123.35) | | 884.87 (574.69,1256.81) | | 6259.83 (4914.45,8021.56) | 6394.45 (4676.9,9175.74) | | 1038.45 (671.69,1547.71) | |
| Zimbabwe | 4291.72 (3510.31,5333.32) | 3880.72 (3038.75,5106.4) | | 670.35 (445.78,949.11) | | 5106.52 (4040.66,6487.13) | 4993.11 (3567.33,6750.9) | | 828.22 (536.12,1199.44) | |

**Table S2**. Changes in prevalence, incidence, and disability-adjusted life years of depression in women of child-bearing age in 204 countries from 1990 to 2021.

|  | Prevalence | | | Incidence | | | Disability-adjusted life years | | |
| --- | --- | --- | --- | --- | --- | --- | --- | --- | --- |
| location | number_1990 | number_2021 | Percentage change/% | number_1990 | number_2021 | Percentage change/% | number_1990 | number_2021 | Percentage change/% |
| Afghanistan | 168977.6412 | 650095.35 | 284.7227 | 205540.2425 | 816358.0487 | 297.1767 | 30636.4832 | 120269.6257 | 292.5699 |
| Albania | 31762.4184 | 31187.6859 | -1.8095 | 29921.3805 | 32574.4176 | 8.8667 | 5150.8728 | 5319.5316 | 3.2744 |
| Algeria | 386858.862 | 860104.484 | 122.3303 | 452078.4625 | 1009295.674 | 123.2567 | 69752.7704 | 154767.5532 | 121.8802 |
| American Samoa | 427.7109 | 468.8114 | 9.6094 | 358.8449 | 398.1627 | 10.9567 | 66.0717 | 72.1283 | 9.1667 |
| Andorra | 986.1337 | 1566.4702 | 58.8497 | 1125.2494 | 1863.3878 | 65.5978 | 177.0957 | 285.7538 | 61.3555 |
| Angola | 207226.1537 | 750600.1049 | 262.213 | 251570.1672 | 930141.4019 | 269.7344 | 37705.8385 | 138349.0449 | 266.9168 |
| Antigua and Barbuda | 735.8158 | 1390.8225 | 89.0178 | 831.9627 | 1652.6942 | 98.65 | 129.7345 | 251.2345 | 93.6528 |
| Argentina | 375201.8564 | 656161.9283 | 74.8824 | 439552.5976 | 792741.364 | 80.3519 | 67901.0522 | 120724.9434 | 77.7954 |
| Armenia | 34405.613 | 38839.6791 | 12.8876 | 32519.3257 | 40675.0274 | 25.0796 | 5595.8452 | 6630.8049 | 18.4951 |
| Australia | 347654.9794 | 519833.1867 | 49.5256 | 412157.275 | 626911.1439 | 52.1048 | 63364.383 | 95816.3086 | 51.2148 |
| Austria | 125498.0091 | 126193.0633 | 0.5538 | 142071.3661 | 142777.3828 | 0.4969 | 22345.4947 | 22376.0423 | 0.1367 |
| Azerbaijan | 72644.7567 | 138361.5621 | 90.4632 | 69319.3147 | 143451.945 | 106.9437 | 11855.0006 | 23482.1021 | 98.0776 |
| Bahamas | 3281.0692 | 6316.6616 | 92.5184 | 3724.5887 | 7603.9222 | 104.1547 | 579.1431 | 1147.1825 | 98.0827 |
| Bahrain | 9523.1642 | 29291.3857 | 207.5804 | 11524.8195 | 35755.127 | 210.2446 | 1751.7495 | 5376.0589 | 206.8966 |
| Bangladesh | 1779731.335 | 3801694.781 | 113.6106 | 2156227.991 | 4678225.754 | 116.9634 | 323985.1953 | 697249.1977 | 115.2102 |
| Barbados | 3136.2022 | 4303.9374 | 37.2341 | 3531.1769 | 5170.3257 | 46.4193 | 552.9362 | 781.4063 | 41.3194 |
| Belarus | 142909.0319 | 174829.2953 | 22.3361 | 156788.729 | 210932.4393 | 34.5329 | 24944.8312 | 31918.8617 | 27.9578 |
| Belgium | 138855.3235 | 183585.0743 | 32.2132 | 157630.5543 | 223080.1114 | 41.5209 | 24700.4993 | 33687.4588 | 36.3837 |
| Belize | 1860.9971 | 6852.0461 | 268.1922 | 2145.8075 | 8276.6344 | 285.7119 | 331.3524 | 1248.2062 | 276.7006 |
| Benin | 64513.2373 | 204198.3043 | 216.5216 | 67630.3716 | 221364.282 | 227.3149 | 10838.721 | 35107.8898 | 223.9117 |
| Bermuda | 944.2719 | 814.599 | -13.7326 | 1097.8424 | 968.267 | -11.8027 | 170.6344 | 148.0558 | -13.2321 |
| Bhutan | 7721.9484 | 11585.6371 | 50.0352 | 8668.7182 | 12702.3983 | 46.5314 | 1341.729 | 1998.9984 | 48.9868 |
| Bolivia (Plurinational State of) | 82361.5556 | 219700.699 | 166.7515 | 98632.7319 | 276563.5097 | 180.3973 | 14968.703 | 40938.9167 | 173.4968 |
| Bosnia and Herzegovina | 60176.8284 | 41039.6955 | -31.8015 | 63751.1568 | 44036.4811 | -30.9244 | 10406.2396 | 7072.1209 | -32.0396 |
| Botswana | 18382.9933 | 52180.3234 | 183.8511 | 19259.2841 | 59035.6266 | 206.5307 | 3108.1099 | 9075.3374 | 191.9889 |
| Brazil | 2539487.326 | 4589446.411 | 80.7233 | 3184584.883 | 5832145.999 | 83.1368 | 468480.0969 | 851092.256 | 81.671 |
| Brunei Darussalam | 1896.4472 | 3948.8468 | 108.2234 | 1938.5182 | 4190.5763 | 116.1742 | 323.5253 | 685.5191 | 111.8904 |
| Bulgaria | 95901.6304 | 82528.6659 | -13.9445 | 95487.43 | 89168.2602 | -6.6178 | 15965.7919 | 14225.8212 | -10.8981 |
| Burkina Faso | 120086.6816 | 318506.0938 | 165.2302 | 124804.3215 | 333696.4223 | 167.3757 | 20150.7484 | 53989.7711 | 167.9294 |
| Burundi | 92414.1229 | 222612.5763 | 140.8859 | 100404.6961 | 240532.1465 | 139.5626 | 15948.618 | 38412.9698 | 140.8545 |
| Cabo Verde | 4444.3348 | 11249.4912 | 153.1198 | 4618.9416 | 12676.4097 | 174.444 | 753.8965 | 1988.8592 | 163.8106 |
| Cambodia | 113722.8924 | 212653.5963 | 86.9928 | 110681.8224 | 201738.2545 | 82.2686 | 18753.9272 | 34858.5019 | 85.8731 |
| Cameroon | 144374.0789 | 512696.1658 | 255.1165 | 154104.9089 | 561765.5794 | 264.5345 | 24532.7031 | 88669.0249 | 261.4319 |
| Canada | 459017.9512 | 653781.674 | 42.4305 | 475938.8936 | 734711.1148 | 54.3709 | 78534.5927 | 116418.1428 | 48.238 |
| Central African Republic | 57569.6045 | 130048.2194 | 125.8974 | 69771.4288 | 159401.8258 | 128.4629 | 10381.8848 | 23725.7596 | 128.5304 |
| Chad | 91621.3031 | 272088.3733 | 196.9706 | 102280.9696 | 309154.2937 | 202.2598 | 15994.8613 | 48029.4309 | 200.2804 |
| Chile | 298421.912 | 413410.851 | 38.5323 | 384725.8923 | 534248.4136 | 38.8647 | 57348.2404 | 79171.6634 | 38.0542 |
| China | 14618427.48 | 12975935.93 | -11.2358 | 14240887.95 | 9996508.015 | -29.8042 | 2428798.621 | 1934532.654 | -20.3502 |
| Colombia | 285919.9394 | 424888.7865 | 48.6041 | 311170.6426 | 447994.1146 | 43.9706 | 49359.864 | 72146.5665 | 46.1644 |
| Comoros | 6101.3561 | 12886.8469 | 111.2128 | 6012.6778 | 13246.9277 | 120.3166 | 1004.3013 | 2171.4684 | 116.2168 |
| Congo | 49610.7429 | 139328.225 | 180.8429 | 60329.54 | 170621.577 | 182.816 | 9002.2432 | 25459.6457 | 182.8145 |
| Cook Islands | 195.8611 | 209.2103 | 6.8157 | 183.8958 | 199.4592 | 8.4632 | 31.9035 | 34.2663 | 7.406 |
| Costa Rica | 34441.4596 | 77015.4608 | 123.6127 | 40204.1045 | 94764.4392 | 135.7084 | 6211.6866 | 14247.0223 | 129.3584 |
| Cite d'Ivoire | 144681.3922 | 371739.9068 | 156.9369 | 145557.0226 | 377793.1628 | 159.5499 | 23658.1075 | 61786.3953 | 161.1637 |
| Croatia | 62537.64 | 47752.8877 | -23.6414 | 65664.6638 | 50177.2276 | -23.5856 | 10736.6501 | 8150.7251 | -24.085 |
| Cuba | 246679.4452 | 167299.5677 | -32.1794 | 315640.3702 | 205902.3308 | -34.7668 | 46862.9598 | 30798.0674 | -34.2806 |
| Cyprus | 11195.6808 | 23449.8944 | 109.4548 | 12210.2512 | 26507.5027 | 117.0922 | 1961.8357 | 4174.3598 | 112.7783 |
| Czechia | 132450.4489 | 128658.4217 | -2.863 | 138722.7174 | 136619.9656 | -1.5158 | 22578.1431 | 22037.6576 | -2.3938 |
| Democratic People's Republic of Korea | 223316.5337 | 264156.3257 | 18.2878 | 197025.7367 | 218815.6984 | 11.0594 | 35444.9884 | 40627.497 | 14.6213 |
| Democratic Republic of the Congo | 720613.792 | 1849042.304 | 156.5927 | 862485.532 | 2226408.03 | 158.1386 | 128255.5074 | 333559.5025 | 160.0742 |
| Denmark | 105813.7801 | 92010.7453 | -13.0446 | 127726.3539 | 108627.2466 | -14.9531 | 19489.1047 | 16663.7401 | -14.4971 |
| Djibouti | 5899.3194 | 22626.1146 | 283.5377 | 5915.7994 | 23557.405 | 298.2117 | 983.3292 | 3844.0816 | 290.9252 |
| Dominica | 754.0027 | 941.5973 | 24.8798 | 860.6957 | 1132.2446 | 31.5499 | 133.1991 | 170.8 | 28.2291 |
| Dominican Republic | 104025.9214 | 192312.0878 | 84.8694 | 125950.9539 | 238392.8644 | 89.2744 | 19088.0164 | 35652.2635 | 86.7783 |
| Ecuador | 124089.8309 | 300052.8047 | 141.8029 | 144465.5998 | 366019.074 | 153.3607 | 22431.4 | 55490.2119 | 147.3774 |
| Egypt | 816658.4268 | 1949841.325 | 138.7585 | 924351.5161 | 2310619.187 | 149.9719 | 144321.4542 | 353478.2839 | 144.9243 |
| El Salvador | 79821.9937 | 114766.6703 | 43.7783 | 100394.6854 | 143678.9432 | 43.1141 | 15008.1514 | 21551.0975 | 43.5959 |
| Equatorial Guinea | 8971.3645 | 35021.9069 | 290.3744 | 10906.6529 | 43025.0362 | 294.4843 | 1619.2767 | 6411.627 | 295.9562 |
| Eritrea | 52036.4063 | 116599.2086 | 124.0724 | 54262.6929 | 123943.0425 | 128.413 | 8760.0843 | 20021.3966 | 128.5526 |
| Estonia | 26704.1784 | 19343.2327 | -27.5648 | 31090.9617 | 22408.2151 | -27.9269 | 4821.6121 | 3458.5074 | -28.2707 |
| Eswatini | 11054.5946 | 26893.7506 | 143.2812 | 11556.4204 | 31936.6301 | 176.354 | 1876.5234 | 4791.4771 | 155.338 |
| Ethiopia | 728808.5195 | 1787211.718 | 145.2238 | 737811.4875 | 1863646.173 | 152.5911 | 121019.0588 | 302261.4778 | 149.7635 |
| Fiji | 7810.0275 | 10581.7763 | 35.4896 | 6944.5022 | 9891.8449 | 42.4414 | 1236.1689 | 1714.5764 | 38.7008 |
| Finland | 111548.0223 | 104146.6141 | -6.6352 | 132134.5898 | 125246.9107 | -5.2126 | 20506.9022 | 19215.4164 | -6.2978 |
| France | 1151966.435 | 1192020.614 | 3.477 | 1379766.989 | 1453396.11 | 5.3363 | 211807.5046 | 219866.6877 | 3.805 |
| Gabon | 18107.0312 | 45038.0744 | 148.7325 | 21559.7325 | 54696.5438 | 153.6977 | 3248.8737 | 8176.3703 | 151.6678 |
| Gambia | 20300.9559 | 58240.8497 | 186.8872 | 24439.772 | 71057.6326 | 190.7459 | 3708.1248 | 10690.6793 | 188.3042 |
| Georgia | 59941.0784 | 43984.192 | -26.621 | 59067.7204 | 47299.1196 | -19.9239 | 9961.8505 | 7585.377 | -23.8557 |
| Germany | 1019809.097 | 1192125.828 | 16.897 | 1104427.572 | 1422779.13 | 28.825 | 176485.7527 | 216611.3313 | 22.7359 |
| Ghana | 208165.4291 | 591396.8569 | 184.0995 | 218564.6225 | 636422.8379 | 191.1829 | 35147.3522 | 101594.759 | 189.0538 |
| Greece | 219757.3827 | 235558.0778 | 7.1901 | 268542.2653 | 298085.7679 | 11.0014 | 41025.5222 | 44583.5576 | 8.6727 |
| Greenland | 2109.7448 | 1990.4438 | -5.6548 | 2665.8377 | 2563.9255 | -3.8229 | 403.7341 | 383.6638 | -4.9712 |
| Grenada | 876.6915 | 1471.5518 | 67.8529 | 1005.0129 | 1764.3161 | 75.5516 | 155.3522 | 267.0084 | 71.873 |
| Guam | 1436.6333 | 1738.8673 | 21.0377 | 1280.5493 | 1637.9899 | 27.9131 | 229.7954 | 284.581 | 23.841 |
| Guatemala | 96001.9572 | 289718.6101 | 201.7841 | 118097.4397 | 369084.792 | 212.5257 | 17493.62 | 54107.2 | 209.2968 |
| Guinea | 83562.2821 | 219123.2601 | 162.2275 | 88185.2543 | 241093.5626 | 173.3944 | 14143.3476 | 38017.0527 | 168.7981 |
| Guinea-Bissau | 14223.6678 | 35512.9794 | 149.6753 | 15227.5011 | 39289.1997 | 158.0148 | 2423.815 | 6160.4403 | 154.163 |
| Guyana | 13947.4815 | 20281.2559 | 45.4116 | 17558.3719 | 26717.2531 | 52.1625 | 2603.1943 | 3870.0338 | 48.6648 |
| Haiti | 86693.9099 | 230962.6152 | 166.4116 | 104959.4354 | 285992.5559 | 172.4791 | 15635.8612 | 42183.6566 | 169.7879 |
| Honduras | 44162.5186 | 161817.3535 | 266.4133 | 51598.2178 | 201613.6224 | 290.7376 | 7904.2048 | 29902.3547 | 278.3095 |
| Hungary | 136170.2171 | 111479.9272 | -18.1319 | 144737.4923 | 115532.6651 | -20.1778 | 23350.7483 | 18837.0906 | -19.3298 |
| Iceland | 3829.8859 | 4610.0121 | 20.3694 | 4252.6976 | 5055.6494 | 18.881 | 676.9162 | 807.7273 | 19.3246 |
| India | 11776149.59 | 22836700.25 | 93.9233 | 13368733.12 | 25586191.12 | 91.3883 | 2041894.341 | 3959612.738 | 93.9186 |
| Indonesia | 1624374.013 | 3189280.69 | 96.3391 | 1304095.292 | 2752422.312 | 111.0599 | 245895.3968 | 500178.6237 | 103.4111 |
| Iran (Islamic Republic of) | 1060506.149 | 2353351.619 | 121.9083 | 1293092.598 | 2868731.247 | 121.8504 | 194454.785 | 430877.3112 | 121.5823 |
| Iraq | 262737.019 | 755613.0213 | 187.5929 | 305180.7438 | 888934.8575 | 191.2814 | 46848.8044 | 134953.0756 | 188.0609 |
| Ireland | 60033.8822 | 106407.1917 | 77.2452 | 70917.8484 | 132688.6353 | 87.1019 | 10918.6351 | 19869.7321 | 81.98 |
| Israel | 90675.5103 | 184413.4079 | 103.3773 | 105998.4926 | 220953.6106 | 108.4498 | 16543.0924 | 34037.1872 | 105.7486 |
| Italy | 949550.6437 | 989535.245 | 4.2109 | 1098546.505 | 1193614.611 | 8.654 | 170632.2698 | 182075.577 | 6.7064 |
| Jamaica | 26515.424 | 45314.1042 | 70.8972 | 30345.2233 | 54656.5966 | 80.116 | 4708.767 | 8268.1512 | 75.5906 |
| Japan | 1104898.015 | 1088945.4 | -1.4438 | 1206511.014 | 1264770.291 | 4.8287 | 194255.8034 | 198474.6111 | 2.1718 |
| Jordan | 61056.0948 | 244693.4733 | 300.7683 | 73567.3263 | 294119.0943 | 299.7958 | 11180.2987 | 44559.9767 | 298.558 |
| Kazakhstan | 192890.391 | 270336.9219 | 40.1505 | 198448.9288 | 293860.3981 | 48.0786 | 32676.0125 | 47115.6373 | 44.1903 |
| Kenya | 328833.3725 | 893841.3018 | 171.822 | 346462.8255 | 947907.0237 | 173.5956 | 55830.0535 | 152287.865 | 172.7704 |
| Kiribati | 766.7823 | 1332.9562 | 73.8376 | 701.0482 | 1193.7206 | 70.2766 | 122.4252 | 210.4279 | 71.8828 |
| Kuwait | 28458.7102 | 106042.2904 | 272.618 | 32930.0549 | 120171.3487 | 264.9291 | 5114.4283 | 18728.0737 | 266.1812 |
| Kyrgyzstan | 49307.3566 | 98447.407 | 99.6607 | 51998.5378 | 109173.0215 | 109.954 | 8454.7578 | 17331.4197 | 104.9901 |
| Lao People's Democratic Republic | 37253.9482 | 77281.5691 | 107.4453 | 33219.2777 | 65574.7083 | 97.3996 | 5892.882 | 12060.6558 | 104.6648 |
| Latvia | 40142.3225 | 28129.5109 | -29.9256 | 45193.6344 | 32992.1017 | -26.9983 | 7094.0084 | 5052.6812 | -28.7754 |
| Lebanon | 51111.9657 | 154539.2539 | 202.3544 | 59090.8898 | 194117.675 | 228.5069 | 9092.1417 | 28651.7472 | 215.1265 |
| Lesotho | 32491.4831 | 54736.2525 | 68.4634 | 38287.0074 | 68353.9099 | 78.5303 | 5887.0671 | 10022.1763 | 70.2406 |
| Liberia | 39961.2849 | 105930.3291 | 165.0824 | 45201.7534 | 121343.8126 | 168.4493 | 6873.5422 | 18439.3335 | 168.2654 |
| Libya | 61506.6376 | 160740.6839 | 161.3388 | 72683.7208 | 191420.1004 | 163.3603 | 11162.6405 | 28985.6739 | 159.6668 |
| Lithuania | 57728.0545 | 46928.7955 | -18.7071 | 65502.7922 | 56861.0365 | -13.193 | 10262.3342 | 8591.1931 | -16.2842 |
| Luxembourg | 6784.7919 | 10197.87 | 50.3048 | 7841.883 | 11586.38 | 47.75 | 1223.9439 | 1814.9044 | 48.2833 |
| Madagascar | 171954.8108 | 508942.8225 | 195.9748 | 177312.0295 | 545312.8987 | 207.5442 | 28842.0991 | 87433.8899 | 203.1468 |
| Malawi | 132862.9829 | 314387.3215 | 136.6252 | 131682.6244 | 323814.0794 | 145.9049 | 21504.8742 | 52635.0977 | 144.7589 |
| Malaysia | 186339.1398 | 413104.9821 | 121.6952 | 170181.7234 | 397970.0536 | 133.8501 | 30046.1265 | 68434.2876 | 127.7641 |
| Maldives | 2573.8821 | 5869.517 | 128.0414 | 2790.655 | 5590.7579 | 100.3386 | 449.6026 | 960.8812 | 113.7179 |
| Mali | 99703.8748 | 282106.6184 | 182.9445 | 98492.2297 | 283031.9871 | 187.3648 | 16242.6767 | 46497.2343 | 186.2658 |
| Malta | 5384.694 | 6093.5091 | 13.1635 | 5856.0577 | 6874.5038 | 17.3913 | 940.5338 | 1081.2613 | 14.9625 |
| Marshall Islands | 374.6974 | 628.1228 | 67.6347 | 342.5662 | 564.956 | 64.9188 | 60.0484 | 99.3885 | 65.514 |
| Mauritania | 22917.9626 | 54653.2134 | 138.4733 | 21999.9699 | 53751.0861 | 144.3235 | 3703.529 | 8994.0684 | 142.8513 |
| Mauritius | 20253.406 | 23085.3254 | 13.9824 | 22295.9765 | 25345.6306 | 13.678 | 3620.6559 | 4106.6109 | 13.4217 |
| Mexico | 951302.2074 | 2723620.476 | 186.3044 | 1101545.313 | 3509491.706 | 218.5971 | 169310.5613 | 513075.2148 | 203.0379 |
| Micronesia (Federated States of) | 911.208 | 1088.26 | 19.4305 | 841.349 | 986.8204 | 17.2902 | 146.5969 | 173.8612 | 18.5981 |
| Monaco | 506.5503 | 630.9458 | 24.5574 | 589.0504 | 773.3531 | 31.2881 | 91.7387 | 116.9076 | 27.4354 |
| Mongolia | 28740.9159 | 50683.1579 | 76.345 | 32722.8708 | 56123.7546 | 71.5123 | 5127.8678 | 8927.9044 | 74.1056 |
| Montenegro | 6958.5575 | 8287.4485 | 19.0972 | 6933.583 | 9050.8498 | 30.5364 | 1163.5901 | 1439.2561 | 23.691 |
| Morocco | 502449.3273 | 916597.9377 | 82.4259 | 610012.5874 | 1136465.912 | 86.302 | 92224.2411 | 169165.6587 | 83.4286 |
| Mozambique | 201122.2105 | 552989.4672 | 174.952 | 205803.6532 | 605865.5006 | 194.3901 | 33183.3033 | 93502.9276 | 181.777 |
| Myanmar | 295868.84 | 535791.5997 | 81.0909 | 200268.4525 | 411429.8588 | 105.4392 | 41353.5828 | 79427.2478 | 92.0686 |
| Namibia | 17007.3096 | 43788.6844 | 157.4698 | 16733.5139 | 47764.2388 | 185.4406 | 2784.3319 | 7538.607 | 170.751 |
| Nauru | 103.3498 | 134.1064 | 29.7598 | 96.4229 | 131.3219 | 36.1936 | 16.7262 | 22.1992 | 32.721 |
| Nepal | 270990.1438 | 740819.3617 | 173.375 | 308758.1663 | 915446.1997 | 196.493 | 47146.6342 | 135810.5688 | 188.0599 |
| Netherlands | 260277.7384 | 279299.9526 | 7.3084 | 303642.8398 | 337063.423 | 11.0065 | 47177.8397 | 51452.7477 | 9.0613 |
| New Zealand | 60171.196 | 84432.0098 | 40.3196 | 67844.5958 | 96248.6947 | 41.8664 | 10578.1728 | 15085.4387 | 42.6091 |
| Nicaragua | 43253.8843 | 108542.3934 | 150.9425 | 52121.1863 | 134359.3715 | 157.7826 | 7903.7638 | 20160.82 | 155.0787 |
| Niger | 102166.0738 | 301918.0266 | 195.5169 | 107831.957 | 319052.7029 | 195.8795 | 17292.0688 | 51469.6047 | 197.6486 |
| Nigeria | 1111836.632 | 2881907.831 | 159.2025 | 1161085.384 | 2837020.385 | 144.3421 | 185470.802 | 470963.3279 | 153.9286 |
| Niue | 21.503 | 18.6664 | -13.1914 | 19.7024 | 17.657 | -10.3817 | 3.4647 | 3.0381 | -12.3118 |
| North Macedonia | 21454.5646 | 28811.8666 | 34.2925 | 20745.5502 | 30479.9204 | 46.9227 | 3513.7245 | 4934.3059 | 40.4295 |
| Northern Mariana Islands | 484.367 | 501.1089 | 3.4565 | 389.9596 | 432.3312 | 10.8656 | 73.5129 | 78.4035 | 6.6527 |
| Norway | 67046.1364 | 92638.1976 | 38.1708 | 74997.9551 | 108814.6042 | 45.0901 | 11914.8349 | 16886.6977 | 41.7283 |
| Oman | 22041.9276 | 80509.4251 | 265.2558 | 25354.6019 | 95445.3516 | 276.4419 | 3930.4105 | 14559.7048 | 270.4373 |
| Pakistan | 1333356.493 | 3807809.362 | 185.5807 | 1504175.926 | 4383428.698 | 191.4173 | 231455.4681 | 668286.6353 | 188.7323 |
| Palau | 176.3912 | 187.5264 | 6.3128 | 163.3656 | 172.7037 | 5.716 | 28.5089 | 30.1101 | 5.6166 |
| Palestine | 38202.2888 | 131090.7488 | 243.1489 | 47428.866 | 165980.1931 | 249.9561 | 7120.7938 | 24665.3103 | 246.3843 |
| Panama | 25810.7029 | 57786.5204 | 123.8859 | 29844.8669 | 70233.7276 | 135.3293 | 4617.6105 | 10593.3299 | 129.4115 |
| Papua New Guinea | 42029.896 | 116035.2812 | 176.078 | 40460.1379 | 108320.9825 | 167.7227 | 6874.6082 | 18752.6754 | 172.7817 |
| Paraguay | 49883.7878 | 137309.5102 | 175.2588 | 58987.4777 | 172090.7996 | 191.7412 | 8997.8991 | 25509.1519 | 183.5012 |
| Peru | 188440.0872 | 419426.9117 | 122.5784 | 195569.1516 | 462754.8816 | 136.6196 | 31724.9464 | 72861.0292 | 129.6648 |
| Philippines | 627336.0809 | 1362928.08 | 117.2564 | 583327.6681 | 1310788.451 | 124.7088 | 100985.8033 | 223734.4897 | 121.5504 |
| Poland | 318479.6565 | 354240.4213 | 11.2286 | 258564.2768 | 311864.4184 | 20.6139 | 47858.4386 | 55420.4206 | 15.8007 |
| Portugal | 233564.8358 | 241642.6556 | 3.4585 | 288591.7008 | 304201.8163 | 5.4091 | 43768.8023 | 45479.9262 | 3.9095 |
| Puerto Rico | 37644.0419 | 35250.675 | -6.3579 | 40049.7482 | 39468.4743 | -1.4514 | 6431.8039 | 6170.4114 | -4.0641 |
| Qatar | 6060.4125 | 44503.963 | 634.3388 | 7168.4515 | 52234.7173 | 628.675 | 1096.6378 | 7999.7579 | 629.4804 |
| Republic of Korea | 441218.0302 | 462215.2707 | 4.7589 | 472253.9823 | 507502.2513 | 7.4638 | 76944.4798 | 81841.7639 | 6.3647 |
| Republic of Moldova | 60046.8827 | 53003.189 | -11.7303 | 64623.4633 | 58333.7631 | -9.7328 | 10360.1936 | 9213.2893 | -11.0703 |
| Romania | 232100.6504 | 212221.6353 | -8.5648 | 222271.8106 | 221116.1439 | -0.5199 | 37744.3153 | 35929.8191 | -4.8073 |
| Russian Federation | 1749450.178 | 2031948.891 | 16.1479 | 1774636.553 | 2212681.993 | 24.6837 | 291520.1612 | 350940.289 | 20.3829 |
| Rwanda | 128438.0928 | 286237.5108 | 122.8603 | 143936.0763 | 321520.2019 | 123.3771 | 22627.9583 | 50553.3443 | 123.411 |
| Saint Kitts and Nevis | 549.5841 | 1106.6756 | 101.366 | 665.5591 | 1378.592 | 107.1329 | 100.2583 | 205.6446 | 105.1148 |
| Saint Lucia | 1535.1032 | 2863.8917 | 86.5602 | 1765.0755 | 3472.438 | 96.7303 | 271.8665 | 521.4388 | 91.7996 |
| Saint Vincent and the Grenadines | 1169.9199 | 1646.7399 | 40.7566 | 1345.6645 | 1982.5985 | 47.3323 | 207.9529 | 298.7532 | 43.6639 |
| Samoa | 1399.1662 | 1970.323 | 40.8212 | 1274.0174 | 1775.2889 | 39.3457 | 224.523 | 314.1249 | 39.9077 |
| San Marino | 445.5127 | 637.2546 | 43.0385 | 522.9098 | 783.5083 | 49.8362 | 81.1779 | 118.4585 | 45.9244 |
| Sao Tome and Principe | 1362.5525 | 3289.3259 | 141.4091 | 1383.8837 | 3418.7664 | 147.0415 | 228.5506 | 558.6443 | 144.4292 |
| Saudi Arabia | 212126.6278 | 792606.4706 | 273.6478 | 244937.3915 | 930535.7064 | 279.9076 | 37891.4836 | 142573.866 | 276.2689 |
| Senegal | 88033.5045 | 232408.0549 | 163.9995 | 87360.5497 | 245286.1502 | 180.7745 | 14380.6732 | 39298.0781 | 173.2701 |
| Serbia | 111485.0036 | 106436.5447 | -4.5284 | 113688.185 | 111758.8069 | -1.6971 | 18826.567 | 18163.1977 | -3.5236 |
| Seychelles | 624.9259 | 1064.768 | 70.3831 | 514.5498 | 920.3601 | 78.8671 | 96.1396 | 167.3473 | 74.067 |
| Sierra Leone | 58853.021 | 141592.2503 | 140.5862 | 61356.2084 | 152449.8361 | 148.4668 | 9912.8973 | 24352.9061 | 145.6689 |
| Singapore | 49476.7295 | 50159.1575 | 1.3793 | 61252.3653 | 56366.3644 | -7.9768 | 9423.6419 | 9037.5254 | -4.0973 |
| Slovakia | 59568.6774 | 70449.1077 | 18.2654 | 59167.7316 | 74993.9055 | 26.748 | 9893.0862 | 12097.4788 | 22.2822 |
| Slovenia | 29778.9735 | 25146.0284 | -15.5578 | 32955.425 | 27420.4235 | -16.7954 | 5264.5654 | 4370.487 | -16.9829 |
| Solomon Islands | 3079.2425 | 7621.6954 | 147.5185 | 2978.4312 | 7205.3228 | 141.9167 | 506.4986 | 1239.4001 | 144.6996 |
| Somalia | 114702.7776 | 381749.3274 | 232.8161 | 120314.9326 | 430854.4748 | 258.1056 | 19267.019 | 67029.7515 | 247.8989 |
| South Africa | 615822.249 | 1277527.842 | 107.4507 | 672596.3726 | 1479857.036 | 120.0216 | 105765.8773 | 223065.7737 | 110.9052 |
| South Sudan | 82512.185 | 160248.5943 | 94.212 | 85316.5568 | 171157.2496 | 100.6143 | 13791.7742 | 27327.677 | 98.1448 |
| Spain | 687667.9083 | 1003410.287 | 45.915 | 784653.3567 | 1242554.2 | 58.3571 | 124074.7842 | 187733.4713 | 51.3067 |
| Sri Lanka | 230434.917 | 277400.0514 | 20.3811 | 229062.0704 | 260590.7411 | 13.7642 | 38769.4052 | 45451.3831 | 17.2352 |
| Sudan | 324198.7384 | 849266.1188 | 161.9585 | 380969.1001 | 1014633.659 | 166.3296 | 58225.5486 | 153709.8566 | 163.9904 |
| Suriname | 6576.2558 | 14023.1902 | 113.2397 | 8203.5939 | 18315.3132 | 123.2596 | 1225.9768 | 2673.732 | 118.0899 |
| Sweden | 173242.1237 | 215577.418 | 24.4371 | 206568.6456 | 264210.9067 | 27.9047 | 31850.3747 | 40199.6821 | 26.2142 |
| Switzerland | 140268.7894 | 155966.12 | 11.1909 | 164220.972 | 183468.1222 | 11.7203 | 25481.1669 | 28233.3917 | 10.801 |
| Syrian Arab Republic | 175256.3169 | 296111.7359 | 68.9592 | 202577.4295 | 353313.6434 | 74.4092 | 31304.486 | 53506.7579 | 70.9236 |
| Taiwan (Province of China) | 180045.3356 | 230937.0713 | 28.2661 | 135788.3497 | 180125.1124 | 32.6514 | 26616.3156 | 34687.5952 | 30.3246 |
| Tajikistan | 49720.9366 | 124852.4741 | 151.1064 | 50365.4408 | 131815.7184 | 161.7186 | 8340.9981 | 21379.6743 | 156.3203 |
| Thailand | 645292.2278 | 748425.9097 | 15.9825 | 569854.0705 | 646214.1707 | 13.3999 | 102402.6127 | 117594.849 | 14.8358 |
| Timor-Leste | 7969.9557 | 14481.3171 | 81.6988 | 7405.2366 | 13704.2588 | 85.0617 | 1292.5076 | 2383.8024 | 84.4324 |
| Togo | 50495.2709 | 138057.82 | 173.4074 | 53388.5685 | 147940.5648 | 177.1016 | 8556.8783 | 23621.1756 | 176.049 |
| Tokelau | 15.0892 | 15.4701 | 2.5241 | 14.0484 | 14.7941 | 5.3081 | 2.4392 | 2.5374 | 4.0256 |
| Tonga | 834.5562 | 1019.8457 | 22.2022 | 739.1846 | 903.1585 | 22.1831 | 132.118 | 160.9942 | 21.8564 |
| Trinidad and Tobago | 18758.6765 | 26019.4113 | 38.706 | 22797.8922 | 32707.3869 | 43.4667 | 3449.8931 | 4848.7569 | 40.548 |
| Tunisia | 157261.6964 | 315461.7255 | 100.5967 | 188815.0777 | 394078.7311 | 108.7115 | 28844.4916 | 58529.6 | 102.9143 |
| Türkiye | 968521.2001 | 1774244.442 | 83.1911 | 1144119.797 | 2144473.994 | 87.4344 | 175648.9966 | 324714.5971 | 84.8656 |
| Turkmenistan | 38734.8835 | 63513.362 | 63.9694 | 39830.595 | 67451.0769 | 69.3449 | 6545.6023 | 10898.4186 | 66.4999 |
| Tuvalu | 106.3061 | 136.2846 | 28.2001 | 96.6172 | 132.5194 | 37.1592 | 17.04 | 22.573 | 32.4709 |
| Uganda | 331351.1863 | 980319.4736 | 195.8551 | 382947.6645 | 1161660.392 | 203.347 | 58827.1936 | 178091.5186 | 202.7367 |
| Ukraine | 779753.2994 | 764474.0081 | -1.9595 | 884896.5183 | 902058.8647 | 1.9395 | 137384.288 | 137272.9541 | -0.081 |
| United Arab Emirates | 22846.5997 | 134552.004 | 488.9367 | 26332.0084 | 156004.2225 | 492.4509 | 4059.573 | 23847.7006 | 487.4436 |
| United Kingdom | 1112440.487 | 1309063.659 | 17.6749 | 1327500.767 | 1592590.772 | 19.9691 | 203071.2283 | 240554.712 | 18.4583 |
| United Republic of Tanzania | 381726.3508 | 1027559.993 | 169.1876 | 394744.7938 | 1093172.237 | 176.9314 | 62976.078 | 174813.6134 | 177.5873 |
| United States of America | 4777645.654 | 8040511.588 | 68.2944 | 4796587.596 | 9742035.372 | 103.1035 | 807795.7798 | 1480344.876 | 83.2573 |
| United States Virgin Islands | 1380.8579 | 1013.5111 | -26.6028 | 1565.1614 | 1206.9779 | -22.8848 | 244.1477 | 183.3471 | -24.9032 |
| Uruguay | 37461.4382 | 58628.4767 | 56.5035 | 44469.3607 | 74278.1951 | 67.0323 | 6840.4867 | 11066.5152 | 61.7796 |
| Uzbekistan | 219042.24 | 454720.4713 | 107.5949 | 228255.9158 | 477720.9653 | 109.2918 | 37146.4039 | 77479.5582 | 108.5789 |
| Vanuatu | 1464.2532 | 3451.1543 | 135.6938 | 1379.272 | 3247.1199 | 135.4227 | 238.8266 | 559.4576 | 134.2526 |
| Venezuela (Bolivarian Republic of) | 214570.0991 | 356236.7792 | 66.0235 | 251487.3174 | 424740.2649 | 68.8913 | 38563.4139 | 64541.5566 | 67.3647 |
| Viet Nam | 638708.9103 | 1086527.248 | 70.1131 | 562911.9188 | 915666.744 | 62.6661 | 101444.6756 | 169474.9675 | 67.0615 |
| Yemen | 224325.4723 | 684565.4598 | 205.1662 | 274295.5751 | 833588.8014 | 203.9017 | 40636.0084 | 123887.6442 | 204.8716 |
| Zambia | 100906.8162 | 307912.7463 | 205.1456 | 97805.4626 | 314534.7269 | 221.5922 | 16273.3358 | 51079.9058 | 213.8871 |
| Zimbabwe | 103191.5269 | 207357.1868 | 100.944 | 93309.3485 | 202751.7372 | 117.2898 | 16118.0978 | 33631.0737 | 108.6541 |

**Table S3.** Prevalence, incidence and disability-adjusted life years of anxiety disorders in women of child-bearing age at the national level, from 1990 to 2021

|  | 1990 | | | 2021 | | |
| --- | --- | --- | --- | --- | --- | --- |
| Location | ASPR per 100 000  (95% uncertainty interval) | ASIR per 100 000  (95% uncertainty interval) | ASDR per 100 000  (95% uncertainty interval) | ASPR per 100 000  (95% uncertainty interval) | ASIR per 100 000  (95% uncertainty interval) | ASDR per 100 000  (95% uncertainty interval) |
| Afghanistan | 8133.49 (6207.41,10676.9) | 974.03 (707.7,1382.48) | 956.29 (619.65,1397.97) | 10405.71 (7158.14,14725.91) | 1224.49 (798.36,1842.36) | 1229.29 (751.5,1905.87) |
| Albania | 5387.78 (4026.29,7145) | 759.63 (543.76,1058.52) | 649.64 (404,969.13) | 7470.4 (5226.36,10502.13) | 1037.69 (702.33,1468.43) | 896.18 (534.81,1351.59) |
| Algeria | 8162.26 (6104.99,11104.74) | 974.23 (683.76,1380.42) | 975.54 (622.4,1451.09) | 9045.53 (6002.63,12905.11) | 1033.89 (690.87,1499.95) | 1070.77 (625.51,1656.43) |
| American Samoa | 5568.4 (4251.85,7346.76) | 773.05 (547.05,1102.65) | 673.11 (418.69,995.96) | 6419.59 (4308.3,9205.66) | 887.36 (576.63,1273.15) | 768.15 (470.36,1208.5) |
| Andorra | 8554.28 (6458.04,11258.88) | 956.94 (696.23,1328.19) | 1020.47 (662.46,1490.21) | 10264.19 (7126.99,13877.59) | 1122.08 (761.68,1618.53) | 1216.31 (721.32,1836.57) |
| Angola | 5328.03 (4019.78,7170.11) | 752.08 (547.11,1049.31) | 629.69 (389.76,925.21) | 6154.44 (4297.43,8807.16) | 880.45 (581.64,1273.14) | 728.34 (429.39,1134.79) |
| Antigua and Barbuda | 6922.34 (5161.19,9215.12) | 952.8 (688.39,1310.07) | 828.58 (511.67,1203.31) | 8651.29 (5995.38,12101.95) | 1160.12 (782.89,1633.59) | 1029.94 (615.53,1621.83) |
| Argentina | 8764.89 (6640.47,11593.04) | 1028.55 (746.39,1390.16) | 1045.44 (661.04,1481.58) | 11034.72 (7711.54,15175.44) | 1278.59 (859.08,1845.81) | 1311.69 (788.11,1913.82) |
| Armenia | 3823.76 (2896.25,5037.52) | 561.31 (407.13,768.33) | 459.17 (284.34,668.11) | 5303.2 (3663.53,7412.43) | 773.31 (520.78,1099.38) | 636.31 (381.86,997.56) |
| Australia | 10028.39 (7499.36,13363.08) | 1143.32 (824.56,1561.71) | 1184.72 (763.48,1726.26) | 10837.88 (7398.52,14903.76) | 1217.8 (833.45,1714.38) | 1276.52 (759.52,1917.26) |
| Austria | 9020.62 (6743.89,11823.33) | 984.21 (706.89,1340.82) | 1077.12 (682.23,1545.65) | 11140.42 (7625.36,15748.09) | 1191.01 (808.77,1729.91) | 1327.68 (773.73,2000.04) |
| Azerbaijan | 3421.92 (2568.58,4558.52) | 520.84 (380.27,703.92) | 412.23 (260.42,606.24) | 4564.16 (3159.72,6509.35) | 690.52 (463.65,1020.92) | 547.36 (322.61,842.94) |
| Bahamas | 6913.47 (5200.95,9305.07) | 954.26 (687.99,1325.01) | 827.49 (517.59,1225.73) | 8899.54 (6072.35,12354.08) | 1200.81 (793.79,1732.64) | 1057.08 (639.63,1594.11) |
| Bahrain | 8725.65 (6379.74,11782.09) | 996.05 (706.33,1435.29) | 1038.3 (640.05,1522) | 10174.76 (6872.16,14353.07) | 1146.61 (753.03,1689.82) | 1203.97 (690.26,1820.61) |
| Bangladesh | 4263.51 (3203.3,5690.66) | 687.12 (499.64,935.34) | 505.86 (320.1,739.58) | 5467.48 (3748.69,7578.71) | 871.45 (584.21,1250.75) | 647.42 (389.43,993.35) |
| Barbados | 6914.53 (5181.08,9010.21) | 946.52 (687.43,1270.7) | 826.85 (521.16,1205.22) | 8937.35 (6301.05,12427.37) | 1198.61 (802.97,1739.81) | 1062.53 (657.19,1644.03) |
| Belarus | 5301.87 (4003.37,6942.23) | 723.98 (516.1,986.76) | 636.94 (405.9,926.78) | 7839.16 (5453.13,10888.44) | 1068.35 (706.86,1482.55) | 935 (568.56,1443.2) |
| Belgium | 7433.73 (5602.22,9746.07) | 885.71 (641.29,1227.63) | 886.37 (564.11,1276.58) | 8846.55 (6146.06,12406.38) | 1046.21 (705.15,1528.34) | 1050.88 (652.23,1599.83) |
| Belize | 6708.27 (5078.04,8849.3) | 938.14 (667.97,1288.95) | 804.22 (511.88,1165.36) | 8279.6 (5711.78,11908.48) | 1144.69 (754.71,1683.38) | 986.65 (591.03,1505.39) |
| Benin | 4247.35 (3158.62,5632.12) | 579.78 (418.83,800.24) | 502.02 (317.21,736.72) | 4752.5 (3243.55,6853.23) | 646.09 (421.94,954.03) | 566.78 (326.91,891.87) |
| Bermuda | 7003.89 (5266.67,9310.16) | 947.67 (687.24,1255.9) | 839.59 (534.69,1241.67) | 8766.38 (6007.03,12199.98) | 1161.33 (751.66,1680.94) | 1045.38 (620.82,1560.53) |
| Bhutan | 4398.26 (3251.66,5836.23) | 700.8 (506.02,953.28) | 525.11 (326.53,764.73) | 4808.55 (3326.98,6644.05) | 756.33 (507.02,1085.77) | 574.19 (345.48,896.66) |
| Bolivia (Plurinational State of) | 8682.18 (6501.68,11683.69) | 1112.14 (796.61,1563.11) | 1035.65 (657.02,1506.86) | 12945.76 (9023.08,18376.72) | 1637.14 (1119.57,2410.14) | 1542.51 (966.14,2383.89) |
| Bosnia and Herzegovina | 5284.98 (4073.85,6921.64) | 747.83 (545.12,1018.83) | 635.09 (410.32,931.1) | 7258.17 (5023.24,10020.72) | 1018.27 (663.62,1444.7) | 866.67 (514.95,1307.24) |
| Botswana | 4995.97 (3742.22,6668.3) | 718.65 (510.58,990.46) | 595.82 (375.02,879.65) | 6832.79 (4642.2,9780.62) | 970.73 (640.69,1414.32) | 802.45 (493.85,1244.57) |
| Brazil | 9190.31 (7729.24,10943.67) | 1207.57 (907.89,1580.07) | 1086.97 (754.56,1491.32) | 16098.24 (13070.46,19305.15) | 1839.21 (1408.6,2410.57) | 1895.55 (1290.38,2642.6) |
| Brunei Darussalam | 4730.51 (3564.44,6299.28) | 673.98 (493.06,927.16) | 572.56 (366.53,837.57) | 5448.56 (3717.01,7718.98) | 770.71 (516.67,1107.04) | 657.14 (404.02,989.78) |
| Bulgaria | 5357.81 (4127.25,6915.45) | 751.75 (552.21,1004.2) | 642.43 (415.03,917.4) | 8107.71 (5595.69,11694.95) | 1135.51 (768.56,1630.96) | 966.58 (590.92,1483.62) |
| Burkina Faso | 4172.89 (3140.65,5611.54) | 573.83 (416.77,801.18) | 495.34 (323.43,719.82) | 4539.71 (3071.01,6405.51) | 625.04 (408.29,905.79) | 543.19 (326.52,818.77) |
| Burundi | 5401.01 (4006.98,7166.55) | 792.99 (570.11,1093.83) | 642.39 (408.45,950.97) | 6627.03 (4540.25,9415.4) | 975.04 (643.67,1401.95) | 793.31 (454.09,1222.69) |
| Cabo Verde | 4200.08 (3182.96,5507.64) | 575.14 (419.09,779.25) | 504.63 (321.59,745.27) | 5003.34 (3467.44,7092.29) | 688.9 (467.61,982.76) | 599.54 (357.09,922.75) |
| Cambodia | 5842.35 (4482.57,7900.33) | 849.39 (628.24,1169.26) | 695.75 (443.91,1029.04) | 7449.87 (5066.91,10449.2) | 1070.19 (723.76,1533.23) | 896.21 (522.22,1342.64) |
| Cameroon | 4282.77 (3234.11,5647.01) | 584.66 (425.2,796.95) | 507.16 (323.21,747.33) | 4742.99 (3161.32,6700.79) | 649.76 (417.39,931.15) | 566.31 (334.84,859.55) |
| Canada | 7989.09 (7255.44,8749.78) | 1050.37 (790.32,1352.81) | 953.8 (659.71,1293.99) | 8939.2 (6174.37,12362.06) | 1190.99 (824.16,1701.68) | 1059.25 (680.62,1600.89) |
| Central African Republic | 5493.76 (4095.06,7389.1) | 764.16 (553.55,1049.15) | 645.53 (406.61,942.99) | 6101.15 (4224.01,8835.57) | 849.06 (551.65,1273.34) | 721.7 (419.72,1126.27) |
| Chad | 4296.45 (3244.03,5634.43) | 585.53 (419.11,811.26) | 510.54 (329.3,757.3) | 4699.71 (3118,6733.96) | 630.49 (415.96,917.43) | 560.76 (332.12,857.32) |
| Chile | 10893.07 (9884.43,11918.11) | 1157.35 (859.82,1570.62) | 1295.49 (902.35,1722.08) | 13025.65 (8915.98,18092.16) | 1379.66 (922.76,1937.18) | 1540.7 (944.6,2346.08) |
| China | 4967.59 (4110.59,6052.25) | 688.85 (523.42,903.65) | 602.7 (408.67,843.06) | 4956.68 (4056.32,6016.96) | 698.35 (536.63,908.95) | 601.19 (412.75,833.41) |
| Colombia | 7174.91 (5373.99,9433.53) | 957.86 (679.69,1342.47) | 860.7 (542.82,1232.19) | 8915.32 (6038.94,12637.62) | 1173.28 (776.72,1703.8) | 1069.73 (630.08,1741.98) |
| Comoros | 5292.49 (3941.71,7065.24) | 783.56 (550.16,1082.99) | 630.81 (395.09,936.76) | 6491.8 (4597.45,9323.63) | 945.58 (640.23,1362.28) | 774.72 (465.47,1188.27) |
| Congo | 5164.14 (3914.09,6936.31) | 738.76 (537.82,1007.42) | 609.53 (394.99,901.86) | 5953.12 (4111.76,8300.57) | 847.76 (556.36,1220.98) | 701.12 (432.18,1088.37) |
| Cook Islands | 5536.62 (4233.33,7314.72) | 768.36 (554.18,1064.72) | 668.62 (427.86,985.74) | 6398.65 (4535.97,8969.51) | 881.79 (613.03,1302.49) | 768.09 (478.5,1222.42) |
| Costa Rica | 6581.89 (5023.8,8680.58) | 900.1 (645.6,1243.19) | 788.83 (501.12,1166.15) | 8354.86 (5580.66,11458.83) | 1139.09 (771.75,1640.34) | 998.89 (606.08,1510.61) |
| Cite d'Ivoire | 4291.22 (3286.75,5761.14) | 583.04 (421.76,804.88) | 505.85 (327.65,748) | 4636.74 (3233.66,6654.44) | 633.47 (426.52,940.69) | 552.35 (334.69,898.03) |
| Croatia | 5279.84 (4002.69,6886.25) | 740.92 (547.92,1001.39) | 633.59 (403.54,931.07) | 6950.3 (4631.32,9652.57) | 967.89 (628.35,1411.29) | 833.3 (490.12,1301.48) |
| Cuba | 6871.3 (5116.9,9106.52) | 940.18 (697.7,1290.06) | 819.23 (529.73,1187.48) | 8878.61 (6084.53,12157.02) | 1187.63 (786.9,1682.67) | 1057.02 (663.58,1562.57) |
| Cyprus | 9360.94 (7049.51,12212.86) | 1008.26 (737.14,1402.03) | 1118.59 (708.08,1607.55) | 10762.12 (7481.94,14897.92) | 1127.96 (760.07,1637.17) | 1282.27 (785.91,1905.04) |
| Czechia | 5018.12 (3823.82,6554.97) | 714.85 (522.31,983.04) | 601.47 (378.43,872.06) | 6591.6 (4740.51,9359) | 935.06 (633.43,1343.53) | 787.27 (481.86,1251.48) |
| Democratic People's Republic of Korea | 4953.78 (3817.07,6386.35) | 676.02 (502.42,898.92) | 601.05 (385.77,882.13) | 5158.69 (3560.31,7051.28) | 711.19 (476.64,1011.63) | 625.51 (372.23,954.94) |
| Democratic Republic of the Congo | 5255.04 (3989.83,7010.4) | 749.22 (531.88,1062.92) | 613.06 (398.07,892.03) | 5716.61 (3942.57,8279.82) | 818.72 (537.8,1182.69) | 674.73 (402.85,1073.24) |
| Denmark | 8000.85 (5992.49,10541.77) | 920.47 (667.53,1257.59) | 950.17 (607.44,1354.5) | 9671.37 (6573.19,13526.02) | 1110.67 (761.14,1553.95) | 1148.74 (710.35,1709.31) |
| Djibouti | 5163.28 (3844.2,7025.57) | 774.94 (551.04,1080.23) | 618.57 (390.91,902.81) | 6098.29 (4133.99,8743.6) | 888.5 (587.91,1297.58) | 728.65 (428.78,1143.4) |
| Dominica | 6886.22 (5199.97,9233.98) | 955.15 (689.27,1303.44) | 823.04 (523.09,1213.01) | 8749.3 (6179,12122.21) | 1186.44 (797.65,1690.54) | 1038.65 (630.23,1571.67) |
| Dominican Republic | 7024.45 (5241.27,9297.7) | 962.57 (696.31,1319.67) | 841.77 (529.06,1225.36) | 8366.37 (5703.78,11664.99) | 1130.09 (737.95,1636.55) | 997.08 (599.39,1498.51) |
| Ecuador | 8276.52 (6170.63,11012.92) | 1092.38 (767.92,1538.81) | 993.73 (635.98,1430.63) | 11074.74 (7726.23,15248.31) | 1424.78 (952.37,2069.84) | 1322.36 (828.99,2034.61) |
| Egypt | 7243.12 (5369.89,9764.95) | 896.59 (645.61,1225.96) | 862.75 (553.55,1256.11) | 8785.15 (6005.35,12168.39) | 1076.03 (725.23,1575.8) | 1045.72 (594.65,1608.64) |
| El Salvador | 6673.95 (5039.01,8919.44) | 916.65 (659.27,1277.82) | 797.22 (497.31,1169.67) | 8385.02 (5639.33,11691.13) | 1137.16 (748.38,1621.2) | 1003.1 (614.51,1568.66) |
| Equatorial Guinea | 5212.82 (3971.78,6957.13) | 742.59 (539.96,1019.73) | 611.75 (387.28,901.17) | 5838.39 (3847.13,8372.47) | 836.57 (541.64,1225.94) | 688.08 (415.61,1096.15) |
| Eritrea | 5479.27 (4125.3,7259.88) | 804.5 (578.25,1109.16) | 649.38 (414.31,962.46) | 6280.62 (4246.66,8951.17) | 910.71 (599.57,1330.89) | 751.9 (455.28,1203.56) |
| Estonia | 5204.74 (3980.99,6797.26) | 714.62 (519.42,949.66) | 622.79 (391.18,906.27) | 6961.03 (4690.99,9773.25) | 958.76 (646.62,1377.98) | 831.29 (497.79,1274.51) |
| Eswatini | 4972.91 (3756.28,6697.71) | 717.02 (508.16,1008.82) | 595.77 (377.54,884.11) | 7457.95 (5151.48,10637.88) | 1067.49 (704.33,1558.7) | 873.44 (540.73,1320.54) |
| Ethiopia | 6349.61 (5191.88,7809.46) | 927.26 (679.65,1201.71) | 755.13 (506.09,1076.67) | 6702.52 (5339.07,8384.46) | 1017.45 (744.91,1338.39) | 801.86 (533.51,1165.55) |
| Fiji | 5568.41 (4166.4,7408.06) | 777.22 (564.28,1064.11) | 669.04 (427.85,984.23) | 6691.06 (4583.32,9686.86) | 923.75 (608.37,1332.69) | 801.96 (477.97,1242.97) |
| Finland | 6094.78 (4622.87,7866.81) | 753.75 (558.99,1028.07) | 726.04 (478.73,1050.24) | 7078.73 (4799.64,9893.4) | 925.88 (618.33,1373.51) | 842.42 (497.53,1297.71) |
| France | 10588.98 (7983.54,13705.5) | 1072.66 (776.5,1452.11) | 1260.65 (800.1,1823.95) | 12799.47 (8764.64,17620.19) | 1294.15 (873.58,1829.11) | 1517.89 (920.04,2273.32) |
| Gabon | 5305.58 (3907.99,7006.24) | 751.89 (547.89,1013.21) | 625.69 (401.77,921.44) | 6430.06 (4337.54,9212.18) | 909.92 (595.14,1350.59) | 757.33 (465.14,1201.58) |
| Gambia | 4356.19 (3285.49,5867.55) | 587.49 (428.2,823.11) | 517.6 (324.47,769.05) | 5028.38 (3423.02,6968.55) | 682.55 (444.72,981.83) | 596.35 (360,921.73) |
| Georgia | 3422.66 (2596.31,4442.58) | 518.79 (384.52,699.51) | 412.45 (259.46,609.36) | 4599.54 (3151.3,6579.92) | 693.06 (452.14,1001.71) | 551.2 (321.72,855.09) |
| Germany | 9656.64 (7344.89,12516.61) | 1014.51 (728.97,1394.09) | 1148.09 (735.18,1656.14) | 12282.66 (8653.21,17409.49) | 1258.99 (853.1,1781.12) | 1455.59 (866.61,2202.51) |
| Ghana | 4101.82 (3137.75,5410.68) | 566.56 (412.59,777.35) | 485.83 (315.46,716.8) | 4618.04 (3244.6,6654.34) | 642.78 (436.93,948.1) | 550.37 (324.65,837.57) |
| Greece | 8789.47 (6728.01,11266.08) | 977.4 (716.43,1314.83) | 1047.44 (664.82,1506.63) | 11159.11 (7801.18,15310.4) | 1200.33 (810.91,1691.22) | 1320.55 (822.96,2037.9) |
| Greenland | 8523.12 (6289.84,11474.39) | 1117.42 (799.95,1524.91) | 1001.73 (632.93,1500.79) | 10999.73 (7274.3,15663.44) | 1439.03 (943.13,2061.2) | 1294.82 (731.19,1972.01) |
| Grenada | 6840.14 (5218.57,9022.64) | 950.34 (681.73,1268.94) | 816.26 (506.54,1192.58) | 8660.88 (5883.29,12256.82) | 1171.06 (768.67,1686.03) | 1030.67 (623.58,1587.77) |
| Guam | 5539.01 (4213.13,7310.1) | 770.37 (559.03,1038.72) | 669.96 (425.52,956.38) | 6746.05 (4586.11,9337.41) | 930.13 (623.16,1316.92) | 812.33 (489.72,1261.5) |
| Guatemala | 6562.25 (4829.81,8630.25) | 909.8 (649.89,1270.71) | 776.94 (491.2,1102.72) | 8388.76 (5793.47,11384.42) | 1153.74 (754.4,1664.75) | 995.47 (598.71,1514.81) |
| Guinea | 4310.82 (3276.09,5711.85) | 587.34 (424.88,798.23) | 511.37 (325.56,750.19) | 5059.02 (3442.84,7257.47) | 685.78 (447.93,999.07) | 602.81 (360,965.05) |
| Guinea-Bissau | 4181.35 (3140.42,5480.64) | 575.3 (419.98,782.73) | 496.57 (316.11,732.96) | 4765.08 (3236.64,6743.62) | 657.37 (435.11,955.23) | 565.51 (343.06,878.96) |
| Guyana | 6961.75 (5212.2,9196.51) | 962.15 (690.02,1341.45) | 826.67 (507.33,1186.57) | 9357.96 (6344,13282.98) | 1271.71 (864.08,1870.18) | 1104.53 (638,1737.26) |
| Haiti | 7060.8 (5317.58,9333.23) | 965.72 (693.08,1319.11) | 829.85 (526.78,1211.75) | 8626.49 (5932.9,11962.03) | 1163.62 (777.08,1670.35) | 1010.37 (606.7,1512.75) |
| Honduras | 6395.52 (4848.66,8440.38) | 894.93 (653.39,1250.22) | 766.61 (482.02,1111.77) | 8839.51 (6149.16,12429.28) | 1225.44 (795.87,1772.37) | 1056.73 (636.84,1639.88) |
| Hungary | 5219.73 (3963.96,6815.85) | 734.7 (543.19,970.02) | 624.77 (397.88,909) | 6807.53 (4730.23,9728.52) | 951.83 (642.1,1367.67) | 812.34 (495.69,1247.17) |
| Iceland | 8342.82 (6308.05,10976.89) | 952.72 (685.2,1313.6) | 997.04 (638.25,1437.05) | 9022.4 (6152.49,12794.92) | 1019.01 (688.54,1464.26) | 1073.89 (628.79,1627.05) |
| India | 4544.47 (3750.12,5513.5) | 729.05 (564.68,949.95) | 534.63 (364.54,740.24) | 5621.82 (4630.89,6704.2) | 906.21 (705.41,1148.32) | 663.71 (460.82,910.21) |
| Indonesia | 5206.28 (4279.56,6326.28) | 807.11 (618.06,1042.98) | 628.43 (424.35,878.15) | 6894.36 (5662.82,8351.44) | 1041.81 (791.34,1346.02) | 831.55 (563.09,1184.02) |
| Iran (Islamic Republic of) | 11193.79 (9313.06,13571.16) | 1256.94 (903.55,1709.65) | 1331.85 (899.04,1867.39) | 13282.8 (10843.69,15918.35) | 1394.33 (1036.5,1883.04) | 1566.11 (1033.85,2188.63) |
| Iraq | 8973.03 (6597.82,12019.88) | 1022.73 (728.46,1468.81) | 1067.01 (669.84,1528.32) | 10429.24 (7140.36,14831.93) | 1155.07 (751.47,1756.47) | 1237.62 (729.02,1891.26) |
| Ireland | 10239.49 (7648.82,13528) | 1069.24 (779.92,1492.46) | 1221.78 (776.09,1781.32) | 12326.07 (8600.83,16989.22) | 1253.62 (829.48,1771) | 1459.02 (883.45,2251.19) |
| Israel | 5767.99 (4452.44,7553.67) | 776.13 (575.51,1054.31) | 690.7 (435.17,997.73) | 6678.49 (4560.26,9288.8) | 889.43 (599.34,1276.4) | 797.11 (485.5,1252.41) |
| Italy | 9729.47 (8169.98,11602.53) | 1112.49 (833.11,1496.28) | 1157.42 (796.59,1607.81) | 11091.64 (9087.6,13210.65) | 1287.03 (961.78,1692.83) | 1318.11 (876.36,1833.75) |
| Jamaica | 6578.98 (4924.63,8754.32) | 928.24 (658.54,1266.76) | 789.16 (493.93,1165.86) | 8679.82 (5851.9,12321.17) | 1192.65 (791.28,1743.28) | 1036.6 (588.73,1600.31) |
| Japan | 4136.63 (3462.2,4954.79) | 612.89 (476.14,779.12) | 497.67 (338.9,690.1) | 4944.96 (4105.15,5951.73) | 729.05 (561.87,930.01) | 594.39 (401.52,827.93) |
| Jordan | 8465.59 (6323.18,11318.78) | 998.03 (710.98,1431.41) | 1011.64 (627.45,1475.18) | 9813.68 (6542.47,13610.34) | 1128.16 (735.97,1661.2) | 1166.97 (670.38,1855.42) |
| Kazakhstan | 3123.76 (2377.68,4161.96) | 487.28 (354.63,651.13) | 374.76 (231.49,549.01) | 3799.52 (2612.75,5269.96) | 587.04 (403.13,842.26) | 454.02 (271.5,693.38) |
| Kenya | 5248.31 (4312.13,6413.33) | 817.68 (611.82,1053.94) | 628.24 (424.22,874.32) | 6248.69 (5114.32,7570.83) | 958.69 (722.78,1225.65) | 746.28 (504.29,1038.14) |
| Kiribati | 5553.85 (4235.64,7462.74) | 770.68 (551.86,1058.22) | 663.57 (430.87,966.23) | 6465.26 (4464.76,9260.86) | 894.1 (603.27,1298.32) | 772.22 (482,1161.57) |
| Kuwait | 7948.56 (5772.38,10639.82) | 939.58 (673.91,1324.22) | 948.67 (600.93,1403.71) | 8309.77 (5726.56,11542.37) | 942.05 (623.58,1373.12) | 982.57 (594.37,1482.82) |
| Kyrgyzstan | 3062.88 (2316.4,4131.47) | 482.51 (350.44,664.9) | 368.67 (234.45,538.56) | 3990.88 (2707.16,5847.16) | 623.54 (409.03,906.64) | 478.73 (281.05,728.74) |
| Lao People's Democratic Republic | 6279.95 (4805.55,8296.22) | 892.83 (648.54,1209.18) | 752.69 (486.12,1077.14) | 7683.03 (5214.14,10898.03) | 1070.31 (698.41,1550.06) | 925.86 (557.56,1413.12) |
| Latvia | 5462.71 (4120.24,7189.51) | 738.49 (537.48,999.94) | 654.12 (413.33,957.26) | 7926.58 (5427.22,11080.17) | 1069.96 (723.76,1533.94) | 944.77 (572.66,1426.42) |
| Lebanon | 10155.25 (7723.88,13453.1) | 1065.65 (766.07,1470.73) | 1201.76 (758,1726.14) | 13782.3 (9470.62,19109.96) | 1400.36 (951.76,1978.64) | 1622.12 (950.09,2443.11) |
| Lesotho | 4990.18 (3782.22,6630.98) | 717.39 (529.14,985.33) | 593.29 (379.63,863.92) | 7234.74 (5065.41,10219.73) | 1031.3 (670.91,1505.53) | 844.03 (518.16,1292.58) |
| Liberia | 4437.45 (3251.35,5832.74) | 596.61 (433.78,825.59) | 515.21 (326.78,772.15) | 4858.27 (3296.29,6857.11) | 652.27 (436.28,950.45) | 568.1 (339.5,909.22) |
| Libya | 8750.55 (6584.66,11674.44) | 1015.47 (724.2,1455.85) | 1046.08 (662.16,1522.33) | 10266.18 (7062.13,14597.64) | 1140.44 (750.18,1660.22) | 1213.28 (739.27,1840.67) |
| Lithuania | 6345.32 (4837.97,8404.37) | 805.48 (577.13,1080.89) | 759.22 (487.77,1114.23) | 9065.03 (6285.29,12768.56) | 1150.44 (761.88,1669.77) | 1081.71 (656.3,1681.11) |
| Luxembourg | 8416.62 (6327.74,11250.84) | 940.77 (677.63,1301.27) | 1001.21 (639.54,1439.69) | 9844.82 (6744.83,13847.56) | 1095.61 (723.32,1572.53) | 1171 (715.24,1776.61) |
| Madagascar | 5607.75 (4180.98,7422.78) | 816.88 (599.59,1109.27) | 664.65 (412.97,977.9) | 6749.04 (4582.96,9651.47) | 973.08 (652.61,1437.2) | 803.92 (471.87,1257.18) |
| Malawi | 5531.53 (4175.2,7517.27) | 813.53 (588.91,1141.55) | 648.64 (413.54,954.26) | 6842.55 (4630.28,9853.18) | 1006.2 (677.37,1479.89) | 815.31 (473.12,1274.59) |
| Malaysia | 6573.68 (4909.03,8746.19) | 920.99 (679.52,1266.36) | 792.16 (500.34,1188.47) | 8437.45 (5801.93,12153.68) | 1164.57 (793.55,1741.56) | 1013.37 (608.2,1564.7) |
| Maldives | 5341.48 (4083.13,7062.58) | 800.26 (572.44,1100.07) | 637.85 (401.1,932.04) | 6626.15 (4546.38,9313.37) | 974.74 (662.86,1434.64) | 790.69 (462.64,1229.09) |
| Mali | 3851.67 (2925.95,5006.55) | 546.01 (404.67,746.09) | 456.95 (291.65,671.1) | 4433.93 (3065.82,6363.95) | 617.74 (400.5,923.77) | 528.09 (319.76,854.94) |
| Malta | 9212.48 (7079.67,12040.59) | 986.89 (721.39,1341.64) | 1100.64 (695.53,1591.9) | 10507.59 (7125.96,14631.45) | 1112.1 (718.35,1641.54) | 1247 (762.66,1953.95) |
| Marshall Islands | 5579.23 (4220.79,7325.81) | 778.11 (563.35,1041.47) | 672.21 (430.03,983.62) | 6388.74 (4520.19,9238.5) | 879.28 (597.18,1291.39) | 764.45 (455.58,1199.74) |
| Mauritania | 3930.06 (3014.2,5252.95) | 551.28 (392.18,748.85) | 468.65 (295.09,694.25) | 4419.79 (3144.43,6318.58) | 620.42 (415.77,908.5) | 529.99 (320.23,822.34) |
| Mauritius | 5520.52 (4182.3,7282.47) | 819.19 (590.45,1125.94) | 660.6 (417.7,971.92) | 6862.02 (4759.79,9522) | 1005.13 (676.26,1466.47) | 819.19 (494.06,1270.02) |
| Mexico | 4425.18 (3679.91,5369.31) | 684.16 (534.38,873.55) | 530.59 (360.45,742.51) | 7826.16 (6386.77,9422.67) | 1145.94 (881.19,1482.3) | 931.6 (618.69,1302.18) |
| Micronesia (Federated States of) | 5581.81 (4250.15,7307.6) | 775.39 (568.92,1063.35) | 671.89 (422.53,972.28) | 6422.69 (4461.25,9152.79) | 885.7 (594.91,1285.67) | 771.52 (471.55,1155.73) |
| Monaco | 8398.7 (6183.55,10798.25) | 920.45 (678.48,1256.68) | 999.6 (626.01,1427.36) | 10433.4 (7322.29,14482.89) | 1147.11 (760.17,1694.19) | 1238.11 (753.74,1905.58) |
| Mongolia | 3122.69 (2345.21,4173.34) | 487.23 (357.34,667.18) | 374.46 (240.84,548.19) | 3467.42 (2411.1,4884.03) | 535.75 (352.32,773.15) | 414.56 (251.38,633.61) |
| Montenegro | 5372.3 (4047.32,7016.65) | 755.58 (546.9,1025.77) | 646.94 (422.13,953.51) | 7661.36 (5138.49,10698.54) | 1069.18 (694.44,1540.32) | 915.92 (544.73,1398.42) |
| Morocco | 8127.58 (6023.27,10806.16) | 962.5 (684.91,1367.16) | 965.58 (603.98,1381.62) | 9937.31 (6841.05,13949.19) | 1137.96 (775.9,1601.37) | 1171.78 (715.88,1847.88) |
| Mozambique | 5689.33 (4295.9,7537.46) | 818.73 (586.66,1134.48) | 666.42 (425.31,974.71) | 7001.72 (4787.63,9978.05) | 1016.56 (677.13,1505.4) | 815.03 (496.83,1274.28) |
| Myanmar | 5467.64 (4141.45,7216.37) | 813.93 (596.34,1136.51) | 655.54 (419.31,957.38) | 7374.27 (5078.61,10004.28) | 1077.21 (705.46,1530.53) | 884.17 (514.55,1356.11) |
| Namibia | 4910.75 (3725.86,6512.31) | 710.57 (515.73,994.63) | 586.14 (377.43,857.48) | 7234.66 (4983.55,10131.05) | 1030.2 (692.91,1474.7) | 857.91 (539.61,1294.41) |
| Nauru | 5550.08 (4226.8,7442.25) | 771.64 (560.41,1053.68) | 668.35 (426.76,994.51) | 6438.19 (4474.38,9132.16) | 886.48 (601.81,1276.76) | 774.04 (448.58,1227.66) |
| Nepal | 4306.53 (3271.46,5672.66) | 684.24 (500.89,933.17) | 508.04 (323.5,752.97) | 5857.3 (3931.05,8459.73) | 932.84 (620.71,1327.25) | 695.14 (406.79,1117.58) |
| Netherlands | 9576.04 (7220.45,12320.52) | 960.73 (694.77,1332.03) | 1146.81 (727.52,1672.77) | 12363.56 (8519.25,17124.37) | 1206.24 (832.08,1742.31) | 1473.53 (905.87,2188.35) |
| New Zealand | 12293.91 (10113.59,15015.16) | 1322.76 (961.7,1817.05) | 1448.29 (985.73,2035.79) | 13678.52 (10441.35,17565.05) | 1483.93 (1065.22,2014.63) | 1616.42 (1038.31,2341.22) |
| Nicaragua | 6705.78 (5040.6,8842.08) | 919.74 (650.14,1272.26) | 802.52 (505.22,1170.15) | 8471.14 (5867.81,11860.75) | 1150.86 (781.99,1636.1) | 1012.04 (601.85,1561.31) |
| Niger | 4007.49 (3114.01,5280.8) | 559.79 (405.16,758.62) | 475.51 (302.71,697.01) | 4279.8 (3008.08,6141.74) | 587.76 (387.92,883.36) | 514.6 (295.41,854.29) |
| Nigeria | 4249.31 (3522.09,5205.68) | 597.46 (442.97,780.84) | 503.1 (344.98,704.11) | 4466.06 (3691.14,5481.53) | 627.11 (472.66,818.86) | 532.24 (356.13,748.68) |
| Niue | 5562.36 (4252.42,7197.52) | 772.1 (568.89,1052.2) | 669.74 (430.37,988.72) | 6385.1 (4409.85,8882.17) | 879.21 (587.9,1258.39) | 765.38 (442.48,1149.77) |
| North Macedonia | 5330.52 (4119.07,7031.75) | 752.08 (556.68,1012.5) | 639.84 (404.03,925.37) | 7910.48 (5284.87,10835.18) | 1105.38 (730.03,1576.8) | 946.64 (553.06,1421.35) |
| Northern Mariana Islands | 5537.82 (4199.74,7324.8) | 772.46 (548.41,1041.37) | 670.68 (412.39,989.61) | 6876.14 (4829.94,9519.82) | 945.58 (619.27,1346.49) | 827.08 (506.23,1264.37) |
| Norway | 11915.99 (9883.68,14251.02) | 1243.16 (924.35,1707.62) | 1422.57 (975.11,1995) | 11824.78 (9601.35,14499.02) | 1287.81 (964,1750.75) | 1408.45 (944.85,1970.82) |
| Oman | 8421.31 (6217.94,11041.35) | 980.53 (701.94,1363.13) | 1004.34 (644.6,1452.9) | 9913.88 (6845.9,13882.01) | 1117.65 (736.55,1644.92) | 1177.85 (709.45,1813.51) |
| Pakistan | 4602.21 (3803.31,5531.24) | 742.17 (559.99,963.49) | 545.88 (373.87,753.78) | 5583.56 (4387.89,6995.63) | 899.49 (648.16,1183.06) | 661.15 (429.83,944.81) |
| Palau | 5536.43 (4182.51,7274.44) | 770.47 (560.49,1033.69) | 666.94 (423.51,968.6) | 6269.07 (4399.17,8618.42) | 859.08 (569.89,1185.73) | 749.94 (450.41,1197.96) |
| Palestine | 8927.36 (6625.57,11998.37) | 1022.19 (723.25,1437.36) | 1063.76 (672.94,1572.67) | 10761.77 (7389.35,15084.2) | 1211.59 (809.98,1783.46) | 1278.17 (761.04,2006.39) |
| Panama | 6084.32 (4603.16,8132.57) | 859.01 (622.91,1197.45) | 730.59 (460.2,1069.28) | 7785.09 (5177.68,11193.23) | 1083.8 (727.1,1613.37) | 928.64 (551.39,1405.43) |
| Papua New Guinea | 5564.51 (4219.26,7489.22) | 772.57 (560.75,1063.08) | 663.5 (425.41,992.92) | 6157.93 (4187.51,8786.05) | 851.98 (569.15,1247.79) | 738.55 (429.81,1158.67) |
| Paraguay | 11280.46 (8596.04,15182.32) | 1304.02 (951.33,1767.84) | 1345.05 (859.47,1990.33) | 14802.02 (10367.85,20058.43) | 1668.54 (1120.03,2391.7) | 1754.89 (1101.37,2711.87) |
| Peru | 8555.33 (6494.1,11379.44) | 1105.31 (803.24,1538.94) | 1025.46 (644.53,1478.46) | 12435.96 (8273.22,17645.72) | 1572.77 (1022.26,2372.38) | 1488.82 (868.18,2274.9) |
| Philippines | 6014.86 (4964.04,7321.46) | 905.9 (689.59,1184.43) | 722.98 (493.98,1000.4) | 7769.32 (6453.52,9354.53) | 1152.67 (885.34,1486.57) | 933.39 (633.67,1319.37) |
| Poland | 5001.25 (4121.99,5960.3) | 738.05 (565.91,949.27) | 598.69 (411.91,829.06) | 6576.12 (5397.18,7870.02) | 966.25 (747.78,1239.54) | 787.48 (529.57,1101.73) |
| Portugal | 12010.9 (9231.05,15791.82) | 1146.86 (831.52,1570.05) | 1427.55 (933.39,2076.16) | 15047.65 (10401.09,20397.11) | 1373.72 (922.12,1951.5) | 1781.15 (1072.31,2714.5) |
| Puerto Rico | 7006.93 (5204.48,9191.42) | 952.02 (694.82,1282.03) | 838.4 (525.49,1214.37) | 8442.91 (5791.71,11822.75) | 1128.9 (757.46,1628.58) | 1007.75 (603.46,1562.94) |
| Qatar | 8192.15 (5964.33,10922) | 948.25 (678.05,1302.61) | 970.93 (609.15,1433.07) | 8862.43 (5754.35,12826.02) | 995.53 (628.8,1457.56) | 1043.77 (605.9,1593.39) |
| Republic of Korea | 5496.98 (4179.88,7392.34) | 739.91 (535.43,1023.04) | 663.06 (428.46,973.79) | 6055.72 (4005.5,8419.53) | 800.72 (511.39,1126.09) | 729.53 (428.73,1143.37) |
| Republic of Moldova | 5748.92 (4250.22,7620.18) | 763.94 (553.57,1054.99) | 689.68 (432.66,1008.53) | 7456.61 (5090.68,10212.52) | 988.29 (657.96,1422.74) | 891.33 (521.83,1375.43) |
| Romania | 4987.09 (3747.76,6493.01) | 719.32 (528.84,975.66) | 596.05 (376.84,858.73) | 6908.32 (4548.77,9527.65) | 985.23 (645.07,1424.58) | 825.03 (500.55,1232.07) |
| Russian Federation | 5051.57 (4197.66,6112.57) | 726.15 (564.71,940.89) | 603.93 (412.06,839.16) | 6760.45 (5627.07,8028.17) | 966.41 (745.38,1234.5) | 804.35 (545.14,1106.44) |
| Rwanda | 5366.99 (3964.87,7106.07) | 793.06 (571.85,1094.78) | 639.22 (404.68,945.23) | 6562.85 (4433.84,9199.45) | 957.52 (645.93,1402.76) | 782.16 (477.27,1205.79) |
| Saint Kitts and Nevis | 6871.39 (5099.26,9158.83) | 956.62 (687.88,1326.56) | 818.27 (507.68,1193.91) | 8377.58 (5684.65,11660.21) | 1125.34 (750.78,1615.46) | 995.55 (585.53,1507.96) |
| Saint Lucia | 6868.07 (5207.26,9110.32) | 954.12 (677.65,1317.87) | 818.06 (509.6,1188.2) | 9174.06 (6260.33,12780.91) | 1235.15 (812.06,1756.28) | 1086.7 (659.15,1669.25) |
| Saint Vincent and the Grenadines | 6829.01 (5134.56,9160.43) | 951.07 (680.34,1313.81) | 817.35 (510.82,1208.27) | 8690.2 (5857.74,12022.3) | 1171.11 (790.78,1698.62) | 1031.7 (625.32,1565.03) |
| Samoa | 5587.72 (4263.56,7453.6) | 775.74 (563.49,1072.21) | 675.54 (437.94,1013.47) | 6476.17 (4430.76,8969.49) | 895.11 (586.8,1274.21) | 779.13 (462.97,1197.36) |
| San Marino | 8643.96 (6604.87,11335.86) | 965.92 (703.67,1351.52) | 1031.51 (657.01,1494.23) | 10609.46 (7321.12,14588.31) | 1165.12 (792.99,1657.28) | 1260.49 (778.2,1885.62) |
| Sao Tome and Principe | 4224.88 (3118.94,5604.9) | 579.74 (419.96,813.17) | 506.48 (322.96,743.34) | 4591.16 (3103.95,6410.45) | 632.64 (423.55,903.88) | 549.02 (328.93,831.86) |
| Saudi Arabia | 8106.15 (5959.47,11048.76) | 963.09 (685.14,1367.68) | 967.22 (612.6,1403.43) | 8854.65 (6132.98,12569.53) | 1010.68 (669.72,1508.61) | 1047.54 (632.82,1580.69) |
| Senegal | 4079.43 (3087.96,5358.06) | 564.42 (409.94,776.88) | 484.16 (305.97,713.08) | 4972.16 (3431.75,7163.54) | 689.87 (447.41,984.22) | 591.77 (351.5,938.15) |
| Serbia | 5108.2 (3941.19,6590.68) | 723.88 (531.19,980.25) | 612.83 (377.47,887.17) | 6754.11 (4627.64,9430.97) | 960.33 (638.43,1359.83) | 811.06 (493.68,1253.1) |
| Seychelles | 5364.77 (4126.11,7031.73) | 799.34 (576.88,1062.71) | 649.24 (418.53,952.08) | 7169.75 (5092.64,9877.51) | 1046.77 (715.7,1502.33) | 863.08 (501.05,1344.47) |
| Sierra Leone | 4554.08 (3422.37,5897.98) | 606.74 (434.02,825.73) | 541.35 (340.34,788.83) | 4724.26 (3216.07,6805.17) | 629.7 (430.84,942.73) | 563.31 (344.28,908.08) |
| Singapore | 4593.59 (3491.89,6092.18) | 652.53 (468.46,903.22) | 554.13 (348.32,815.41) | 4992.1 (3414.44,6966.61) | 705.26 (451.36,1017.03) | 602.43 (358.15,919.45) |
| Slovakia | 5232.92 (3932.05,6789.83) | 737.67 (529.43,1012.58) | 626.58 (396.24,915.32) | 7189.2 (4867.48,10061.39) | 1006.5 (673.3,1454.58) | 860.04 (527.51,1335.66) |
| Slovenia | 5200.11 (3990.53,6793.2) | 736.44 (539.54,995.28) | 623.28 (395.34,899.75) | 6555.82 (4259.29,9167.86) | 922.19 (604.12,1288.33) | 785.53 (449.56,1185.35) |
| Solomon Islands | 5589.76 (4272.61,7315.15) | 776.99 (556.64,1070.64) | 673.69 (428.64,988.41) | 6472.28 (4437.06,9252.53) | 894.06 (594.37,1329.79) | 776.09 (466.51,1225.09) |
| Somalia | 5239.22 (3927.88,6916.82) | 774.28 (558.37,1051.48) | 616.95 (384.37,886.62) | 6644.53 (4529.42,9548.94) | 1001.11 (655.53,1442.94) | 787.93 (479.35,1190.93) |
| South Africa | 5621.5 (4644.99,6849.33) | 810.95 (599.46,1059.34) | 667.26 (453.48,926.67) | 7556.67 (6107.18,9112.73) | 1070.1 (812.44,1412.91) | 880.4 (597.62,1228.41) |
| South Sudan | 6326.79 (4700.7,8404.38) | 885.23 (624.93,1224.92) | 749 (473.49,1089.05) | 7021.65 (4835.62,9813.02) | 974.9 (630.38,1432.05) | 832.94 (486.1,1312.6) |
| Spain | 7926.11 (6070.4,10409.7) | 939.41 (684.79,1326.75) | 948.02 (599.03,1380.18) | 10078.87 (7002.76,13995.16) | 1125.13 (747.28,1597.01) | 1197.36 (710.61,1852.81) |
| Sri Lanka | 5541.9 (4264.43,7295.25) | 818.23 (605.89,1109.67) | 664.4 (424.39,992.88) | 7177.64 (4884.52,9943.98) | 1043.3 (702.49,1477.76) | 862.43 (517.97,1316.05) |
| Sudan | 8168.45 (6163.95,10840.98) | 965.66 (680.56,1381.94) | 968.4 (618.96,1426.07) | 9767.33 (6579.6,14275.02) | 1141.75 (770.98,1714.22) | 1159.86 (714.33,1852.22) |
| Suriname | 6784.62 (5073.68,9090.58) | 935.96 (690.16,1310.53) | 807.76 (510.86,1171.7) | 9310.89 (6514.4,12918.16) | 1271.17 (841.9,1827.42) | 1098.09 (659.5,1636.11) |
| Sweden | 7998.12 (6671.45,9562.96) | 980.26 (748.3,1295.93) | 955.2 (659.04,1323.13) | 9348.14 (7172.79,12078.59) | 1143.04 (809.12,1612.45) | 1112.46 (714.65,1617.54) |
| Switzerland | 10575.65 (7929.06,13703.89) | 1050.66 (759.43,1445.78) | 1255.71 (800.71,1799.43) | 11794.07 (8447.47,16932.69) | 1165.83 (787.93,1655.03) | 1397.63 (857.6,2084.83) |
| Syrian Arab Republic | 8837.57 (6554.18,11777.77) | 1021.38 (722.07,1465.72) | 1053.12 (661.57,1547.03) | 10453.18 (7196.85,14474.34) | 1175.02 (793.9,1733.59) | 1235.24 (756.39,1925.77) |
| Taiwan (Province of China) | 5350.16 (4158.7,6851.34) | 683.72 (500.79,903.64) | 651.2 (424.02,948.43) | 5214.58 (3545.14,7341.66) | 719.87 (477.99,1054.63) | 629.94 (384.58,975.54) |
| Tajikistan | 3303.95 (2486.81,4467.08) | 509.3 (367.22,699.45) | 398.66 (255.11,581.57) | 4356.57 (2939.35,6259.86) | 662.71 (427.26,974.17) | 524.22 (307.17,826.53) |
| Thailand | 5083.67 (3826.78,6695.31) | 772.13 (552.18,1030.6) | 611.17 (386.75,880.2) | 6028.47 (4172.25,8543.9) | 892.1 (597.27,1319.23) | 723.87 (429.85,1138.2) |
| Timor-Leste | 5426.36 (4145.99,7236.43) | 809.38 (579.24,1113.84) | 650.4 (416.91,944.63) | 6539.54 (4511.08,9099.74) | 977.62 (651.66,1402.7) | 792.57 (476.15,1230.21) |
| Togo | 4507.45 (3408.55,6028.68) | 601.3 (429.38,840.37) | 535.41 (341.95,774.17) | 4858.6 (3334.25,6979.54) | 654.05 (428.32,958.81) | 578.27 (348.71,893.75) |
| Tokelau | 5563.93 (4238.54,7349.59) | 771.92 (564.67,1038.7) | 668.81 (430.88,953.16) | 6380.43 (4476.8,8812.88) | 878.25 (585.82,1261.66) | 767.01 (455.41,1155.93) |
| Tonga | 5584.62 (4303.07,7296.04) | 774.05 (567.49,1061.65) | 675.9 (436.05,973.73) | 6470.63 (4346.12,9028.44) | 889.46 (582.75,1300.82) | 780.29 (454.83,1202.64) |
| Trinidad and Tobago | 6512 (4887.4,8726.73) | 919.36 (673.93,1269.35) | 775.1 (489.76,1124.51) | 9099.1 (6178.72,13092.53) | 1255.35 (833.29,1790.34) | 1078.14 (663.67,1663.88) |
| Tunisia | 8488.1 (6297.32,11232.71) | 982.46 (698.05,1397.77) | 1014.96 (653.93,1472.78) | 11255.74 (7793.26,15789.75) | 1260.85 (819.39,1866.34) | 1326.36 (802.26,2047.26) |
| Türkiye | 6635.27 (5998.53,7287.61) | 835.07 (636.48,1073.38) | 792.61 (538.24,1071.88) | 8828.56 (5794.76,12219.8) | 1069.83 (723.9,1562.17) | 1048.21 (599.39,1631.9) |
| Turkmenistan | 3243.55 (2440.7,4351.89) | 502.94 (367.77,684.31) | 389.46 (250.85,584.28) | 4171.65 (2896.15,5733.32) | 641.2 (435.07,921.79) | 500.75 (285.54,771.76) |
| Tuvalu | 5497.54 (4192.63,7118.27) | 765.32 (558.7,1017.32) | 660.58 (423.05,956.26) | 6438.67 (4404.22,9458.01) | 888.69 (584.24,1331.43) | 774.26 (460.92,1192.69) |
| Uganda | 5277.83 (3895.98,7214.49) | 790.31 (566.78,1100.79) | 623.32 (389.24,925.85) | 6499.04 (4495.83,9205.56) | 965.38 (653.56,1392.99) | 776.96 (447.07,1192.71) |
| Ukraine | 4641.73 (3837.33,5602.87) | 675.25 (514.42,882.03) | 554.93 (372.52,782.6) | 6159.63 (4407.15,8247.45) | 896.72 (595.09,1243.14) | 732.58 (465.92,1082.36) |
| United Arab Emirates | 7487.06 (5924.73,9435.04) | 913.41 (656.56,1270.41) | 889.16 (590.24,1256.06) | 8823.54 (5953.19,12526.9) | 1011.75 (673.52,1479.45) | 1036.8 (626.98,1620.36) |
| United Kingdom | 6828.61 (5666.45,8361.37) | 882.71 (672.14,1172.73) | 810.61 (555.99,1129.18) | 8457.23 (6940.51,10174.49) | 1087.5 (824.04,1436.38) | 1001.5 (688.84,1388.89) |
| United Republic of Tanzania | 5517.24 (4171.96,7486.49) | 814.07 (587.75,1140.49) | 644 (410.75,935.72) | 6567.25 (4462.91,9406.21) | 956.41 (615.76,1430.75) | 780.6 (471.03,1249.5) |
| United States of America | 9215.04 (7600.58,11107.58) | 1189.69 (919.92,1535.99) | 1092.87 (749.88,1530.82) | 12495.14 (10387.69,14973) | 1581.97 (1191.55,2038.6) | 1463.9 (1013.06,2046.84) |
| United States Virgin Islands | 6981.89 (5211.99,9132.9) | 945.02 (685.18,1297.2) | 833.4 (518.45,1215.25) | 8754.43 (6120.75,11900.22) | 1169.92 (777.79,1683.75) | 1042.03 (628.83,1562.82) |
| Uruguay | 9385.12 (7108.55,12525.46) | 1062.9 (771.11,1488.42) | 1121.81 (703.68,1614.61) | 12198 (8549.77,16818.59) | 1364.38 (896.58,1925.05) | 1448.51 (871.74,2234.47) |
| Uzbekistan | 3064.2 (2325.79,4188.07) | 480.1 (353.88,654.05) | 367.01 (225.08,534.08) | 3630.4 (2499.12,5005.38) | 563.99 (374.7,793.24) | 433.85 (248.11,709.1) |
| Vanuatu | 5566.83 (4217.92,7344.93) | 774.45 (559.2,1064.2) | 671.61 (426.07,981.82) | 6488.48 (4454.92,9299.02) | 892.29 (606.05,1296.11) | 781.31 (456.87,1221.68) |
| Venezuela (Bolivarian Republic of) | 6321.56 (4660.94,8349.57) | 881.78 (631.38,1186.78) | 757.96 (470.33,1115.19) | 7275.54 (5158.31,10104.79) | 991.62 (674.33,1369.01) | 867.69 (538.89,1345.32) |
| Viet Nam | 3451.34 (2600.86,4664.82) | 577.8 (427.25,785.52) | 417.43 (262.55,621.92) | 4297.38 (3009.34,5917.67) | 710.04 (482.05,1003.5) | 519.13 (315.57,832.97) |
| Yemen | 8289.27 (6144.54,11084.16) | 978.56 (698.78,1385.59) | 968.81 (606.15,1416.93) | 8733.06 (5989.61,12159.61) | 1021.75 (681.16,1501.91) | 1020.16 (609.92,1557.41) |
| Zambia | 5523.45 (4142.1,7581.93) | 817.71 (581.17,1137.81) | 653.02 (416.73,964.98) | 7132.94 (4894.5,9952.76) | 1038.89 (671.34,1545.26) | 845.96 (518.47,1336.13) |
| Zimbabwe | 4300.24 (3312.63,5682.06) | 642.83 (470.15,896.32) | 511.93 (322.18,746.22) | 5554.43 (3693.7,8047.8) | 824.66 (534.82,1205.56) | 659.58 (392.21,994.45) |

**Table S4.** Changes in prevalence, incidence, and disability-adjusted life years of anxiety disorders in women of child-bearing age in 204 countries from 1990 to 2021.

|  | Prevalence | | | Incidence | | | Disability-adjusted life years | | |
| --- | --- | --- | --- | --- | --- | --- | --- | --- | --- |
| location | number_1990 | number_2021 | Percentage change/% | number_1990 | number_2021 | Percentage change/% | number_1990 | number_2021 | Percentage change/% |
| Afghanistan | 178960.2007 | 748721.0374 | 318.3729 | 21431.4776 | 88105.8555 | 311.1049 | 21041.1473 | 88450.9136 | 320.3712 |
| Albania | 44994.013 | 45847.2148 | 1.8963 | 6343.7617 | 6368.5193 | 0.3903 | 5425.223 | 5500.04 | 1.3791 |
| Algeria | 474331.6306 | 1015208.202 | 114.0292 | 56614.9203 | 116037.1085 | 104.9585 | 56691.3723 | 120176.1611 | 111.9832 |
| American Samoa | 669.3349 | 753.2178 | 12.5323 | 92.922 | 104.1153 | 12.0458 | 80.9097 | 90.1278 | 11.393 |
| Andorra | 1276.5679 | 2051.5415 | 60.7076 | 142.8052 | 224.2744 | 57.0491 | 152.2863 | 243.1085 | 59.6391 |
| Angola | 122653.7355 | 472354.3065 | 285.112 | 17313.3415 | 67574.5996 | 290.3036 | 14495.7273 | 55899.8956 | 285.6302 |
| Antigua and Barbuda | 1124.7135 | 2090.5047 | 85.87 | 154.8073 | 280.3315 | 81.0842 | 134.6239 | 248.8744 | 84.8665 |
| Argentina | 703538.736 | 1311620.018 | 86.4318 | 82559.5909 | 151977.4 | 84.0821 | 83915.3023 | 155911.7073 | 85.7965 |
| Armenia | 33117.4798 | 39074.1668 | 17.9865 | 4861.4721 | 5697.7484 | 17.2021 | 3976.8452 | 4688.3322 | 17.8907 |
| Australia | 447618.1692 | 652910.1596 | 45.8632 | 51032.3931 | 73364.2964 | 43.7603 | 52880.2753 | 76901.6979 | 45.4261 |
| Austria | 178507.1671 | 219968.7815 | 23.2269 | 19476.367 | 23516.6214 | 20.7444 | 21314.9535 | 26215.1139 | 22.9893 |
| Azerbaijan | 64167.0452 | 125108.5183 | 94.9732 | 9766.6158 | 18927.9192 | 93.8022 | 7730.0312 | 15003.7081 | 94.0963 |
| Bahamas | 5014.5397 | 9595.4416 | 91.3524 | 692.1534 | 1294.7058 | 87.0548 | 600.2039 | 1139.7333 | 89.891 |
| Bahrain | 10060.4634 | 33171.9935 | 229.7263 | 1148.4249 | 3738.2024 | 225.5069 | 1197.1386 | 3925.2091 | 227.8826 |
| Bangladesh | 1049656.932 | 2514781.812 | 139.5813 | 169166.015 | 400827.1236 | 136.9431 | 124539.5363 | 297781.437 | 139.1059 |
| Barbados | 4737.4441 | 6362.4058 | 34.3004 | 648.5004 | 853.2752 | 31.5767 | 566.5081 | 756.4068 | 33.5209 |
| Belarus | 134333.4958 | 166243.2554 | 23.7541 | 18343.4313 | 22656.3048 | 23.5118 | 16138.2551 | 19828.2997 | 22.8652 |
| Belgium | 180971.6692 | 219180.6735 | 21.1133 | 21562.3985 | 25920.6896 | 20.2125 | 21578.2816 | 26036.4268 | 20.6603 |
| Belize | 2823.9368 | 10001.0532 | 254.1529 | 394.9225 | 1382.6932 | 250.1176 | 338.5453 | 1191.7908 | 252.0329 |
| Benin | 46527.2065 | 154176.977 | 231.3695 | 6351.1966 | 20959.9072 | 230.0151 | 5499.3168 | 18387.044 | 234.3514 |
| Bermuda | 1198.0448 | 1181.8878 | -1.3486 | 162.1023 | 156.5712 | -3.4121 | 143.6155 | 140.9393 | -1.8634 |
| Bhutan | 6296.6623 | 9955.1889 | 58.1026 | 1003.2868 | 1565.8279 | 56.0698 | 751.7666 | 1188.7542 | 58.1281 |
| Bolivia (Plurinational State of) | 132825.677 | 403645.8255 | 203.8914 | 17014.2705 | 51045.5041 | 200.0158 | 15843.9978 | 48095.2401 | 203.555 |
| Bosnia and Herzegovina | 61529.8971 | 53101.0779 | -13.6987 | 8706.5178 | 7449.6791 | -14.4356 | 7394.0212 | 6340.6227 | -14.2466 |
| Botswana | 16084.8491 | 46488.4553 | 189.0202 | 2313.744 | 6604.5613 | 185.4491 | 1918.271 | 5459.6473 | 184.6129 |
| Brazil | 3579704.822 | 9452216.693 | 164.0502 | 470357.887 | 1079906.284 | 129.5925 | 423384.6877 | 1112986.818 | 162.8784 |
| Brunei Darussalam | 3196.0455 | 6807.3611 | 112.9932 | 455.3603 | 962.9101 | 111.4611 | 386.838 | 821.0236 | 112.2397 |
| Bulgaria | 110952.6221 | 115491.2639 | 4.0906 | 15567.6609 | 16174.8651 | 3.9004 | 13303.7156 | 13768.5362 | 3.4939 |
| Burkina Faso | 87346.5159 | 249359.1039 | 185.4826 | 12011.2383 | 34332.2956 | 185.8348 | 10368.4467 | 29836.7949 | 187.7653 |
| Burundi | 68019.8666 | 207260.9665 | 204.7065 | 9986.8715 | 30494.3999 | 205.3449 | 8090.2007 | 24810.8758 | 206.6781 |
| Cabo Verde | 3331.2408 | 7519.8575 | 125.7374 | 456.1646 | 1035.3909 | 126.9775 | 400.2411 | 901.0939 | 125.1378 |
| Cambodia | 146312.6825 | 336624.4683 | 130.072 | 21271.7929 | 48357.0533 | 127.3295 | 17424.0684 | 40495.2915 | 132.4101 |
| Cameroon | 101903.9045 | 371503.3866 | 264.5625 | 13911.4143 | 50893.4425 | 265.8395 | 12067.3626 | 44357.1336 | 267.5794 |
| Canada | 587688.0414 | 741410.6789 | 26.1572 | 77266.8219 | 98779.7324 | 27.8424 | 70162.937 | 87853.7857 | 25.214 |
| Central African Republic | 35214.2902 | 84406.8483 | 139.6949 | 4898.1584 | 11746.4046 | 139.8127 | 4137.787 | 9984.3638 | 141.2972 |
| Chad | 57128.8586 | 181783.7973 | 218.1996 | 7785.6227 | 24387.2879 | 213.2349 | 6788.4948 | 21690.2016 | 219.5142 |
| Chile | 393732.8621 | 613530.7783 | 55.8241 | 41832.9031 | 64984.2587 | 55.3425 | 46825.7537 | 72569.6515 | 54.9781 |
| China | 16006097.41 | 15796801.12 | -1.3076 | 2219549.381 | 2225610.97 | 0.2731 | 1941978.021 | 1915987.351 | -1.3384 |
| Colombia | 623126.9461 | 1167940.967 | 87.4323 | 83188.317 | 153704.1609 | 84.7665 | 74749.7717 | 140138.1717 | 87.4764 |
| Comoros | 5575.9578 | 12673.4627 | 127.2876 | 825.5233 | 1845.9865 | 123.6141 | 664.5948 | 1512.4271 | 127.5713 |
| Congo | 29107.7652 | 85294.6051 | 193.0304 | 4164.0631 | 12146.4239 | 191.6964 | 3435.6261 | 10045.4659 | 192.3911 |
| Cook Islands | 254.3469 | 275.606 | 8.3583 | 35.2979 | 37.981 | 7.6014 | 30.7157 | 33.0836 | 7.709 |
| Costa Rica | 51182.8592 | 108156.3184 | 111.3136 | 6999.4541 | 14745.8866 | 110.672 | 6134.1708 | 12930.9521 | 110.802 |
| Cite d'Ivoire | 117427.5343 | 308752.1606 | 162.93 | 15954.7136 | 42181.4514 | 164.3824 | 13842.4751 | 36780.1438 | 165.705 |
| Croatia | 63394.9119 | 62347.5081 | -1.6522 | 8896.2626 | 8682.4261 | -2.4037 | 7607.5509 | 7475.0868 | -1.7412 |
| Cuba | 210545.0985 | 220978.0574 | 4.9552 | 28808.3766 | 29558.6092 | 2.6042 | 25102.1997 | 26307.9187 | 4.8032 |
| Cyprus | 18490.599 | 38433.8122 | 107.856 | 1991.6044 | 4028.1778 | 102.2579 | 2209.5355 | 4579.2506 | 107.2495 |
| Czechia | 129195.0969 | 151204.6569 | 17.0359 | 18404.3525 | 21449.3894 | 16.5452 | 15485.4025 | 18059.0855 | 16.6201 |
| Democratic People's Republic of Korea | 280922.934 | 339949.834 | 21.0118 | 38336.215 | 46866.4516 | 22.2511 | 34084.9498 | 41220.0291 | 20.9332 |
| Democratic Republic of the Congo | 448497.8375 | 1218065.434 | 171.5878 | 63943.5332 | 174449.5513 | 172.8181 | 52322.3028 | 143767.417 | 174.7727 |
| Denmark | 104465.1594 | 122154.9108 | 16.9336 | 12018.3122 | 14028.4519 | 16.7256 | 12406.1299 | 14509.245 | 16.9522 |
| Djibouti | 5075.2388 | 19593.3774 | 286.0582 | 761.7253 | 2854.6985 | 274.7675 | 608.0266 | 2341.0951 | 285.0317 |
| Dominica | 1173.0857 | 1438.1278 | 22.5936 | 162.7132 | 195.0162 | 19.8527 | 140.2063 | 170.7243 | 21.7665 |
| Dominican Republic | 132616.0984 | 242358.1277 | 82.7517 | 18172.5821 | 32736.5319 | 80.1424 | 15892.046 | 28883.4263 | 81.7477 |
| Ecuador | 208741.3773 | 522236.2358 | 150.1834 | 27550.9553 | 67186.1885 | 143.8616 | 25062.7905 | 62356.4967 | 148.8011 |
| Egypt | 949816.2584 | 2279547.344 | 139.9988 | 117573.1049 | 279205.6378 | 137.4741 | 113134.9056 | 271340.3133 | 139.8378 |
| El Salvador | 87480.0472 | 148941.9388 | 70.2582 | 12015.1966 | 20199.1498 | 68.1134 | 10449.7004 | 17817.8769 | 70.5109 |
| Equatorial Guinea | 5148.5177 | 21279.2205 | 313.3077 | 733.4302 | 3049.0559 | 315.7254 | 604.2079 | 2507.8427 | 315.0629 |
| Eritrea | 43209.1384 | 103958.3562 | 140.5934 | 6344.2701 | 15074.3483 | 137.6057 | 5120.9954 | 12445.6587 | 143.032 |
| Estonia | 19832.6044 | 19377.099 | -2.2968 | 2723.066 | 2668.863 | -1.9905 | 2373.1242 | 2314.0313 | -2.4901 |
| Eswatini | 9691.9695 | 23505.3985 | 142.5245 | 1397.4343 | 3364.4335 | 140.7579 | 1161.1217 | 2752.8457 | 137.085 |
| Ethiopia | 716462.2378 | 1857878.449 | 159.3128 | 104628.3056 | 282028.3487 | 169.5526 | 85205.7029 | 222268.8827 | 160.8615 |
| Fiji | 10885.811 | 15281.2913 | 40.3781 | 1519.4004 | 2109.6925 | 38.8503 | 1307.9146 | 1831.5419 | 40.0353 |
| Finland | 76996.7543 | 80451.3768 | 4.4867 | 9522.2664 | 10522.8743 | 10.5081 | 9172.2076 | 9574.2529 | 4.3833 |
| France | 1532429.12 | 1817183.747 | 18.5819 | 155233.9025 | 183734.6715 | 18.3599 | 182440.5714 | 215499.489 | 18.1204 |
| Gabon | 11745.4223 | 31479.3365 | 168.0137 | 1664.5288 | 4454.6287 | 167.621 | 1385.1499 | 3707.6434 | 167.6709 |
| Gambia | 9885.569 | 30923.4513 | 212.8141 | 1333.1982 | 4197.5155 | 214.8456 | 1174.5877 | 3667.4163 | 212.2301 |
| Georgia | 46979.8775 | 36196.9648 | -22.9522 | 7121.0288 | 5454.1672 | -23.4076 | 5661.3805 | 4337.7786 | -23.3795 |
| Germany | 1876788.411 | 2091825.684 | 11.4577 | 197172.53 | 214415.3664 | 8.7451 | 223133.9142 | 247896.8251 | 11.0978 |
| Ghana | 144664.9877 | 421641.7399 | 191.4608 | 19981.7465 | 58687.8663 | 193.7074 | 17134.422 | 50250.9212 | 193.2747 |
| Greece | 221470.15 | 241878.6971 | 9.215 | 24627.6612 | 26017.6087 | 5.6438 | 26392.4483 | 28623.5512 | 8.4536 |
| Greenland | 1255.5374 | 1400.0575 | 11.5106 | 164.607 | 183.1616 | 11.272 | 147.5645 | 164.8066 | 11.6845 |
| Grenada | 1331.3951 | 2218.4866 | 66.6287 | 184.9788 | 299.9677 | 62.1632 | 158.8806 | 264.0053 | 66.1659 |
| Guam | 1932.6483 | 2433.8778 | 25.9349 | 268.7932 | 335.5786 | 24.8464 | 233.7602 | 293.0752 | 25.3743 |
| Guatemala | 120559.9376 | 368717.3338 | 205.8374 | 16714.5104 | 50711.2621 | 203.3966 | 14273.6783 | 43754.4508 | 206.5394 |
| Guinea | 58801.2489 | 167888.6601 | 185.5189 | 8011.5121 | 22758.3255 | 184.0703 | 6975.2308 | 20004.7088 | 186.7964 |
| Guinea-Bissau | 9753.6148 | 25031.4913 | 156.6381 | 1341.961 | 3453.2479 | 157.3285 | 1158.3111 | 2970.7078 | 156.4689 |
| Guyana | 14219.4815 | 19014.8465 | 33.7239 | 1965.1987 | 2584.0443 | 31.4902 | 1688.4884 | 2244.344 | 32.9203 |
| Haiti | 108637.1189 | 305377.0145 | 181.0982 | 14858.4846 | 41191.9166 | 177.2282 | 12768.076 | 35766.9293 | 180.1278 |
| Honduras | 67474.4232 | 250908.1397 | 271.8567 | 9441.7924 | 34783.938 | 268.404 | 8087.8961 | 29995.2578 | 270.866 |
| Hungary | 132305.9896 | 145603.7345 | 10.0508 | 18622.65 | 20358.4329 | 9.3208 | 15836.1859 | 17374.8253 | 9.716 |
| Iceland | 5411.1699 | 7184.8121 | 32.7774 | 617.934 | 811.47 | 31.3199 | 646.6803 | 855.1693 | 32.2399 |
| India | 9175622.813 | 21266721.99 | 131.7742 | 1472012.356 | 3428094.323 | 132.8849 | 1079452.704 | 2510738.849 | 132.5937 |
| Indonesia | 2495087.19 | 5191008.372 | 108.0492 | 386805.9177 | 784415.9175 | 102.7932 | 301174.3664 | 626106.0135 | 107.8882 |
| Iran (Islamic Republic of) | 1415729.579 | 3087644.839 | 118.0957 | 158971.463 | 324117.7493 | 103.8842 | 168444.9229 | 364048.0751 | 116.1229 |
| Iraq | 364098.2358 | 1099504.3 | 201.9801 | 41499.1983 | 121772.9783 | 193.4345 | 43295.818 | 130475.9518 | 201.3592 |
| Ireland | 90420.0851 | 143809.316 | 59.0458 | 9441.9281 | 14626.0938 | 54.9058 | 10788.9331 | 17022.4787 | 57.7772 |
| Israel | 70363.1633 | 148559.2685 | 111.1322 | 9467.8829 | 19784.8578 | 108.9681 | 8425.7353 | 17731.3664 | 110.443 |
| Italy | 1390617.495 | 1345785.754 | -3.2239 | 159006.0234 | 156160.2431 | -1.7897 | 165427.9592 | 159930.7293 | -3.323 |
| Jamaica | 39199.5029 | 67018.6579 | 70.9681 | 5530.7199 | 9208.7118 | 66.5011 | 4702.0432 | 8003.79 | 70.2194 |
| Japan | 1327979.853 | 1229631.338 | -7.4059 | 196755.7214 | 181288.2624 | -7.8612 | 159767.4372 | 147802.4634 | -7.489 |
| Jordan | 70598.3431 | 304127.0788 | 330.785 | 8323.0112 | 34961.6893 | 320.0606 | 8436.5549 | 36164.662 | 328.6662 |
| Kazakhstan | 128540.2257 | 179936.6029 | 39.9847 | 20051.149 | 27800.9716 | 38.6503 | 15421.055 | 21501.2576 | 39.4279 |
| Kenya | 271833.3077 | 829366.3407 | 205.1011 | 42351.139 | 127243.9301 | 200.4498 | 32539.5813 | 99051.6985 | 204.4037 |
| Kiribati | 1038.1722 | 2067.0236 | 99.1022 | 144.0622 | 285.8552 | 98.4248 | 124.0409 | 246.8868 | 99.0365 |
| Kuwait | 32941.9063 | 121468.2491 | 268.7347 | 3893.9938 | 13770.3909 | 253.6316 | 3931.6611 | 14362.8057 | 265.3114 |
| Kyrgyzstan | 32104.823 | 68807.3639 | 114.321 | 5057.6703 | 10750.5801 | 112.5599 | 3864.3357 | 8253.8865 | 113.5913 |
| Lao People's Democratic Republic | 60772.6853 | 152457.013 | 150.8644 | 8640.109 | 21238.4688 | 145.8125 | 7283.9845 | 18372.0918 | 152.2258 |
| Latvia | 35403.6872 | 30888.4774 | -12.7535 | 4786.1276 | 4169.4593 | -12.8845 | 4239.3163 | 3681.6133 | -13.1555 |
| Lebanon | 76100.6123 | 204474.0279 | 168.6891 | 7985.6755 | 20775.7114 | 160.1622 | 9005.6338 | 24065.7584 | 167.23 |
| Lesotho | 18879.693 | 36672.1068 | 94.241 | 2714.1629 | 5227.5369 | 92.6022 | 2244.6497 | 4278.3075 | 90.6002 |
| Liberia | 24620.0065 | 67789.0397 | 175.3413 | 3310.142 | 9101.3079 | 174.9522 | 2858.4782 | 7926.8902 | 177.3116 |
| Libya | 79694.1968 | 204210.9335 | 156.2432 | 9248.1916 | 22685.2225 | 145.2936 | 9527 | 24134.0794 | 153.323 |
| Lithuania | 58497.0756 | 51936.0038 | -11.2161 | 7425.7036 | 6591.1816 | -11.2383 | 6999.208 | 6197.4286 | -11.4553 |
| Luxembourg | 8179.0175 | 15326.8799 | 87.3927 | 914.2156 | 1705.6912 | 86.5743 | 972.9488 | 1823.0734 | 87.3761 |
| Madagascar | 151030.5361 | 488860.19 | 223.683 | 22000.6341 | 70484.2332 | 220.3736 | 17900.6946 | 58231.2155 | 225.3014 |
| Malawi | 124896.6324 | 342761.7821 | 174.4364 | 18368.7974 | 50403.5483 | 174.3976 | 14645.6477 | 40840.9597 | 178.8607 |
| Malaysia | 294530.3439 | 710998.8928 | 141.4009 | 41264.2669 | 98135.2199 | 137.8213 | 35492.4797 | 85393.3806 | 140.5957 |
| Maldives | 2542.4238 | 7632.799 | 200.2174 | 380.9031 | 1122.8255 | 194.7798 | 303.6033 | 910.8067 | 199.999 |
| Mali | 73910.4856 | 241398.13 | 226.6088 | 10477.4899 | 33632.0066 | 220.993 | 8768.5723 | 28751.2654 | 227.8899 |
| Malta | 8736.789 | 9905.8435 | 13.3808 | 935.9312 | 1048.412 | 12.0181 | 1043.8089 | 1175.5829 | 12.6243 |
| Marshall Islands | 545.5205 | 947.5447 | 73.6955 | 76.0809 | 130.411 | 71.4109 | 65.7267 | 113.3792 | 72.5008 |
| Mauritania | 18473.7986 | 47443.5762 | 156.8155 | 2591.3486 | 6659.7984 | 157.0012 | 2202.9765 | 5689.1584 | 158.2487 |
| Mauritius | 16493.5444 | 21718.2056 | 31.677 | 2447.4635 | 3181.2095 | 29.9799 | 1973.6722 | 2592.7147 | 31.365 |
| Mexico | 968811.5178 | 2742597.598 | 183.0889 | 149784.388 | 401582.7313 | 168.1072 | 116163.6156 | 326469.8467 | 181.0431 |
| Micronesia (Federated States of) | 1294.3341 | 1679.5285 | 29.76 | 179.8009 | 231.6091 | 28.8142 | 155.8006 | 201.7526 | 29.4941 |
| Monaco | 585.4519 | 744.9971 | 27.2516 | 64.1619 | 81.9093 | 27.6602 | 69.6794 | 88.4071 | 26.877 |
| Mongolia | 15980.6855 | 29259.0031 | 83.0898 | 2493.4703 | 4520.7693 | 81.3043 | 1916.348 | 3498.1259 | 82.5413 |
| Montenegro | 8385.0481 | 11034.1633 | 31.5933 | 1179.3021 | 1539.8712 | 30.5748 | 1009.7401 | 1319.1373 | 30.6413 |
| Morocco | 511845.4278 | 962506.2523 | 88.0463 | 60615.0894 | 110219.8265 | 81.8356 | 60808.6138 | 113496.0364 | 86.6447 |
| Mozambique | 179529.8205 | 531590.4618 | 196.1015 | 25835.4567 | 77179.9534 | 198.7366 | 21029.3609 | 61879.6644 | 194.2537 |
| Myanmar | 569844.8508 | 1114835.849 | 95.6385 | 84829.0804 | 162851.0503 | 91.9755 | 68321.6239 | 133668.1508 | 95.6455 |
| Namibia | 16652.5688 | 47843.7817 | 187.3057 | 2409.5662 | 6812.8192 | 182.7405 | 1987.6218 | 5673.4661 | 185.4399 |
| Nauru | 135.0845 | 183.3229 | 35.7098 | 18.781 | 25.2419 | 34.4012 | 16.2671 | 22.0402 | 35.4893 |
| Nepal | 196701.8287 | 531333.1913 | 170.1211 | 31252.5934 | 84620.5121 | 170.7632 | 23204.8956 | 63058.1401 | 171.745 |
| Netherlands | 379384.2655 | 453694.9712 | 19.5872 | 38062.249 | 44264.4703 | 16.2949 | 45434.402 | 54072.8412 | 19.013 |
| New Zealand | 111129.4854 | 163424.0624 | 47.0573 | 11956.9523 | 17729.2955 | 48.276 | 13091.647 | 19312.2193 | 47.5156 |
| Nicaragua | 60503.0524 | 153572.3534 | 153.8258 | 8298.3494 | 20863.7971 | 151.421 | 7240.7144 | 18347.1165 | 153.3882 |
| Niger | 69712.2647 | 228160.2372 | 227.2885 | 9737.8631 | 31334.0022 | 221.7749 | 8271.6612 | 27433.8457 | 231.6607 |
| Nigeria | 860567.2408 | 2564443.216 | 197.9945 | 120997.2621 | 360093.7903 | 197.6049 | 101887.0053 | 305615.4952 | 199.9553 |
| Niue | 27.5547 | 24.5026 | -11.0764 | 3.8248 | 3.3739 | -11.7878 | 3.3178 | 2.9371 | -11.4732 |
| North Macedonia | 27128.154 | 41982.4642 | 54.7561 | 3827.4841 | 5866.4616 | 53.272 | 3256.3035 | 5023.9947 | 54.2852 |
| Northern Mariana Islands | 764.7141 | 786.9733 | 2.9108 | 106.668 | 108.2215 | 1.4563 | 92.6141 | 94.6588 | 2.2078 |
| Norway | 125769.9723 | 143211.7065 | 13.868 | 13121.2323 | 15596.8458 | 18.8672 | 15014.8424 | 17057.9483 | 13.6072 |
| Oman | 28548.7221 | 100674.399 | 252.6406 | 3324.0431 | 11349.5818 | 241.4391 | 3404.7762 | 11960.9108 | 251.298 |
| Pakistan | 1088637.066 | 3382200.643 | 210.6821 | 175556.5441 | 544860.093 | 210.3616 | 129126.3137 | 400488.9272 | 210.1528 |
| Palau | 226.9401 | 234.8039 | 3.4651 | 31.5819 | 32.1762 | 1.8819 | 27.3382 | 28.0884 | 2.7439 |
| Palestine | 39464.1131 | 140129.085 | 255.0798 | 4518.6565 | 15776.109 | 249.1327 | 4702.4159 | 16643.1141 | 253.9269 |
| Panama | 37233.2103 | 83213.7696 | 123.4934 | 5256.7387 | 11584.5769 | 120.3757 | 4470.8908 | 9926.1216 | 122.0166 |
| Papua New Guinea | 53626.97 | 161104.2641 | 200.4165 | 7445.5539 | 22289.5615 | 199.3674 | 6394.3567 | 19322.0733 | 202.1738 |
| Paraguay | 106054.7191 | 280796.7238 | 164.7659 | 12259.9418 | 31652.4877 | 158.1781 | 12645.6817 | 33290.5209 | 163.256 |
| Peru | 464751.4663 | 1196196.13 | 157.384 | 60043.9696 | 151282.3393 | 151.9526 | 55706.1136 | 143206.8938 | 157.0757 |
| Philippines | 933461.4017 | 2277418.343 | 143.9756 | 140588.2019 | 337880.8962 | 140.3337 | 112201.3237 | 273602.7805 | 143.8499 |
| Poland | 469789.5011 | 580544.083 | 23.5754 | 69328.2958 | 85301.1906 | 23.0395 | 56237.3844 | 69519.5347 | 23.618 |
| Portugal | 303867.0652 | 350291.1285 | 15.2778 | 29014.7264 | 31978.5775 | 10.215 | 36115.9876 | 41463.0354 | 14.8052 |
| Puerto Rico | 67131.5129 | 63309.6405 | -5.6931 | 9121.0609 | 8465.1115 | -7.1916 | 8032.5001 | 7556.6514 | -5.924 |
| Qatar | 6477.2948 | 49166.8365 | 659.0644 | 749.7512 | 5523.0014 | 636.6446 | 767.6901 | 5790.6245 | 654.2919 |
| Republic of Korea | 694542.6181 | 702024.6664 | 1.0773 | 93487.9777 | 92825.1971 | -0.7089 | 83777.5721 | 84573.1438 | 0.9496 |
| Republic of Moldova | 64701.463 | 65773.7906 | 1.6573 | 8597.7584 | 8717.592 | 1.3938 | 7761.9922 | 7862.3275 | 1.2926 |
| Romania | 280433.083 | 280518.6049 | 0.0305 | 40448.5367 | 40006.2636 | -1.0934 | 33517.1458 | 33501.1994 | -0.0476 |
| Russian Federation | 1870375.184 | 2291368.51 | 22.5085 | 268862.4743 | 327554.1204 | 21.8296 | 223610.2377 | 272624.6872 | 21.9196 |
| Rwanda | 87077.852 | 231114.1856 | 165.411 | 12867.2362 | 33719.4252 | 162.0565 | 10371.2013 | 27544.2386 | 165.5839 |
| Saint Kitts and Nevis | 686.0366 | 1308.0148 | 90.6625 | 95.5083 | 175.7022 | 83.9653 | 81.6954 | 155.4376 | 90.265 |
| Saint Lucia | 2342.4005 | 4190.3849 | 78.8928 | 325.4085 | 564.1731 | 73.3738 | 279.0057 | 496.3678 | 77.9059 |
| Saint Vincent and the Grenadines | 1799.1653 | 2415.4254 | 34.2526 | 250.5672 | 325.5084 | 29.9086 | 215.3374 | 286.76 | 33.1678 |
| Samoa | 2060.4232 | 3150.778 | 52.919 | 286.0464 | 435.4873 | 52.2436 | 249.0996 | 379.0617 | 52.1727 |
| San Marino | 537.6959 | 755.5944 | 40.5245 | 60.0848 | 82.9784 | 38.1022 | 64.165 | 89.7704 | 39.9056 |
| Sao Tome and Principe | 1083.8701 | 2575.2827 | 137.6007 | 148.7299 | 354.8632 | 138.5958 | 129.9348 | 307.9591 | 137.0104 |
| Saudi Arabia | 264345.3271 | 898408.229 | 239.8616 | 31406.9552 | 102545.3658 | 226.5053 | 31541.595 | 106285.398 | 236.969 |
| Senegal | 70194.858 | 193963.9662 | 176.3222 | 9712.0251 | 26911.982 | 177.0996 | 8330.8775 | 23084.8343 | 177.0997 |
| Serbia | 119272.5516 | 137293.5977 | 15.1091 | 16901.9892 | 19521.0187 | 15.4954 | 14309.0304 | 16486.7107 | 15.2189 |
| Seychelles | 970.8641 | 1738.0004 | 79.0158 | 144.6568 | 253.7439 | 75.4109 | 117.4932 | 209.2159 | 78.0663 |
| Sierra Leone | 45723.0006 | 107439.6866 | 134.9795 | 6091.7262 | 14320.7628 | 135.0855 | 5435.1182 | 12810.864 | 135.7053 |
| Singapore | 42933.1448 | 72618.2726 | 69.1427 | 6098.7934 | 10259.2056 | 68.217 | 5179.0905 | 8763.3413 | 69.2062 |
| Slovakia | 69414.7778 | 90743.7494 | 30.7268 | 9785.2456 | 12704.2678 | 29.8309 | 8311.6591 | 10855.5991 | 30.6069 |
| Slovenia | 25875.0493 | 27574.7039 | 6.5687 | 3664.4441 | 3878.8818 | 5.8518 | 3101.3692 | 3304.0419 | 6.5349 |
| Solomon Islands | 4159.8872 | 11096.4264 | 166.7483 | 578.2355 | 1532.8188 | 165.0856 | 501.3603 | 1330.5723 | 165.3924 |
| Somalia | 88736.2259 | 321358.0918 | 262.1498 | 13113.9466 | 48418.1036 | 269.2108 | 10449.2353 | 38107.7855 | 264.6945 |
| South Africa | 542662.2062 | 1170355.502 | 115.6692 | 78283.7167 | 165734.7508 | 111.7104 | 64413.084 | 136354.0899 | 111.6869 |
| South Sudan | 81932.549 | 163018.19 | 98.9663 | 11463.8813 | 22633.873 | 97.4364 | 9699.6654 | 19337.8906 | 99.3666 |
| Spain | 763340.8679 | 998130.5897 | 30.7582 | 90471.4858 | 111424.3888 | 23.1597 | 91300.9097 | 118577.365 | 29.8753 |
| Sri Lanka | 254420.4389 | 405329.2637 | 59.3147 | 37563.9179 | 58916.121 | 56.8423 | 30501.7612 | 48702.2957 | 59.6704 |
| Sudan | 382436.8915 | 1102628.395 | 188.3164 | 45210.8033 | 128892.099 | 185.0914 | 45339.3085 | 130936.3983 | 188.7922 |
| Suriname | 6597.8038 | 13512.488 | 104.8028 | 910.192 | 1844.787 | 102.6811 | 785.5181 | 1593.6027 | 102.8728 |
| Sweden | 164183.2144 | 205374.6054 | 25.0887 | 20122.4253 | 25112.1438 | 24.7968 | 19608.1563 | 24440.194 | 24.643 |
| Switzerland | 186157.4035 | 230758.0816 | 23.9586 | 18494.1259 | 22810.1328 | 23.3372 | 22103.5163 | 27345.3947 | 23.7151 |
| Syrian Arab Republic | 243690.1144 | 406676.2987 | 66.8826 | 28163.9336 | 45713.7935 | 62.3132 | 29039.1002 | 48056.6231 | 65.4894 |
| Taiwan (Province of China) | 294376.2259 | 292936.773 | -0.489 | 37619.8088 | 40439.6487 | 7.4956 | 35830.52 | 35387.566 | -1.2362 |
| Tajikistan | 39832.3032 | 110717.6498 | 177.9594 | 6140.1445 | 16842.018 | 174.2935 | 4806.2619 | 13322.6317 | 177.1932 |
| Thailand | 812886.9445 | 982726.6005 | 20.8934 | 123464.7369 | 145425.7717 | 17.7873 | 97727.0694 | 118001.0528 | 20.7455 |
| Timor-Leste | 10111.9292 | 22640.6445 | 123.9003 | 1508.2669 | 3384.6207 | 124.4046 | 1212.005 | 2743.9628 | 126.3986 |
| Togo | 38459.2722 | 104766.6471 | 172.4093 | 5130.5127 | 14103.3067 | 174.8908 | 4568.3099 | 12469.3313 | 172.9528 |
| Tokelau | 19.6582 | 20.5173 | 4.37 | 2.7273 | 2.8241 | 3.551 | 2.363 | 2.4664 | 4.3774 |
| Tonga | 1249.2414 | 1636.0146 | 30.9606 | 173.1493 | 224.8884 | 29.8812 | 151.1933 | 197.2855 | 30.4856 |
| Trinidad and Tobago | 20032.053 | 30924.6873 | 54.376 | 2828.1081 | 4266.4999 | 50.8606 | 2384.3481 | 3664.2366 | 53.6788 |
| Tunisia | 173548.3443 | 345343.1854 | 98.9896 | 20087.5078 | 38684.9025 | 92.5819 | 20751.9049 | 40694.8217 | 96.1016 |
| Türkiye | 945141.5082 | 1911521.07 | 102.2471 | 118948.6512 | 231634.5279 | 94.7349 | 112901.3595 | 226953.0062 | 101.0188 |
| Turkmenistan | 28668.2449 | 52421.2132 | 82.8546 | 4445.2637 | 8057.3983 | 81.2581 | 3442.2523 | 6292.4589 | 82.8006 |
| Tuvalu | 133.7895 | 186.9941 | 39.7673 | 18.625 | 25.8097 | 38.5753 | 16.0761 | 22.4864 | 39.8753 |
| Uganda | 203090.9148 | 676790.3754 | 233.245 | 30411.2025 | 100532.2153 | 230.5763 | 23985.3954 | 80909.9157 | 237.3299 |
| Ukraine | 587571.3928 | 622446.414 | 5.9355 | 85476.7163 | 90616.0056 | 6.0125 | 70245.8027 | 74028.6953 | 5.3852 |
| United Arab Emirates | 25625.0179 | 152116.5539 | 493.6252 | 3126.2085 | 17442.4255 | 457.9419 | 3043.2155 | 17874.3054 | 487.3493 |
| United Kingdom | 969085.2107 | 1297299.697 | 33.8685 | 125270.316 | 166816.7709 | 33.1654 | 115037.6608 | 153625.7838 | 33.5439 |
| United Republic of Tanzania | 331044.9707 | 975859.0617 | 194.7814 | 48845.8662 | 142117.7946 | 190.9515 | 38641.1943 | 115993.1764 | 200.1801 |
| United States of America | 6173590.138 | 9460586.694 | 53.2429 | 797032.226 | 1197771.841 | 50.279 | 732165.4264 | 1108375.73 | 51.3832 |
| United States Virgin Islands | 2006.8142 | 1505.9347 | -24.9589 | 271.6292 | 201.249 | -25.9104 | 239.5446 | 179.249 | -25.1709 |
| Uruguay | 70483.6475 | 101533.1116 | 44.052 | 7982.5712 | 11356.7653 | 42.2695 | 8424.9901 | 12057.0097 | 43.1101 |
| Uzbekistan | 150106.5797 | 323056.5091 | 115.2181 | 23518.6234 | 50187.3787 | 113.3942 | 17978.9283 | 38606.4117 | 114.7314 |
| Vanuatu | 1951.8211 | 5088.0375 | 160.6815 | 271.5358 | 699.7051 | 157.6843 | 235.4789 | 612.6719 | 160.1812 |
| Venezuela (Bolivarian Republic of) | 305995.8803 | 499375.4339 | 63.1968 | 42682.4814 | 68062.0603 | 59.4613 | 36689.0415 | 59556.0323 | 62.3265 |
| Viet Nam | 588950.0159 | 1097621.289 | 86.3692 | 98598.3058 | 181356.4231 | 83.9346 | 71231.19 | 132594.1125 | 86.1461 |
| Yemen | 227056.116 | 729704.2449 | 221.3762 | 26804.3336 | 85373.8734 | 218.5077 | 26537.1558 | 85241.2079 | 221.2146 |
| Zambia | 101579.66 | 350860.0117 | 245.4038 | 15038.1646 | 51101.4256 | 239.8116 | 12009.3652 | 41611.6726 | 246.4935 |
| Zimbabwe | 103396.5376 | 225544.8863 | 118.1358 | 15456.4732 | 33486.3051 | 116.6491 | 12308.9313 | 26783.2632 | 117.5921 |

| **Table S5.** Global age structure of depressive disorders prevalence, Incidence and DALYs among WCBA, 2021 | | | | | | | | | |
| --- | --- | --- | --- | --- | --- | --- | --- | --- | --- |
| measure | location | sex | age | cause | metric | year | val | upper | lower |
| Prevalence | Global | Female | 15-19 years | Depressive disorders | Number | 2021 | 12840700.853904 | 16836971.1966016 | 9489523.30927841 |
| Prevalence | Global | Female | 15-19 years | Depressive disorders | Rate | 2021 | 4228.78388586219 | 5544.86186486212 | 3125.15210122581 |
| Prevalence | Global | Female | 20-24 years | Depressive disorders | Number | 2021 | 16718165.7241053 | 23077956.2961346 | 12607116.8321669 |
| Prevalence | Global | Female | 20-24 years | Depressive disorders | Rate | 2021 | 5691.25107409572 | 7856.2711798539 | 4291.75475326612 |
| Prevalence | Global | Female | 25-29 years | Depressive disorders | Number | 2021 | 17500006.5582143 | 22147968.974316 | 14180521.715243 |
| Prevalence | Global | Female | 25-29 years | Depressive disorders | Rate | 2021 | 6014.01103040774 | 7611.318845 | 4873.24468872143 |
| Prevalence | Global | Female | 30-34 years | Depressive disorders | Number | 2021 | 18739692.025425 | 23575257.4578796 | 14685995.658456 |
| Prevalence | Global | Female | 30-34 years | Depressive disorders | Rate | 2021 | 6268.90487080887 | 7886.52482163311 | 4912.84006114207 |
| Prevalence | Global | Female | 35-39 years | Depressive disorders | Number | 2021 | 19275719.764511 | 23134151.9438464 | 15606826.5505954 |
| Prevalence | Global | Female | 35-39 years | Depressive disorders | Rate | 2021 | 6938.63573182674 | 8327.54653336567 | 5617.95282808369 |
| Prevalence | Global | Female | 40-44 years | Depressive disorders | Number | 2021 | 18420973.8574191 | 22726531.6311895 | 14521667.9541417 |
| Prevalence | Global | Female | 40-44 years | Depressive disorders | Rate | 2021 | 7425.12163293894 | 9160.60480637688 | 5853.39036400762 |
| Prevalence | Global | Female | 45-49 years | Depressive disorders | Number | 2021 | 17742682.0689092 | 21023647.9193641 | 14956261.8900103 |
| Prevalence | Global | Female | 45-49 years | Depressive disorders | Rate | 2021 | 7529.4890823864 | 8921.83757032848 | 6347.01169624619 |
| Incidence | Global | Female | 15-19 years | Depressive disorders | Number | 2021 | 16955625.1135598 | 22811613.9556356 | 11658045.7402826 |
| Incidence | Global | Female | 15-19 years | Depressive disorders | Rate | 2021 | 5583.93775158636 | 7512.47043316736 | 3839.30414142172 |
| Incidence | Global | Female | 20-24 years | Depressive disorders | Number | 2021 | 19524170.5960749 | 28189084.7860857 | 14284978.1693674 |
| Incidence | Global | Female | 20-24 years | Depressive disorders | Rate | 2021 | 6646.48016471832 | 9596.21777377558 | 4862.9376387042 |
| Incidence | Global | Female | 25-29 years | Depressive disorders | Number | 2021 | 19103089.3205336 | 26658737.3969761 | 13908914.3535559 |
| Incidence | Global | Female | 25-29 years | Depressive disorders | Rate | 2021 | 6564.92267625048 | 9161.4788959552 | 4779.90474260812 |
| Incidence | Global | Female | 30-34 years | Depressive disorders | Number | 2021 | 19955617.9855076 | 26940779.1801378 | 14600131.7423698 |
| Incidence | Global | Female | 30-34 years | Depressive disorders | Rate | 2021 | 6675.6631122657 | 9012.37766323012 | 4884.11639156153 |
| Incidence | Global | Female | 35-39 years | Depressive disorders | Number | 2021 | 20443426.773985 | 26638877.5731462 | 15402041.2739303 |
| Incidence | Global | Female | 35-39 years | Depressive disorders | Rate | 2021 | 7358.97249119165 | 9589.13441588307 | 5544.23675131043 |
| Incidence | Global | Female | 40-44 years | Depressive disorders | Number | 2021 | 19207159.4495037 | 24950926.3716806 | 14510511.5178869 |
| Incidence | Global | Female | 40-44 years | Depressive disorders | Rate | 2021 | 7742.01712893583 | 10057.2132938355 | 5848.89343041317 |
| Incidence | Global | Female | 45-49 years | Depressive disorders | Number | 2021 | 18059504.2593065 | 21686443.6135673 | 14667827.6667418 |
| Incidence | Global | Female | 45-49 years | Depressive disorders | Rate | 2021 | 7663.93939910796 | 9203.10919115637 | 6224.60842448314 |
| DALYs | Global | Female | 15-19 years | Depressive disorders | Number | 2021 | 2421174.54833323 | 3649932.64241374 | 1522832.81179283 |
| DALYs | Global | Female | 15-19 years | Depressive disorders | Rate | 2021 | 797.357093770964 | 1202.01977433568 | 501.509296777506 |
| DALYs | Global | Female | 20-24 years | Depressive disorders | Number | 2021 | 3040490.8633267 | 4552662.73016322 | 1921336.88793599 |
| DALYs | Global | Female | 20-24 years | Depressive disorders | Rate | 2021 | 1035.05355654753 | 1549.83190624063 | 654.067605718187 |
| DALYs | Global | Female | 25-29 years | Depressive disorders | Number | 2021 | 3068097.72786358 | 4465907.83856861 | 1977866.96164541 |
| DALYs | Global | Female | 25-29 years | Depressive disorders | Rate | 2021 | 1054.37523788124 | 1534.74336781487 | 679.709100933522 |
| DALYs | Global | Female | 30-34 years | Depressive disorders | Number | 2021 | 3205876.37187194 | 4741597.38184578 | 2042397.00767731 |
| DALYs | Global | Female | 30-34 years | Depressive disorders | Rate | 2021 | 1072.44740071353 | 1586.18524157915 | 683.23388304261 |
| DALYs | Global | Female | 35-39 years | Depressive disorders | Number | 2021 | 3271980.31141411 | 4667624.36860094 | 2112188.51058118 |
| DALYs | Global | Female | 35-39 years | Depressive disorders | Rate | 2021 | 1177.80709514208 | 1680.19382012121 | 760.319554907385 |
| DALYs | Global | Female | 40-44 years | Depressive disorders | Number | 2021 | 3097075.36721033 | 4452880.73388011 | 1951314.0610877 |
| DALYs | Global | Female | 40-44 years | Depressive disorders | Rate | 2021 | 1248.36838084182 | 1794.86607613386 | 786.535194055706 |
| DALYs | Global | Female | 45-49 years | Depressive disorders | Number | 2021 | 2937728.42583591 | 4062875.74967875 | 1940257.97647239 |
| DALYs | Global | Female | 45-49 years | Depressive disorders | Rate | 2021 | 1246.68829793824 | 1724.16878584013 | 823.390240219764 |

*DALYs , Disability-Adjusted Life Years.

| **Table S6.** Global age structure of anxiety disorders prevalence, incidence and DALYs among WCBA, 2021 | | | | | | | | | |
| --- | --- | --- | --- | --- | --- | --- | --- | --- | --- |
| measure | location | sex | age | cause | metric | year | val | upper | lower |
| Prevalence | Global | Female | 15-19 years | Anxiety disorders | Number | 2021 | 20118504.8878659 | 25916527.1977682 | 15320457.2289611 |
| Prevalence | Global | Female | 15-19 years | Anxiety disorders | Rate | 2021 | 6625.55807859824 | 8535.0008413373 | 5045.43353131491 |
| Prevalence | Global | Female | 20-24 years | Anxiety disorders | Number | 2021 | 20952070.6489405 | 27599626.7814156 | 15481127.1556768 |
| Prevalence | Global | Female | 20-24 years | Anxiety disorders | Rate | 2021 | 7132.57043584507 | 9395.55260765776 | 5270.13447569309 |
| Prevalence | Global | Female | 25-29 years | Anxiety disorders | Number | 2021 | 21174295.2525716 | 28273700.2071132 | 15686229.197972 |
| Prevalence | Global | Female | 25-29 years | Anxiety disorders | Rate | 2021 | 7276.70842787787 | 9716.47321104611 | 5390.69257535944 |
| Prevalence | Global | Female | 30-34 years | Anxiety disorders | Number | 2021 | 21580461.9705283 | 27959318.3615094 | 16510212.7189358 |
| Prevalence | Global | Female | 30-34 years | Anxiety disorders | Rate | 2021 | 7219.21486104476 | 9353.10499356974 | 5523.08718795393 |
| Prevalence | Global | Female | 35-39 years | Anxiety disorders | Number | 2021 | 20185134.6726784 | 25323664.2770351 | 15770645.9519756 |
| Prevalence | Global | Female | 35-39 years | Anxiety disorders | Rate | 2021 | 7265.99568797654 | 9115.70016373277 | 5676.92251460487 |
| Prevalence | Global | Female | 40-44 years | Anxiety disorders | Number | 2021 | 17956932.8234152 | 22779989.0497752 | 13369854.9670137 |
| Prevalence | Global | Female | 40-44 years | Anxiety disorders | Rate | 2021 | 7238.07608655127 | 9182.15240957388 | 5389.11786712334 |
| Prevalence | Global | Female | 45-49 years | Anxiety disorders | Number | 2021 | 16345634.1147269 | 20970878.9865136 | 12070269.3106836 |
| Prevalence | Global | Female | 45-49 years | Anxiety disorders | Rate | 2021 | 6936.62170879929 | 8899.44393771737 | 5122.27861849093 |
| Incidence | Global | Female | 15-19 years | Anxiety disorders | Number | 2021 | 3192421.07279279 | 4520961.66253883 | 2019397.90514321 |
| Incidence | Global | Female | 15-19 years | Anxiety disorders | Rate | 2021 | 1051.34906132546 | 1488.8727682907 | 665.04137255227 |
| Incidence | Global | Female | 20-24 years | Anxiety disorders | Number | 2021 | 3011450.7674766 | 4413157.42785262 | 1709648.71479249 |
| Incidence | Global | Female | 20-24 years | Anxiety disorders | Rate | 2021 | 1025.16763488446 | 1502.34106814752 | 582.00404547729 |
| Incidence | Global | Female | 25-29 years | Anxiety disorders | Number | 2021 | 2935231.16977224 | 4002457.12734362 | 2101594.6732701 |
| Incidence | Global | Female | 25-29 years | Anxiety disorders | Rate | 2021 | 1008.71462951086 | 1375.47498810965 | 722.22907485482 |
| Incidence | Global | Female | 30-34 years | Anxiety disorders | Number | 2021 | 2941780.49304002 | 3857624.57635234 | 2165892.14169065 |
| Incidence | Global | Female | 30-34 years | Anxiety disorders | Rate | 2021 | 984.1005944 | 1290.47379560116 | 724.54615466836 |
| Incidence | Global | Female | 35-39 years | Anxiety disorders | Number | 2021 | 2716105.80534366 | 3749065.61904148 | 1844673.02198977 |
| Incidence | Global | Female | 35-39 years | Anxiety disorders | Rate | 2021 | 977.71025012915 | 1349.54237678523 | 664.02263055725 |
| Incidence | Global | Female | 40-44 years | Anxiety disorders | Number | 2021 | 2277911.16933109 | 3199556.34918211 | 1478412.85576728 |
| Incidence | Global | Female | 40-44 years | Anxiety disorders | Rate | 2021 | 918.1798765 | 1289.67638996734 | 595.91829198278 |
| Incidence | Global | Female | 45-49 years | Anxiety disorders | Number | 2021 | 1887230.17067472 | 2552572.89991683 | 1340409.61449897 |
| Incidence | Global | Female | 45-49 years | Anxiety disorders | Rate | 2021 | 800.88674930076 | 1083.23925927737 | 568.83167488985 |
| DALYs | Global | Female | 15-19 years | Anxiety disorders | Number | 2021 | 2455779.47679212 | 3595952.16214067 | 1544590.42263017 |
| DALYs | Global | Female | 15-19 years | Anxiety disorders | Rate | 2021 | 808.753415942417 | 1184.24256826826 | 508.674655985749 |
| DALYs | Global | Female | 20-24 years | Anxiety disorders | Number | 2021 | 2528461.65155766 | 3751039.04834583 | 1586329.85025273 |
| DALYs | Global | Female | 20-24 years | Anxiety disorders | Rate | 2021 | 860.746946029419 | 1276.94062645234 | 540.023445939617 |
| DALYs | Global | Female | 25-29 years | Anxiety disorders | Number | 2021 | 2531173.9023413 | 3764463.17360254 | 1588356.15514603 |
| DALYs | Global | Female | 25-29 years | Anxiety disorders | Rate | 2021 | 869.857260791457 | 1293.68654659062 | 545.850734711896 |
| DALYs | Global | Female | 30-34 years | Anxiety disorders | Number | 2021 | 2562575.0974798 | 3709231.89545816 | 1658868.35089208 |
| DALYs | Global | Female | 30-34 years | Anxiety disorders | Rate | 2021 | 857.246719348914 | 1240.83265962157 | 554.933766831859 |
| DALYs | Global | Female | 35-39 years | Anxiety disorders | Number | 2021 | 2377055.24732317 | 3378348.39870975 | 1562147.80343733 |
| DALYs | Global | Female | 35-39 years | Anxiety disorders | Rate | 2021 | 855.663014253267 | 1216.09616658804 | 562.322688840973 |
| DALYs | Global | Female | 40-44 years | Anxiety disorders | Number | 2021 | 2096544.7898212 | 3034095.62119931 | 1282716.75757446 |
| DALYs | Global | Female | 40-44 years | Anxiety disorders | Rate | 2021 | 845.074760640688 | 1222.98252023732 | 517.037156630212 |
| DALYs | Global | Female | 45-49 years | Anxiety disorders | Number | 2021 | 1896754.06967004 | 2776861.87512118 | 1160323.34319163 |
| DALYs | Global | Female | 45-49 years | Anxiety disorders | Rate | 2021 | 804.928420860253 | 1178.42111417068 | 492.408188946182 |

*DALYs , Disability-Adjusted Life Years.

| **Table S7.** Temporal joinpoint analysis of depressive disorders age-standardized prevalence rates, 1990-2021 | | | | | | | |
| --- | --- | --- | --- | --- | --- | --- | --- |
| Location | Start.Obs | End.Obs | measure | val | lower | upper | P.Value |
| Global | 1990 | 2021 | AAPC | 0.479 | 0.329 | 0.629 | <0.001 |
| High-middle SDI | 1990 | 2021 | AAPC | 0.316 | 0.145 | 0.486 | <0.001 |
| High SDI | 1990 | 2021 | AAPC | 0.9 | 0.669 | 1.131 | <0.001 |
| Low-middle SDI | 1990 | 2021 | AAPC | 0.334 | 0.144 | 0.524 | 0.001 |
| Low SDI | 1990 | 2021 | AAPC | 0.182 | 0.077 | 0.287 | 0.001 |
| Middle SDI | 1990 | 2021 | AAPC | 0.476 | 0.313 | 0.639 | <0.001 |
| Andean Latin America | 1990 | 2021 | AAPC | 0.93 | 0.518 | 1.344 | <0.001 |
| Australasia | 1990 | 2021 | AAPC | 0.359 | 0.302 | 0.415 | <0.001 |
| Caribbean | 1990 | 2021 | AAPC | 0.228 | 0.155 | 0.3 | <0.001 |
| Central Asia | 1990 | 2021 | AAPC | 0.629 | 0.505 | 0.754 | <0.001 |
| Central Europe | 1990 | 2021 | AAPC | 0.476 | 0.409 | 0.543 | <0.001 |
| Central Latin America | 1990 | 2021 | AAPC | 1.524 | 1.312 | 1.737 | <0.001 |
| Central Sub-Saharan Africa | 1990 | 2021 | AAPC | 0.219 | 0.049 | 0.389 | 0.011 |
| East Asia | 1990 | 2021 | AAPC | -0.35 | -0.532 | -0.168 | <0.001 |
| Eastern Europe | 1990 | 2021 | AAPC | 0.739 | 0.708 | 0.769 | <0.001 |
| Eastern Sub-Saharan Africa | 1990 | 2021 | AAPC | 0.234 | 0.18 | 0.288 | <0.001 |
| High-income Asia Pacific | 1990 | 2021 | AAPC | 0.645 | 0.568 | 0.722 | <0.001 |
| High-income North America | 1990 | 2021 | AAPC | 1.338 | 1.198 | 1.479 | <0.001 |
| North Africa and Middle East | 1990 | 2021 | AAPC | 0.614 | 0.51 | 0.718 | <0.001 |
| Oceania | 1990 | 2021 | AAPC | 0.136 | 0.072 | 0.2 | <0.001 |
| South Asia | 1990 | 2021 | AAPC | 0.279 | 0.152 | 0.405 | <0.001 |
| Southeast Asia | 1990 | 2021 | AAPC | 0.526 | 0.494 | 0.558 | <0.001 |
| Southern Latin America | 1990 | 2021 | AAPC | 0.551 | 0.417 | 0.686 | <0.001 |
| Southern Sub-Saharan Africa | 1990 | 2021 | AAPC | 0.908 | 0.716 | 1.101 | <0.001 |
| Tropical Latin America | 1990 | 2021 | AAPC | 0.686 | 0.628 | 0.744 | <0.001 |
| Western Europe | 1990 | 2021 | AAPC | 0.714 | 0.564 | 0.863 | <0.001 |
| Western Sub-Saharan Africa | 1990 | 2021 | AAPC | 0.025 | -0.122 | 0.173 | 0.738 |
| Global | 1990 | 2005 | APC | 0.048 | -0.021 | 0.117 | 0.166 |
| Global | 2005 | 2010 | APC | -1.293 | -1.784 | -0.8 | <0.001 |
| Global | 2010 | 2019 | APC | 0.419 | 0.236 | 0.602 | <0.001 |
| Global | 2019 | 2021 | APC | 8.782 | 6.756 | 10.848 | <0.001 |
| High-middle SDI | 1990 | 2010 | APC | -0.51 | -0.567 | -0.453 | <0.001 |
| High-middle SDI | 2010 | 2019 | APC | 0.66 | 0.419 | 0.901 | <0.001 |
| High-middle SDI | 2019 | 2021 | APC | 7.291 | 4.656 | 9.992 | <0.001 |
| High SDI | 1990 | 1999 | APC | 0.985 | 0.65 | 1.322 | <0.001 |
| High SDI | 1999 | 2018 | APC | -0.157 | -0.275 | -0.038 | 0.012 |
| High SDI | 2018 | 2021 | APC | 7.581 | 5.283 | 9.929 | <0.001 |
| Low-middle SDI | 1990 | 2005 | APC | 0.238 | 0.147 | 0.329 | <0.001 |
| Low-middle SDI | 2005 | 2010 | APC | -2.586 | -3.198 | -1.971 | <0.001 |
| Low-middle SDI | 2010 | 2019 | APC | 0.132 | -0.096 | 0.361 | 0.241 |
| Low-middle SDI | 2019 | 2021 | APC | 9.788 | 7.195 | 12.444 | <0.001 |
| Low SDI | 1990 | 2005 | APC | 0.07 | 0.019 | 0.12 | 0.01 |
| Low SDI | 2005 | 2010 | APC | -1.571 | -1.929 | -1.212 | <0.001 |
| Low SDI | 2010 | 2019 | APC | 0.014 | -0.116 | 0.144 | 0.829 |
| Low SDI | 2019 | 2021 | APC | 6.386 | 5.019 | 7.771 | <0.001 |
| Middle SDI | 1990 | 2006 | APC | -0.144 | -0.198 | -0.089 | <0.001 |
| Middle SDI | 2006 | 2009 | APC | -1.514 | -2.838 | -0.171 | 0.029 |
| Middle SDI | 2009 | 2019 | APC | 0.557 | 0.427 | 0.688 | <0.001 |
| Middle SDI | 2019 | 2021 | APC | 8.351 | 6.689 | 10.04 | <0.001 |
| Andean Latin America | 1990 | 2018 | APC | -0.235 | -0.362 | -0.109 | 0.001 |
| Andean Latin America | 2018 | 2021 | APC | 12.483 | 7.784 | 17.387 | <0.001 |
| Australasia | 1990 | 2000 | APC | 0.556 | 0.52 | 0.592 | <0.001 |
| Australasia | 2000 | 2005 | APC | 1.507 | 1.382 | 1.632 | <0.001 |
| Australasia | 2005 | 2019 | APC | -0.759 | -0.785 | -0.732 | <0.001 |
| Australasia | 2019 | 2021 | APC | 4.465 | 3.593 | 5.345 | <0.001 |
| Caribbean | 1990 | 2010 | APC | -0.826 | -0.848 | -0.803 | <0.001 |
| Caribbean | 2010 | 2019 | APC | 0.249 | 0.154 | 0.345 | <0.001 |
| Caribbean | 2019 | 2021 | APC | 11.286 | 10.096 | 12.488 | <0.001 |
| Central Asia | 1990 | 2011 | APC | -0.036 | -0.075 | 0.003 | 0.067 |
| Central Asia | 2011 | 2019 | APC | 0.496 | 0.281 | 0.711 | <0.001 |
| Central Asia | 2019 | 2021 | APC | 8.461 | 6.541 | 10.416 | <0.001 |
| Central Europe | 1990 | 1998 | APC | -0.208 | -0.297 | -0.119 | <0.001 |
| Central Europe | 1998 | 2010 | APC | -0.847 | -0.902 | -0.792 | <0.001 |
| Central Europe | 2010 | 2019 | APC | 0.594 | 0.5 | 0.688 | <0.001 |
| Central Europe | 2019 | 2021 | APC | 11.218 | 10.232 | 12.213 | <0.001 |
| Central Latin America | 1990 | 2019 | APC | 0.79 | 0.74 | 0.839 | <0.001 |
| Central Latin America | 2019 | 2021 | APC | 12.796 | 9.117 | 16.6 | <0.001 |
| Central Sub-Saharan Africa | 1990 | 2018 | APC | -0.138 | -0.192 | -0.083 | <0.001 |
| Central Sub-Saharan Africa | 2018 | 2021 | APC | 3.608 | 1.8 | 5.447 | <0.001 |
| East Asia | 1990 | 1996 | APC | -0.228 | -0.535 | 0.081 | 0.14 |
| East Asia | 1996 | 1999 | APC | -2.481 | -4.186 | -0.747 | 0.007 |
| East Asia | 1999 | 2009 | APC | -0.996 | -1.159 | -0.834 | <0.001 |
| East Asia | 2009 | 2021 | APC | 0.673 | 0.557 | 0.79 | <0.001 |
| Eastern Europe | 1990 | 1995 | APC | 0.873 | 0.811 | 0.935 | <0.001 |
| Eastern Europe | 1995 | 2001 | APC | -0.981 | -1.04 | -0.923 | <0.001 |
| Eastern Europe | 2001 | 2010 | APC | -0.438 | -0.466 | -0.41 | <0.001 |
| Eastern Europe | 2010 | 2015 | APC | 1.062 | 0.977 | 1.147 | <0.001 |
| Eastern Europe | 2015 | 2019 | APC | 0.022 | -0.115 | 0.159 | 0.736 |
| Eastern Europe | 2019 | 2021 | APC | 12.171 | 11.87 | 12.472 | <0.001 |
| Eastern Sub-Saharan Africa | 1990 | 2002 | APC | -0.037 | -0.074 | 0.001 | 0.053 |
| Eastern Sub-Saharan Africa | 2002 | 2013 | APC | -0.635 | -0.683 | -0.587 | <0.001 |
| Eastern Sub-Saharan Africa | 2013 | 2019 | APC | 0.275 | 0.127 | 0.424 | 0.001 |
| Eastern Sub-Saharan Africa | 2019 | 2021 | APC | 6.739 | 6.02 | 7.463 | <0.001 |
| High-income Asia Pacific | 1990 | 1995 | APC | -0.495 | -0.64 | -0.35 | <0.001 |
| High-income Asia Pacific | 1995 | 2000 | APC | 2.372 | 2.169 | 2.576 | <0.001 |
| High-income Asia Pacific | 2000 | 2005 | APC | 0.223 | 0.04 | 0.405 | 0.02 |
| High-income Asia Pacific | 2005 | 2015 | APC | -0.84 | -0.892 | -0.787 | <0.001 |
| High-income Asia Pacific | 2015 | 2019 | APC | 0.044 | -0.263 | 0.352 | 0.763 |
| High-income Asia Pacific | 2019 | 2021 | APC | 9.327 | 8.476 | 10.184 | <0.001 |
| High-income North America | 1990 | 1997 | APC | 2.7 | 2.437 | 2.963 | <0.001 |
| High-income North America | 1997 | 2019 | APC | -0.175 | -0.222 | -0.128 | <0.001 |
| High-income North America | 2019 | 2021 | APC | 14.122 | 11.843 | 16.448 | <0.001 |
| North Africa and Middle East | 1990 | 2001 | APC | -0.251 | -0.339 | -0.162 | <0.001 |
| North Africa and Middle East | 2001 | 2019 | APC | 0.372 | 0.325 | 0.419 | <0.001 |
| North Africa and Middle East | 2019 | 2021 | APC | 7.811 | 6.141 | 9.507 | <0.001 |
| Oceania | 1990 | 2006 | APC | 0.009 | -0.018 | 0.035 | 0.501 |
| Oceania | 2006 | 2011 | APC | -0.333 | -0.551 | -0.114 | 0.005 |
| Oceania | 2011 | 2019 | APC | 0.071 | -0.021 | 0.163 | 0.124 |
| Oceania | 2019 | 2021 | APC | 2.62 | 1.803 | 3.444 | <0.001 |
| South Asia | 1990 | 1994 | APC | 1.713 | 1.312 | 2.116 | <0.001 |
| South Asia | 1994 | 2006 | APC | -0.173 | -0.256 | -0.09 | <0.001 |
| South Asia | 2006 | 2010 | APC | -4.162 | -4.716 | -3.606 | <0.001 |
| South Asia | 2010 | 2019 | APC | 0.094 | -0.034 | 0.221 | 0.14 |
| South Asia | 2019 | 2021 | APC | 10.562 | 9.068 | 12.077 | <0.001 |
| Southeast Asia | 1990 | 2000 | APC | -0.108 | -0.126 | -0.09 | <0.001 |
| Southeast Asia | 2000 | 2005 | APC | 0.288 | 0.216 | 0.361 | <0.001 |
| Southeast Asia | 2005 | 2010 | APC | -0.28 | -0.352 | -0.207 | <0.001 |
| Southeast Asia | 2010 | 2013 | APC | 0.316 | 0.083 | 0.551 | 0.011 |
| Southeast Asia | 2013 | 2019 | APC | 0.44 | 0.387 | 0.493 | <0.001 |
| Southeast Asia | 2019 | 2021 | APC | 7.101 | 6.842 | 7.362 | <0.001 |
| Southern Latin America | 1990 | 2006 | APC | -0.093 | -0.135 | -0.05 | <0.001 |
| Southern Latin America | 2006 | 2009 | APC | -2.908 | -3.906 | -1.899 | <0.001 |
| Southern Latin America | 2009 | 2019 | APC | -0.049 | -0.141 | 0.043 | 0.279 |
| Southern Latin America | 2019 | 2021 | APC | 14.952 | 13.285 | 16.643 | <0.001 |
| Southern Sub-Saharan Africa | 1990 | 2006 | APC | -0.288 | -0.387 | -0.188 | <0.001 |
| Southern Sub-Saharan Africa | 2006 | 2019 | APC | 0.91 | 0.757 | 1.063 | <0.001 |
| Southern Sub-Saharan Africa | 2019 | 2021 | APC | 11 | 7.882 | 14.209 | <0.001 |
| Tropical Latin America | 1990 | 1995 | APC | 0.211 | 0.087 | 0.335 | 0.002 |
| Tropical Latin America | 1995 | 2000 | APC | 2.738 | 2.569 | 2.907 | <0.001 |
| Tropical Latin America | 2000 | 2006 | APC | 0.091 | -0.024 | 0.207 | 0.113 |
| Tropical Latin America | 2006 | 2010 | APC | -4.591 | -4.804 | -4.377 | <0.001 |
| Tropical Latin America | 2010 | 2019 | APC | -0.414 | -0.463 | -0.366 | <0.001 |
| Tropical Latin America | 2019 | 2021 | APC | 15.388 | 14.745 | 16.034 | <0.001 |
| Western Europe | 1990 | 2019 | APC | -0.006 | -0.034 | 0.021 | 0.638 |
| Western Europe | 2019 | 2021 | APC | 11.756 | 9.131 | 14.444 | <0.001 |
| Western Sub-Saharan Africa | 1990 | 2004 | APC | 0.185 | 0.038 | 0.332 | 0.016 |
| Western Sub-Saharan Africa | 2004 | 2016 | APC | -0.679 | -0.88 | -0.478 | <0.001 |
| Western Sub-Saharan Africa | 2016 | 2021 | APC | 1.282 | 0.551 | 2.02 | 0.001 |

| **Table S8**. Temporal joinpoint analysis of depressive disorders age-standardized incidence rates, 1990-2021 | | | | | | | |
| --- | --- | --- | --- | --- | --- | --- | --- |
| Location | Start.Obs | End.Obs | measure | val | lower | upper | P.Value |
| Global | 1990 | 2021 | AAPC | 0.584 | 0.422 | 0.746 | <0.001 |
| High-middle SDI | 1990 | 2021 | AAPC | 0.287 | 0.077 | 0.498 | 0.007 |
| High SDI | 1990 | 2021 | AAPC | 1.335 | 1.167 | 1.503 | <0.001 |
| Low-middle SDI | 1990 | 2021 | AAPC | 0.356 | 0.081 | 0.633 | 0.011 |
| Low SDI | 1990 | 2021 | AAPC | 0.242 | 0.095 | 0.39 | 0.001 |
| Middle SDI | 1990 | 2021 | AAPC | 0.54 | 0.354 | 0.726 | <0.001 |
| Andean Latin America | 1990 | 2021 | AAPC | 1.161 | 0.598 | 1.728 | <0.001 |
| Australasia | 1990 | 2021 | AAPC | 0.427 | 0.348 | 0.507 | <0.001 |
| Caribbean | 1990 | 2021 | AAPC | 0.263 | 0.188 | 0.338 | <0.001 |
| Central Asia | 1990 | 2021 | AAPC | 0.807 | 0.644 | 0.97 | <0.001 |
| Central Europe | 1990 | 2021 | AAPC | 0.638 | 0.519 | 0.756 | <0.001 |
| Central Latin America | 1990 | 2021 | AAPC | 1.829 | 1.544 | 2.116 | <0.001 |
| Central Sub-Saharan Africa | 1990 | 2021 | AAPC | 0.273 | 0.057 | 0.491 | 0.013 |
| East Asia | 1990 | 2021 | AAPC | -0.915 | -1.301 | -0.528 | <0.001 |
| Eastern Europe | 1990 | 2021 | AAPC | 0.93 | 0.88 | 0.98 | <0.001 |
| Eastern Sub-Saharan Africa | 1990 | 2021 | AAPC | 0.35 | 0.273 | 0.426 | <0.001 |
| High-income Asia Pacific | 1990 | 2021 | AAPC | 0.812 | 0.661 | 0.963 | <0.001 |
| High-income North America | 1990 | 2021 | AAPC | 1.947 | 1.485 | 2.41 | <0.001 |
| North Africa and Middle East | 1990 | 2021 | AAPC | 0.73 | 0.557 | 0.903 | <0.001 |
| Oceania | 1990 | 2021 | AAPC | 0.083 | -0.017 | 0.182 | 0.102 |
| South Asia | 1990 | 2021 | AAPC | 0.299 | 0.153 | 0.446 | <0.001 |
| Southeast Asia | 1990 | 2021 | AAPC | 0.62 | 0.554 | 0.686 | <0.001 |
| Southern Latin America | 1990 | 2021 | AAPC | 0.624 | 0.466 | 0.782 | <0.001 |
| Southern Sub-Saharan Africa | 1990 | 2021 | AAPC | 1.178 | 0.854 | 1.502 | <0.001 |
| Tropical Latin America | 1990 | 2021 | AAPC | 0.761 | 0.691 | 0.831 | <0.001 |
| Western Europe | 1990 | 2021 | AAPC | 0.889 | 0.699 | 1.081 | <0.001 |
| Western Sub-Saharan Africa | 1990 | 2021 | AAPC | 0.005 | -0.213 | 0.224 | 0.962 |
| Global | 1990 | 2005 | APC | 0.052 | -0.022 | 0.127 | 0.161 |
| Global | 2005 | 2010 | APC | -1.992 | -2.518 | -1.464 | <0.001 |
| Global | 2010 | 2019 | APC | 0.462 | 0.265 | 0.658 | <0.001 |
| Global | 2019 | 2021 | APC | 12.29 | 10.035 | 14.59 | <0.001 |
| High-middle SDI | 1990 | 2010 | APC | -0.897 | -0.967 | -0.828 | <0.001 |
| High-middle SDI | 2010 | 2019 | APC | 0.823 | 0.53 | 1.117 | <0.001 |
| High-middle SDI | 2019 | 2021 | APC | 10.264 | 6.914 | 13.719 | <0.001 |
| High SDI | 1990 | 2000 | APC | 1.64 | 1.478 | 1.803 | <0.001 |
| High SDI | 2000 | 2019 | APC | -0.225 | -0.292 | -0.157 | <0.001 |
| High SDI | 2019 | 2021 | APC | 15.664 | 12.808 | 18.593 | <0.001 |
| Low-middle SDI | 1990 | 2005 | APC | 0.257 | 0.13 | 0.384 | <0.001 |
| Low-middle SDI | 2005 | 2010 | APC | -3.677 | -4.551 | -2.795 | <0.001 |
| Low-middle SDI | 2010 | 2019 | APC | 0.075 | -0.254 | 0.406 | 0.64 |
| Low-middle SDI | 2019 | 2021 | APC | 13.448 | 9.556 | 17.478 | <0.001 |
| Low SDI | 1990 | 2005 | APC | 0.118 | 0.046 | 0.189 | 0.003 |
| Low SDI | 2005 | 2010 | APC | -2.304 | -2.791 | -1.815 | <0.001 |
| Low SDI | 2010 | 2019 | APC | 0.002 | -0.182 | 0.187 | 0.979 |
| Low SDI | 2019 | 2021 | APC | 9.071 | 7.098 | 11.08 | <0.001 |
| Middle SDI | 1990 | 2006 | APC | -0.376 | -0.438 | -0.315 | <0.001 |
| Middle SDI | 2006 | 2009 | APC | -2.537 | -4.027 | -1.024 | 0.002 |
| Middle SDI | 2009 | 2019 | APC | 0.764 | 0.616 | 0.913 | <0.001 |
| Middle SDI | 2019 | 2021 | APC | 12.086 | 10.113 | 14.096 | <0.001 |
| Andean Latin America | 1990 | 2018 | APC | -0.417 | -0.585 | -0.248 | <0.001 |
| Andean Latin America | 2018 | 2021 | APC | 17.152 | 10.518 | 24.184 | <0.001 |
| Australasia | 1990 | 2000 | APC | 0.649 | 0.6 | 0.698 | <0.001 |
| Australasia | 2000 | 2005 | APC | 1.874 | 1.699 | 2.05 | <0.001 |
| Australasia | 2005 | 2019 | APC | -0.954 | -0.99 | -0.918 | <0.001 |
| Australasia | 2019 | 2021 | APC | 5.596 | 4.357 | 6.85 | <0.001 |
| Caribbean | 1990 | 2011 | APC | -1.026 | -1.047 | -1.004 | <0.001 |
| Caribbean | 2011 | 2019 | APC | 0.462 | 0.338 | 0.586 | <0.001 |
| Caribbean | 2019 | 2021 | APC | 13.941 | 12.706 | 15.19 | <0.001 |
| Central Asia | 1990 | 2011 | APC | -0.105 | -0.155 | -0.055 | <0.001 |
| Central Asia | 2011 | 2019 | APC | 0.432 | 0.148 | 0.717 | 0.004 |
| Central Asia | 2019 | 2021 | APC | 12.567 | 9.975 | 15.22 | <0.001 |
| Central Europe | 1990 | 1998 | APC | -0.361 | -0.506 | -0.215 | <0.001 |
| Central Europe | 1998 | 2011 | APC | -1.393 | -1.469 | -1.316 | <0.001 |
| Central Europe | 2011 | 2019 | APC | 0.874 | 0.68 | 1.069 | <0.001 |
| Central Europe | 2019 | 2021 | APC | 18.451 | 16.574 | 20.358 | <0.001 |
| Central Latin America | 1990 | 2019 | APC | 0.93 | 0.862 | 0.997 | <0.001 |
| Central Latin America | 2019 | 2021 | APC | 15.813 | 10.773 | 21.083 | <0.001 |
| Central Sub-Saharan Africa | 1990 | 2018 | APC | -0.179 | -0.247 | -0.111 | <0.001 |
| Central Sub-Saharan Africa | 2018 | 2021 | APC | 4.595 | 2.263 | 6.981 | <0.001 |
| East Asia | 1990 | 1995 | APC | -0.963 | -2.125 | 0.213 | 0.103 |
| East Asia | 1995 | 1999 | APC | -3.463 | -5.808 | -1.059 | 0.007 |
| East Asia | 1999 | 2009 | APC | -1.967 | -2.4 | -1.532 | <0.001 |
| East Asia | 2009 | 2021 | APC | 0.863 | 0.554 | 1.172 | <0.001 |
| Eastern Europe | 1990 | 1995 | APC | 1.089 | 0.995 | 1.184 | <0.001 |
| Eastern Europe | 1995 | 2000 | APC | -1.349 | -1.476 | -1.222 | <0.001 |
| Eastern Europe | 2000 | 2010 | APC | -0.765 | -0.801 | -0.729 | <0.001 |
| Eastern Europe | 2010 | 2015 | APC | 1.162 | 1.028 | 1.295 | <0.001 |
| Eastern Europe | 2015 | 2019 | APC | -0.142 | -0.358 | 0.074 | 0.181 |
| Eastern Europe | 2019 | 2021 | APC | 17.67 | 17.139 | 18.204 | <0.001 |
| Eastern Sub-Saharan Africa | 1990 | 2002 | APC | 0.024 | -0.027 | 0.074 | 0.337 |
| Eastern Sub-Saharan Africa | 2002 | 2013 | APC | -0.953 | -1.018 | -0.888 | <0.001 |
| Eastern Sub-Saharan Africa | 2013 | 2019 | APC | 0.351 | 0.145 | 0.558 | 0.002 |
| Eastern Sub-Saharan Africa | 2019 | 2021 | APC | 9.944 | 8.872 | 11.027 | <0.001 |
| High-income Asia Pacific | 1990 | 1995 | APC | -0.804 | -1.056 | -0.552 | <0.001 |
| High-income Asia Pacific | 1995 | 2000 | APC | 3.093 | 2.756 | 3.432 | <0.001 |
| High-income Asia Pacific | 2000 | 2007 | APC | 0.064 | -0.106 | 0.235 | 0.436 |
| High-income Asia Pacific | 2007 | 2016 | APC | -0.897 | -1.009 | -0.785 | <0.001 |
| High-income Asia Pacific | 2016 | 2019 | APC | 0.207 | -0.903 | 1.33 | 0.698 |
| High-income Asia Pacific | 2019 | 2021 | APC | 11.021 | 9.501 | 12.563 | <0.001 |
| High-income North America | 1990 | 1999 | APC | 4.037 | 3.43 | 4.648 | <0.001 |
| High-income North America | 1999 | 2019 | APC | -0.407 | -0.582 | -0.231 | <0.001 |
| High-income North America | 2019 | 2021 | APC | 17.521 | 9.911 | 25.659 | <0.001 |
| North Africa and Middle East | 1990 | 2008 | APC | -0.104 | -0.171 | -0.036 | 0.004 |
| North Africa and Middle East | 2008 | 2019 | APC | 0.581 | 0.408 | 0.754 | <0.001 |
| North Africa and Middle East | 2019 | 2021 | APC | 9.432 | 6.657 | 12.28 | <0.001 |
| Oceania | 1990 | 2006 | APC | -0.124 | -0.162 | -0.086 | <0.001 |
| Oceania | 2006 | 2010 | APC | -0.689 | -1.153 | -0.222 | 0.006 |
| Oceania | 2010 | 2019 | APC | -0.084 | -0.186 | 0.019 | 0.105 |
| Oceania | 2019 | 2021 | APC | 4.113 | 2.859 | 5.381 | <0.001 |
| South Asia | 1990 | 1994 | APC | 2.353 | 1.888 | 2.82 | <0.001 |
| South Asia | 1994 | 2006 | APC | -0.276 | -0.37 | -0.182 | <0.001 |
| South Asia | 2006 | 2010 | APC | -5.925 | -6.562 | -5.283 | <0.001 |
| South Asia | 2010 | 2019 | APC | 0.046 | -0.107 | 0.199 | 0.538 |
| South Asia | 2019 | 2021 | APC | 14.617 | 12.848 | 16.414 | <0.001 |
| Southeast Asia | 1990 | 2000 | APC | -0.601 | -0.639 | -0.562 | <0.001 |
| Southeast Asia | 2000 | 2005 | APC | 0.028 | -0.123 | 0.179 | 0.698 |
| Southeast Asia | 2005 | 2010 | APC | -0.781 | -0.929 | -0.632 | <0.001 |
| Southeast Asia | 2010 | 2013 | APC | 0.284 | -0.186 | 0.756 | 0.217 |
| Southeast Asia | 2013 | 2019 | APC | 0.603 | 0.494 | 0.712 | <0.001 |
| Southeast Asia | 2019 | 2021 | APC | 13.034 | 12.428 | 13.644 | <0.001 |
| Southern Latin America | 1990 | 2006 | APC | -0.125 | -0.175 | -0.076 | <0.001 |
| Southern Latin America | 2006 | 2009 | APC | -2.792 | -3.95 | -1.619 | <0.001 |
| Southern Latin America | 2009 | 2019 | APC | -0.068 | -0.179 | 0.043 | 0.218 |
| Southern Latin America | 2019 | 2021 | APC | 16.45 | 14.46 | 18.474 | <0.001 |
| Southern Sub-Saharan Africa | 1990 | 2006 | APC | -0.538 | -0.666 | -0.411 | <0.001 |
| Southern Sub-Saharan Africa | 2006 | 2014 | APC | 1.442 | 1.005 | 1.88 | <0.001 |
| Southern Sub-Saharan Africa | 2014 | 2019 | APC | 0.38 | -0.743 | 1.516 | 0.491 |
| Southern Sub-Saharan Africa | 2019 | 2021 | APC | 17.105 | 12.54 | 21.855 | <0.001 |
| Tropical Latin America | 1990 | 1995 | APC | 0.121 | -0.021 | 0.264 | 0.09 |
| Tropical Latin America | 1995 | 2000 | APC | 3.202 | 3.003 | 3.402 | <0.001 |
| Tropical Latin America | 2000 | 2006 | APC | 0.015 | -0.124 | 0.155 | 0.817 |
| Tropical Latin America | 2006 | 2010 | APC | -5.462 | -5.721 | -5.201 | <0.001 |
| Tropical Latin America | 2010 | 2019 | APC | -0.614 | -0.673 | -0.555 | <0.001 |
| Tropical Latin America | 2019 | 2021 | APC | 19.159 | 18.346 | 19.978 | <0.001 |
| Western Europe | 1990 | 2019 | APC | -0.02 | -0.056 | 0.015 | 0.247 |
| Western Europe | 2019 | 2021 | APC | 15.051 | 11.619 | 18.589 | <0.001 |
| Western Sub-Saharan Africa | 1990 | 2004 | APC | 0.202 | -0.011 | 0.416 | 0.063 |
| Western Sub-Saharan Africa | 2004 | 2016 | APC | -1.029 | -1.324 | -0.733 | <0.001 |
| Western Sub-Saharan Africa | 2016 | 2021 | APC | 1.969 | 0.877 | 3.072 | 0.001 |

| **Table S9.** Temporal Joinpoint analysis of depressive disorders age-standardized DALYs rates, 1990-2021 | | | | | | | |
| --- | --- | --- | --- | --- | --- | --- | --- |
| location | Start.Obs | End.Obs | measure | val | lower | upper | P.Value |
| Global | 1990 | 2021 | AAPC | 0.524 | 0.376 | 0.673 | <0.001 |
| High-middle SDI | 1990 | 2021 | AAPC | 0.291 | 0.123 | 0.46 | 0.001 |
| High SDI | 1990 | 2021 | AAPC | 1.126 | 1.056 | 1.197 | <0.001 |
| Low-middle SDI | 1990 | 2021 | AAPC | 0.36 | 0.142 | 0.578 | 0.001 |
| Low SDI | 1990 | 2021 | AAPC | 0.235 | 0.115 | 0.355 | <0.001 |
| Middle SDI | 1990 | 2021 | AAPC | 0.455 | 0.203 | 0.708 | <0.001 |
| Andean Latin America | 1990 | 2021 | AAPC | 1.072 | 0.565 | 1.581 | <0.001 |
| Australasia | 1990 | 2021 | AAPC | 0.406 | 0.355 | 0.456 | <0.001 |
| Caribbean | 1990 | 2021 | AAPC | 0.223 | 0.16 | 0.285 | <0.001 |
| Central Asia | 1990 | 2021 | AAPC | 0.72 | 0.6 | 0.841 | <0.001 |
| Central Europe | 1990 | 2021 | AAPC | 0.546 | 0.454 | 0.638 | <0.001 |
| Central Latin America | 1990 | 2021 | AAPC | 1.665 | 1.438 | 1.894 | <0.001 |
| Central Sub-Saharan Africa | 1990 | 2021 | AAPC | 0.287 | 0.089 | 0.485 | 0.005 |
| East Asia | 1990 | 2021 | AAPC | -0.611 | -0.853 | -0.368 | <0.001 |
| Eastern Europe | 1990 | 2021 | AAPC | 0.834 | 0.794 | 0.873 | <0.001 |
| Eastern Sub-Saharan Africa | 1990 | 2021 | AAPC | 0.313 | 0.251 | 0.374 | <0.001 |
| High-income Asia Pacific | 1990 | 2021 | AAPC | 0.744 | 0.647 | 0.841 | <0.001 |
| High-income North America | 1990 | 2021 | AAPC | 1.585 | 1.303 | 1.868 | <0.001 |
| North Africa and Middle East | 1990 | 2021 | AAPC | 0.657 | 0.545 | 0.769 | <0.001 |
| Oceania | 1990 | 2021 | AAPC | 0.114 | 0.038 | 0.19 | 0.003 |
| South Asia | 1990 | 2021 | AAPC | 0.178 | -0.218 | 0.576 | 0.379 |
| Southeast Asia | 1990 | 2021 | AAPC | 0.576 | 0.526 | 0.626 | <0.001 |
| Southern Latin America | 1990 | 2021 | AAPC | 0.516 | 0.128 | 0.905 | 0.009 |
| Southern Sub-Saharan Africa | 1990 | 2021 | AAPC | 0.987 | 0.775 | 1.2 | <0.001 |
| Tropical Latin America | 1990 | 2021 | AAPC | 0.706 | 0.646 | 0.766 | <0.001 |
| Western Europe | 1990 | 2021 | AAPC | 0.795 | 0.665 | 0.925 | <0.001 |
| Western Sub-Saharan Africa | 1990 | 2021 | AAPC | 0.063 | -0.132 | 0.259 | 0.527 |
| Global | 1990 | 2005 | APC | 0.045 | -0.027 | 0.117 | 0.21 |
| Global | 2005 | 2010 | APC | -1.616 | -2.141 | -1.087 | <0.001 |
| Global | 2010 | 2019 | APC | 0.451 | 0.264 | 0.638 | <0.001 |
| Global | 2019 | 2021 | APC | 10.308 | 8.38 | 12.271 | <0.001 |
| High-middle SDI | 1990 | 2010 | APC | -0.693 | -0.756 | -0.631 | <0.001 |
| High-middle SDI | 2010 | 2019 | APC | 0.747 | 0.495 | 1 | <0.001 |
| High-middle SDI | 2019 | 2021 | APC | 8.453 | 5.866 | 11.103 | <0.001 |
| High SDI | 1990 | 2000 | APC | 1.205 | 1.127 | 1.284 | <0.001 |
| High SDI | 2000 | 2019 | APC | -0.162 | -0.194 | -0.129 | <0.001 |
| High SDI | 2019 | 2021 | APC | 13.776 | 12.613 | 14.952 | <0.001 |
| Low-middle SDI | 1990 | 2005 | APC | 0.273 | 0.166 | 0.381 | <0.001 |
| Low-middle SDI | 2005 | 2010 | APC | -3.11 | -3.871 | -2.342 | <0.001 |
| Low-middle SDI | 2010 | 2019 | APC | 0.145 | -0.129 | 0.42 | 0.285 |
| Low-middle SDI | 2019 | 2021 | APC | 11.367 | 8.525 | 14.284 | <0.001 |
| Low SDI | 1990 | 2005 | APC | 0.113 | 0.052 | 0.174 | 0.001 |
| Low SDI | 2005 | 2011 | APC | -1.668 | -1.982 | -1.354 | <0.001 |
| Low SDI | 2011 | 2019 | APC | 0.188 | -0.006 | 0.382 | 0.057 |
| Low SDI | 2019 | 2021 | APC | 7.34 | 5.777 | 8.927 | <0.001 |
| Middle SDI | 1990 | 2012 | APC | -0.446 | -0.526 | -0.365 | <0.001 |
| Middle SDI | 2012 | 2019 | APC | 0.738 | 0.161 | 1.318 | 0.014 |
| Middle SDI | 2019 | 2021 | APC | 9.833 | 6.094 | 13.704 | <0.001 |
| Andean Latin America | 1990 | 2018 | APC | -0.31 | -0.474 | -0.147 | 0.001 |
| Andean Latin America | 2018 | 2021 | APC | 14.93 | 9.087 | 21.085 | <0.001 |
| Australasia | 1990 | 2000 | APC | 0.604 | 0.561 | 0.647 | <0.001 |
| Australasia | 2000 | 2005 | APC | 1.68 | 1.511 | 1.849 | <0.001 |
| Australasia | 2005 | 2019 | APC | -0.825 | -0.854 | -0.796 | <0.001 |
| Australasia | 2019 | 2021 | APC | 5.017 | 4.336 | 5.702 | <0.001 |
| Caribbean | 1990 | 2011 | APC | -0.947 | -0.968 | -0.926 | <0.001 |
| Caribbean | 2011 | 2019 | APC | 0.436 | 0.323 | 0.55 | <0.001 |
| Caribbean | 2019 | 2021 | APC | 12.407 | 11.414 | 13.409 | <0.001 |
| Central Asia | 1990 | 2011 | APC | -0.077 | -0.117 | -0.037 | 0.001 |
| Central Asia | 2011 | 2019 | APC | 0.535 | 0.321 | 0.749 | <0.001 |
| Central Asia | 2019 | 2021 | APC | 10.301 | 8.434 | 12.201 | <0.001 |
| Central Europe | 1990 | 1998 | APC | -0.305 | -0.429 | -0.181 | <0.001 |
| Central Europe | 1998 | 2011 | APC | -1.057 | -1.124 | -0.991 | <0.001 |
| Central Europe | 2011 | 2019 | APC | 0.804 | 0.648 | 0.96 | <0.001 |
| Central Europe | 2019 | 2021 | APC | 14.302 | 12.943 | 15.677 | <0.001 |
| Central Latin America | 1990 | 2019 | APC | 0.862 | 0.807 | 0.917 | <0.001 |
| Central Latin America | 2019 | 2021 | APC | 14.06 | 10.081 | 18.182 | <0.001 |
| Central Sub-Saharan Africa | 1990 | 2018 | APC | -0.111 | -0.178 | -0.044 | 0.002 |
| Central Sub-Saharan Africa | 2018 | 2021 | APC | 4.075 | 1.967 | 6.226 | <0.001 |
| East Asia | 1990 | 1995 | APC | -0.413 | -1.127 | 0.305 | 0.244 |
| East Asia | 1995 | 1999 | APC | -2.779 | -4.281 | -1.254 | 0.001 |
| East Asia | 1999 | 2009 | APC | -1.422 | -1.699 | -1.144 | <0.001 |
| East Asia | 2009 | 2021 | APC | 0.726 | 0.541 | 0.911 | <0.001 |
| Eastern Europe | 1990 | 1995 | APC | 0.971 | 0.893 | 1.048 | <0.001 |
| Eastern Europe | 1995 | 2000 | APC | -1.265 | -1.37 | -1.16 | <0.001 |
| Eastern Europe | 2000 | 2010 | APC | -0.576 | -0.606 | -0.546 | <0.001 |
| Eastern Europe | 2010 | 2015 | APC | 1.2 | 1.093 | 1.308 | <0.001 |
| Eastern Europe | 2015 | 2019 | APC | -0.082 | -0.254 | 0.091 | 0.33 |
| Eastern Europe | 2019 | 2021 | APC | 14.687 | 14.293 | 15.082 | <0.001 |
| Eastern Sub-Saharan Africa | 1990 | 2002 | APC | 0.019 | -0.025 | 0.062 | 0.386 |
| Eastern Sub-Saharan Africa | 2002 | 2013 | APC | -0.746 | -0.802 | -0.689 | <0.001 |
| Eastern Sub-Saharan Africa | 2013 | 2019 | APC | 0.339 | 0.17 | 0.509 | <0.001 |
| Eastern Sub-Saharan Africa | 2019 | 2021 | APC | 8.145 | 7.314 | 8.982 | <0.001 |
| High-income Asia Pacific | 1990 | 1995 | APC | -0.601 | -0.785 | -0.416 | <0.001 |
| High-income Asia Pacific | 1995 | 2000 | APC | 2.741 | 2.465 | 3.017 | <0.001 |
| High-income Asia Pacific | 2000 | 2006 | APC | 0.158 | -0.032 | 0.349 | 0.097 |
| High-income Asia Pacific | 2006 | 2015 | APC | -0.893 | -0.982 | -0.804 | <0.001 |
| High-income Asia Pacific | 2015 | 2019 | APC | 0.044 | -0.377 | 0.467 | 0.827 |
| High-income Asia Pacific | 2019 | 2021 | APC | 10.203 | 9.243 | 11.172 | <0.001 |
| High-income North America | 1990 | 1999 | APC | 2.954 | 2.566 | 3.343 | <0.001 |
| High-income North America | 1999 | 2019 | APC | -0.322 | -0.442 | -0.202 | <0.001 |
| High-income North America | 2019 | 2021 | APC | 15.607 | 11.036 | 20.366 | <0.001 |
| North Africa and Middle East | 1990 | 2002 | APC | -0.245 | -0.334 | -0.155 | <0.001 |
| North Africa and Middle East | 2002 | 2019 | APC | 0.381 | 0.321 | 0.441 | <0.001 |
| North Africa and Middle East | 2019 | 2021 | APC | 8.752 | 6.964 | 10.569 | <0.001 |
| Oceania | 1990 | 2006 | APC | -0.033 | -0.062 | -0.003 | 0.032 |
| Oceania | 2006 | 2010 | APC | -0.553 | -0.949 | -0.156 | 0.009 |
| Oceania | 2010 | 2019 | APC | -0.004 | -0.087 | 0.079 | 0.92 |
| Oceania | 2019 | 2021 | APC | 3.207 | 2.337 | 4.085 | <0.001 |
| South Asia | 1990 | 2005 | APC | 0.295 | 0.099 | 0.491 | 0.005 |
| South Asia | 2005 | 2010 | APC | -4.475 | -5.852 | -3.079 | <0.001 |
| South Asia | 2010 | 2019 | APC | 0.047 | -0.452 | 0.55 | 0.846 |
| South Asia | 2019 | 2021 | APC | 12.504 | 7.34 | 17.917 | <0.001 |
| Southeast Asia | 1990 | 2000 | APC | -0.328 | -0.357 | -0.299 | <0.001 |
| Southeast Asia | 2000 | 2005 | APC | 0.216 | 0.099 | 0.334 | 0.001 |
| Southeast Asia | 2005 | 2010 | APC | -0.493 | -0.61 | -0.376 | <0.001 |
| Southeast Asia | 2010 | 2013 | APC | 0.326 | -0.041 | 0.696 | 0.078 |
| Southeast Asia | 2013 | 2019 | APC | 0.527 | 0.443 | 0.612 | <0.001 |
| Southeast Asia | 2019 | 2021 | APC | 9.601 | 9.186 | 10.017 | <0.001 |
| Southern Latin America | 1990 | 2019 | APC | -0.483 | -0.571 | -0.394 | <0.001 |
| Southern Latin America | 2019 | 2021 | APC | 16.169 | 9.258 | 23.518 | <0.001 |
| Southern Sub-Saharan Africa | 1990 | 2005 | APC | -0.531 | -0.659 | -0.402 | <0.001 |
| Southern Sub-Saharan Africa | 2005 | 2019 | APC | 0.97 | 0.807 | 1.132 | <0.001 |
| Southern Sub-Saharan Africa | 2019 | 2021 | APC | 13.275 | 9.82 | 16.839 | <0.001 |
| Tropical Latin America | 1990 | 1995 | APC | 0.23 | 0.11 | 0.35 | 0.001 |
| Tropical Latin America | 1995 | 2000 | APC | 2.938 | 2.766 | 3.11 | <0.001 |
| Tropical Latin America | 2000 | 2006 | APC | 0.071 | -0.044 | 0.186 | 0.21 |
| Tropical Latin America | 2006 | 2010 | APC | -5.091 | -5.335 | -4.847 | <0.001 |
| Tropical Latin America | 2010 | 2019 | APC | -0.469 | -0.525 | -0.413 | <0.001 |
| Tropical Latin America | 2019 | 2021 | APC | 16.704 | 16.08 | 17.332 | <0.001 |
| Western Europe | 1990 | 2019 | APC | -0.007 | -0.038 | 0.023 | 0.636 |
| Western Europe | 2019 | 2021 | APC | 13.174 | 10.88 | 15.516 | <0.001 |
| Western Sub-Saharan Africa | 1990 | 2004 | APC | 0.206 | 0.021 | 0.391 | 0.03 |
| Western Sub-Saharan Africa | 2004 | 2017 | APC | -0.739 | -0.967 | -0.511 | <0.001 |
| Western Sub-Saharan Africa | 2017 | 2021 | APC | 2.204 | 0.93 | 3.494 | 0.001 |

| **Table S10.** Temporal Joinpoint analysis of anxiety disorders age-standardized prevalence rates, 1990-2021 | | | | | | | |
| --- | --- | --- | --- | --- | --- | --- | --- |
| location | Start.Obs | End.Obs | measure | val | lower | upper | P.Value |
| Global | 1990 | 2021 | AAPC | 0.752 | 0.597 | 0.907 | <0.001 |
| High-middle SDI | 1990 | 2021 | AAPC | 0.564 | 0.531 | 0.596 | <0.001 |
| High SDI | 1990 | 2021 | AAPC | 0.835 | 0.595 | 1.076 | <0.001 |
| Low-middle SDI | 1990 | 2021 | AAPC | 0.852 | 0.802 | 0.901 | <0.001 |
| Low SDI | 1990 | 2021 | AAPC | 0.542 | 0.483 | 0.601 | <0.001 |
| Middle SDI | 1990 | 2021 | AAPC | 0.927 | 0.767 | 1.087 | <0.001 |
| Andean Latin America | 1990 | 2021 | AAPC | 1.439 | 1.349 | 1.53 | <0.001 |
| Australasia | 1990 | 2021 | AAPC | 0.313 | 0.192 | 0.435 | <0.001 |
| Caribbean | 1990 | 2021 | AAPC | 0.788 | 0.722 | 0.853 | <0.001 |
| Central Asia | 1990 | 2021 | AAPC | 0.761 | 0.723 | 0.799 | <0.001 |
| Central Europe | 1990 | 2021 | AAPC | 1.03 | 0.966 | 1.094 | <0.001 |
| Central Latin America | 1990 | 2021 | AAPC | 1.344 | 0.835 | 1.855 | <0.001 |
| Central Sub-Saharan Africa | 1990 | 2021 | AAPC | 0.416 | 0.237 | 0.594 | <0.001 |
| East Asia | 1990 | 2021 | AAPC | -0.022 | -0.304 | 0.261 | 0.88 |
| Eastern Europe | 1990 | 2021 | AAPC | 0.963 | 0.911 | 1.015 | <0.001 |
| Eastern Sub-Saharan Africa | 1990 | 2021 | AAPC | 0.543 | 0.452 | 0.634 | <0.001 |
| High-income Asia Pacific | 1990 | 2021 | AAPC | 0.477 | 0.359 | 0.596 | <0.001 |
| High-income North America | 1990 | 2021 | AAPC | 1.036 | 0.814 | 1.259 | <0.001 |
| North Africa and Middle East | 1990 | 2021 | AAPC | 0.712 | 0.541 | 0.884 | <0.001 |
| Oceania | 1990 | 2021 | AAPC | 0.355 | 0.258 | 0.452 | <0.001 |
| South Asia | 1990 | 2021 | AAPC | 0.816 | 0.736 | 0.896 | <0.001 |
| Southeast Asia | 1990 | 2021 | AAPC | 0.875 | 0.855 | 0.895 | <0.001 |
| Southern Latin America | 1990 | 2021 | AAPC | 0.844 | 0.813 | 0.874 | <0.001 |
| Southern Sub-Saharan Africa | 1990 | 2021 | AAPC | 1.052 | 0.937 | 1.168 | <0.001 |
| Tropical Latin America | 1990 | 2021 | AAPC | 1.87 | 1.642 | 2.098 | <0.001 |
| Western Europe | 1990 | 2021 | AAPC | 0.686 | 0.485 | 0.888 | <0.001 |
| Western Sub-Saharan Africa | 1990 | 2021 | AAPC | 0.292 | -0.024 | 0.608 | 0.07 |
| Global | 1990 | 2002 | APC | 0.329 | 0.199 | 0.459 | <0.001 |
| Global | 2002 | 2019 | APC | -0.19 | -0.273 | -0.108 | <0.001 |
| Global | 2019 | 2021 | APC | 11.916 | 9.393 | 14.498 | <0.001 |
| High-middle SDI | 1990 | 1996 | APC | -0.443 | -0.491 | -0.395 | <0.001 |
| High-middle SDI | 1996 | 2000 | APC | 0.433 | 0.298 | 0.569 | <0.001 |
| High-middle SDI | 2000 | 2005 | APC | -0.674 | -0.755 | -0.593 | <0.001 |
| High-middle SDI | 2005 | 2009 | APC | 0.151 | 0.028 | 0.274 | 0.019 |
| High-middle SDI | 2009 | 2019 | APC | -0.27 | -0.294 | -0.246 | <0.001 |
| High-middle SDI | 2019 | 2021 | APC | 12.662 | 12.326 | 13 | <0.001 |
| High SDI | 1990 | 1996 | APC | 0.349 | 0.03 | 0.669 | 0.034 |
| High SDI | 1996 | 1999 | APC | 3.361 | 1.626 | 5.125 | 0.001 |
| High SDI | 1999 | 2015 | APC | -0.868 | -0.942 | -0.795 | <0.001 |
| High SDI | 2015 | 2019 | APC | 1.707 | 0.695 | 2.729 | 0.002 |
| High SDI | 2019 | 2021 | APC | 11.043 | 8.845 | 13.285 | <0.001 |
| Low-middle SDI | 1990 | 1998 | APC | -0.354 | -0.425 | -0.282 | <0.001 |
| Low-middle SDI | 1998 | 2009 | APC | 0.854 | 0.805 | 0.902 | <0.001 |
| Low-middle SDI | 2009 | 2019 | APC | -0.188 | -0.247 | -0.129 | <0.001 |
| Low-middle SDI | 2019 | 2021 | APC | 11.433 | 10.722 | 12.149 | <0.001 |
| Low SDI | 1990 | 2004 | APC | -0.177 | -0.218 | -0.137 | <0.001 |
| Low SDI | 2004 | 2019 | APC | 0.261 | 0.22 | 0.302 | <0.001 |
| Low SDI | 2019 | 2021 | APC | 7.969 | 7.038 | 8.907 | <0.001 |
| Middle SDI | 1990 | 1995 | APC | -0.064 | -0.514 | 0.389 | 0.772 |
| Middle SDI | 1995 | 2002 | APC | 1.122 | 0.789 | 1.456 | <0.001 |
| Middle SDI | 2002 | 2019 | APC | -0.189 | -0.262 | -0.117 | <0.001 |
| Middle SDI | 2019 | 2021 | APC | 12.935 | 10.754 | 15.159 | <0.001 |
| Andean Latin America | 1990 | 2019 | APC | 0.138 | 0.119 | 0.158 | <0.001 |
| Andean Latin America | 2019 | 2021 | APC | 22.316 | 20.586 | 24.071 | <0.001 |
| Australasia | 1990 | 2000 | APC | -0.46 | -0.578 | -0.342 | <0.001 |
| Australasia | 2000 | 2005 | APC | 1.693 | 1.415 | 1.973 | <0.001 |
| Australasia | 2005 | 2018 | APC | -0.28 | -0.354 | -0.207 | <0.001 |
| Australasia | 2018 | 2021 | APC | 3.236 | 2.064 | 4.422 | <0.001 |
| Caribbean | 1990 | 2019 | APC | 0.088 | 0.073 | 0.103 | <0.001 |
| Caribbean | 2019 | 2021 | APC | 11.499 | 10.359 | 12.651 | <0.001 |
| Central Asia | 1990 | 2019 | APC | -0.027 | -0.036 | -0.018 | <0.001 |
| Central Asia | 2019 | 2021 | APC | 12.914 | 12.237 | 13.594 | <0.001 |
| Central Europe | 1990 | 2019 | APC | 0.037 | 0.022 | 0.052 | <0.001 |
| Central Europe | 2019 | 2021 | APC | 16.576 | 15.409 | 17.756 | <0.001 |
| Central Latin America | 1990 | 1995 | APC | 0.015 | -1.652 | 1.71 | 0.986 |
| Central Latin America | 1995 | 2003 | APC | 2.386 | 1.43 | 3.352 | <0.001 |
| Central Latin America | 2003 | 2018 | APC | -0.558 | -0.874 | -0.241 | 0.001 |
| Central Latin America | 2018 | 2021 | APC | 10.823 | 6.786 | 15.013 | <0.001 |
| Central Sub-Saharan Africa | 1990 | 2018 | APC | -0.022 | -0.079 | 0.035 | 0.44 |
| Central Sub-Saharan Africa | 2018 | 2021 | APC | 4.59 | 2.672 | 6.544 | <0.001 |
| East Asia | 1990 | 2001 | APC | 0.285 | 0.111 | 0.46 | 0.003 |
| East Asia | 2001 | 2004 | APC | -2.581 | -4.901 | -0.205 | 0.035 |
| East Asia | 2004 | 2019 | APC | -0.953 | -1.065 | -0.842 | <0.001 |
| East Asia | 2019 | 2021 | APC | 9.639 | 6.786 | 12.568 | <0.001 |
| Eastern Europe | 1990 | 2001 | APC | -0.374 | -0.419 | -0.329 | <0.001 |
| Eastern Europe | 2001 | 2010 | APC | 0.326 | 0.254 | 0.398 | <0.001 |
| Eastern Europe | 2010 | 2019 | APC | 0.001 | -0.072 | 0.073 | 0.987 |
| Eastern Europe | 2019 | 2021 | APC | 16.711 | 15.935 | 17.491 | <0.001 |
| Eastern Sub-Saharan Africa | 1990 | 2001 | APC | -0.061 | -0.126 | 0.005 | 0.069 |
| Eastern Sub-Saharan Africa | 2001 | 2005 | APC | -0.879 | -1.367 | -0.388 | 0.001 |
| Eastern Sub-Saharan Africa | 2005 | 2019 | APC | 0.24 | 0.189 | 0.292 | <0.001 |
| Eastern Sub-Saharan Africa | 2019 | 2021 | APC | 9.214 | 8.141 | 10.298 | <0.001 |
| High-income Asia Pacific | 1990 | 2000 | APC | 0.386 | 0.297 | 0.476 | <0.001 |
| High-income Asia Pacific | 2000 | 2010 | APC | -0.828 | -0.909 | -0.748 | <0.001 |
| High-income Asia Pacific | 2010 | 2015 | APC | 0.531 | 0.259 | 0.805 | 0.001 |
| High-income Asia Pacific | 2015 | 2019 | APC | -2.173 | -2.678 | -1.665 | <0.001 |
| High-income Asia Pacific | 2019 | 2021 | APC | 13.521 | 11.942 | 15.122 | <0.001 |
| High-income North America | 1990 | 1995 | APC | 0.147 | -0.295 | 0.59 | 0.494 |
| High-income North America | 1995 | 2000 | APC | 5.064 | 4.403 | 5.729 | <0.001 |
| High-income North America | 2000 | 2015 | APC | -1.892 | -1.987 | -1.797 | <0.001 |
| High-income North America | 2015 | 2019 | APC | 3.357 | 2.253 | 4.474 | <0.001 |
| High-income North America | 2019 | 2021 | APC | 11.61 | 9.254 | 14.016 | <0.001 |
| North Africa and Middle East | 1990 | 2019 | APC | 0.148 | 0.11 | 0.187 | <0.001 |
| North Africa and Middle East | 2019 | 2021 | APC | 9.256 | 6.343 | 12.249 | <0.001 |
| Oceania | 1990 | 2019 | APC | 0.013 | -0.007 | 0.033 | 0.196 |
| Oceania | 2019 | 2021 | APC | 5.45 | 3.839 | 7.087 | <0.001 |
| South Asia | 1990 | 2000 | APC | -1.007 | -1.087 | -0.926 | <0.001 |
| South Asia | 2000 | 2011 | APC | 1.083 | 1.003 | 1.162 | <0.001 |
| South Asia | 2011 | 2019 | APC | 0.094 | -0.041 | 0.228 | 0.161 |
| South Asia | 2019 | 2021 | APC | 12.03 | 10.886 | 13.185 | <0.001 |
| Southeast Asia | 1990 | 2001 | APC | 0.049 | 0.038 | 0.06 | <0.001 |
| Southeast Asia | 2001 | 2005 | APC | -0.071 | -0.145 | 0.003 | 0.059 |
| Southeast Asia | 2005 | 2016 | APC | 0.222 | 0.211 | 0.234 | <0.001 |
| Southeast Asia | 2016 | 2019 | APC | 0.411 | 0.257 | 0.566 | <0.001 |
| Southeast Asia | 2019 | 2021 | APC | 12.23 | 12.059 | 12.403 | <0.001 |
| Southern Latin America | 1990 | 2005 | APC | 0.009 | -0.005 | 0.023 | 0.203 |
| Southern Latin America | 2005 | 2010 | APC | -1.046 | -1.145 | -0.947 | <0.001 |
| Southern Latin America | 2010 | 2019 | APC | 0.108 | 0.082 | 0.134 | <0.001 |
| Southern Latin America | 2019 | 2021 | APC | 16.296 | 15.83 | 16.764 | <0.001 |
| Southern Sub-Saharan Africa | 1990 | 2000 | APC | 0.373 | 0.245 | 0.5 | <0.001 |
| Southern Sub-Saharan Africa | 2000 | 2019 | APC | -0.065 | -0.117 | -0.013 | 0.017 |
| Southern Sub-Saharan Africa | 2019 | 2021 | APC | 16.162 | 14.231 | 18.126 | <0.001 |
| Tropical Latin America | 1990 | 1995 | APC | 0.6 | 0.177 | 1.025 | 0.009 |
| Tropical Latin America | 1995 | 2004 | APC | 5.63 | 5.416 | 5.845 | <0.001 |
| Tropical Latin America | 2004 | 2010 | APC | 0.235 | -0.196 | 0.667 | 0.263 |
| Tropical Latin America | 2010 | 2015 | APC | -4.347 | -4.928 | -3.763 | <0.001 |
| Tropical Latin America | 2015 | 2019 | APC | 0.725 | -0.261 | 1.721 | 0.138 |
| Tropical Latin America | 2019 | 2021 | APC | 12.224 | 9.901 | 14.596 | <0.001 |
| Western Europe | 1990 | 2002 | APC | -0.202 | -0.344 | -0.06 | 0.007 |
| Western Europe | 2002 | 2019 | APC | 0.21 | 0.119 | 0.302 | <0.001 |
| Western Europe | 2019 | 2021 | APC | 10.545 | 7.221 | 13.973 | <0.001 |
| Western Sub-Saharan Africa | 1990 | 2005 | APC | -0.32 | -0.481 | -0.159 | <0.001 |
| Western Sub-Saharan Africa | 2005 | 2009 | APC | 1.497 | -0.399 | 3.43 | 0.116 |
| Western Sub-Saharan Africa | 2009 | 2018 | APC | -0.29 | -0.694 | 0.115 | 0.151 |
| Western Sub-Saharan Africa | 2018 | 2021 | APC | 3.564 | 1.648 | 5.518 | 0.001 |

| **Table S11.** Temporal Joinpoint analysis of anxiety disorders age-standardized incidence rates, 1990-2021 | | | | | | | |
| --- | --- | --- | --- | --- | --- | --- | --- |
| location | Start.Obs | End.Obs | measure | val | lower | upper | P.Value |
| Global | 1990 | 2021 | AAPC | 0.719 | 0.553 | 0.884 | <0.001 |
| High-middle SDI | 1990 | 2021 | AAPC | 0.54 | 0.512 | 0.568 | <0.001 |
| High SDI | 1990 | 2021 | AAPC | 0.878 | 0.638 | 1.119 | <0.001 |
| Low-middle SDI | 1990 | 2021 | AAPC | 0.795 | 0.739 | 0.852 | <0.001 |
| Low SDI | 1990 | 2021 | AAPC | 0.538 | 0.466 | 0.61 | <0.001 |
| Middle SDI | 1990 | 2021 | AAPC | 0.914 | 0.746 | 1.082 | <0.001 |
| Andean Latin America | 1990 | 2021 | AAPC | 1.369 | 1.283 | 1.454 | <0.001 |
| Australasia | 1990 | 2021 | AAPC | 0.294 | 0.194 | 0.395 | <0.001 |
| Caribbean | 1990 | 2021 | AAPC | 0.747 | 0.666 | 0.828 | <0.001 |
| Central Asia | 1990 | 2021 | AAPC | 0.735 | 0.705 | 0.765 | <0.001 |
| Central Europe | 1990 | 2021 | AAPC | 1.036 | 0.939 | 1.134 | <0.001 |
| Central Latin America | 1990 | 2021 | AAPC | 1.351 | 0.927 | 1.777 | <0.001 |
| Central Sub-Saharan Africa | 1990 | 2021 | AAPC | 0.424 | 0.245 | 0.604 | <0.001 |
| East Asia | 1990 | 2021 | AAPC | 0.013 | -0.099 | 0.125 | 0.825 |
| Eastern Europe | 1990 | 2021 | AAPC | 0.951 | 0.896 | 1.007 | <0.001 |
| Eastern Sub-Saharan Africa | 1990 | 2021 | AAPC | 0.546 | 0.501 | 0.591 | <0.001 |
| High-income Asia Pacific | 1990 | 2021 | AAPC | 0.415 | 0.193 | 0.637 | <0.001 |
| High-income North America | 1990 | 2021 | AAPC | 1.01 | 0.646 | 1.375 | <0.001 |
| North Africa and Middle East | 1990 | 2021 | AAPC | 0.602 | 0.27 | 0.936 | <0.001 |
| Oceania | 1990 | 2021 | AAPC | 0.316 | 0.201 | 0.431 | <0.001 |
| South Asia | 1990 | 2021 | AAPC | 0.823 | 0.59 | 1.058 | <0.001 |
| Southeast Asia | 1990 | 2021 | AAPC | 0.802 | 0.778 | 0.827 | <0.001 |
| Southern Latin America | 1990 | 2021 | AAPC | 0.764 | 0.644 | 0.884 | <0.001 |
| Southern Sub-Saharan Africa | 1990 | 2021 | AAPC | 0.974 | 0.902 | 1.046 | <0.001 |
| Tropical Latin America | 1990 | 2021 | AAPC | 1.438 | 1.269 | 1.608 | <0.001 |
| Western Europe | 1990 | 2021 | AAPC | 0.773 | 0.557 | 0.99 | <0.001 |
| Western Sub-Saharan Africa | 1990 | 2021 | AAPC | 0.308 | -0.123 | 0.741 | 0.162 |
| Global | 1990 | 2019 | APC | 0.032 | -0.009 | 0.074 | 0.123 |
| Global | 2019 | 2021 | APC | 11.217 | 8.365 | 14.145 | <0.001 |
| High-middle SDI | 1990 | 1995 | APC | -0.564 | -0.62 | -0.508 | <0.001 |
| High-middle SDI | 1995 | 2000 | APC | 0.316 | 0.238 | 0.394 | <0.001 |
| High-middle SDI | 2000 | 2005 | APC | -0.814 | -0.887 | -0.741 | <0.001 |
| High-middle SDI | 2005 | 2009 | APC | 0.204 | 0.089 | 0.319 | 0.002 |
| High-middle SDI | 2009 | 2019 | APC | -0.17 | -0.192 | -0.149 | <0.001 |
| High-middle SDI | 2019 | 2021 | APC | 12.141 | 11.86 | 12.423 | <0.001 |
| High SDI | 1990 | 2001 | APC | 0.905 | 0.675 | 1.136 | <0.001 |
| High SDI | 2001 | 2019 | APC | -0.369 | -0.487 | -0.25 | <0.001 |
| High SDI | 2019 | 2021 | APC | 12.657 | 8.755 | 16.7 | <0.001 |
| Low-middle SDI | 1990 | 1997 | APC | -0.576 | -0.675 | -0.478 | <0.001 |
| Low-middle SDI | 1997 | 2010 | APC | 0.591 | 0.55 | 0.633 | <0.001 |
| Low-middle SDI | 2010 | 2019 | APC | -0.11 | -0.189 | -0.03 | 0.009 |
| Low-middle SDI | 2019 | 2021 | APC | 11.588 | 10.777 | 12.404 | <0.001 |
| Low SDI | 1990 | 2004 | APC | -0.177 | -0.228 | -0.126 | <0.001 |
| Low SDI | 2004 | 2019 | APC | 0.231 | 0.18 | 0.282 | <0.001 |
| Low SDI | 2019 | 2021 | APC | 8.149 | 7.019 | 9.29 | <0.001 |
| Middle SDI | 1990 | 2002 | APC | 0.345 | 0.194 | 0.497 | <0.001 |
| Middle SDI | 2002 | 2019 | APC | -0.009 | -0.103 | 0.084 | 0.837 |
| Middle SDI | 2019 | 2021 | APC | 12.879 | 10.16 | 15.666 | <0.001 |
| Andean Latin America | 1990 | 2019 | APC | 0.061 | 0.043 | 0.08 | <0.001 |
| Andean Latin America | 2019 | 2021 | APC | 22.363 | 20.727 | 24.021 | <0.001 |
| Australasia | 1990 | 1999 | APC | 0.009 | -0.084 | 0.103 | 0.838 |
| Australasia | 1999 | 2005 | APC | 2.042 | 1.836 | 2.248 | <0.001 |
| Australasia | 2005 | 2010 | APC | -1.285 | -1.562 | -1.007 | <0.001 |
| Australasia | 2010 | 2019 | APC | -0.493 | -0.602 | -0.385 | <0.001 |
| Australasia | 2019 | 2021 | APC | 3.987 | 2.731 | 5.259 | <0.001 |
| Caribbean | 1990 | 2019 | APC | 0.038 | 0.019 | 0.057 | <0.001 |
| Caribbean | 2019 | 2021 | APC | 11.604 | 10.191 | 13.036 | <0.001 |
| Central Asia | 1990 | 2019 | APC | -0.039 | -0.046 | -0.032 | <0.001 |
| Central Asia | 2019 | 2021 | APC | 12.66 | 12.133 | 13.189 | <0.001 |
| Central Europe | 1990 | 2019 | APC | 0.023 | -0.001 | 0.047 | 0.064 |
| Central Europe | 2019 | 2021 | APC | 16.94 | 15.171 | 18.736 | <0.001 |
| Central Latin America | 1990 | 2005 | APC | 1.113 | 0.792 | 1.435 | <0.001 |
| Central Latin America | 2005 | 2018 | APC | -0.259 | -0.698 | 0.183 | 0.238 |
| Central Latin America | 2018 | 2021 | APC | 9.919 | 5.799 | 14.198 | <0.001 |
| Central Sub-Saharan Africa | 1990 | 2018 | APC | -0.019 | -0.079 | 0.041 | 0.519 |
| Central Sub-Saharan Africa | 2018 | 2021 | APC | 4.658 | 2.739 | 6.614 | <0.001 |
| East Asia | 1990 | 1995 | APC | -0.533 | -0.757 | -0.309 | <0.001 |
| East Asia | 1995 | 2000 | APC | 0.776 | 0.465 | 1.088 | <0.001 |
| East Asia | 2000 | 2004 | APC | -2.241 | -2.682 | -1.797 | <0.001 |
| East Asia | 2004 | 2015 | APC | -0.738 | -0.806 | -0.67 | <0.001 |
| East Asia | 2015 | 2019 | APC | -0.048 | -0.491 | 0.397 | 0.821 |
| East Asia | 2019 | 2021 | APC | 8.659 | 7.631 | 9.696 | <0.001 |
| Eastern Europe | 1990 | 2000 | APC | -0.389 | -0.424 | -0.354 | <0.001 |
| Eastern Europe | 2000 | 2010 | APC | 0.237 | 0.194 | 0.279 | <0.001 |
| Eastern Europe | 2010 | 2016 | APC | -0.194 | -0.296 | -0.093 | 0.001 |
| Eastern Europe | 2016 | 2019 | APC | 0.391 | -0.059 | 0.843 | 0.085 |
| Eastern Europe | 2019 | 2021 | APC | 16.699 | 16.169 | 17.231 | <0.001 |
| Eastern Sub-Saharan Africa | 1990 | 2000 | APC | 0.045 | 0.002 | 0.088 | 0.039 |
| Eastern Sub-Saharan Africa | 2000 | 2005 | APC | -0.76 | -0.934 | -0.586 | <0.001 |
| Eastern Sub-Saharan Africa | 2005 | 2019 | APC | 0.183 | 0.154 | 0.212 | <0.001 |
| Eastern Sub-Saharan Africa | 2019 | 2021 | APC | 9.248 | 8.678 | 9.821 | <0.001 |
| High-income Asia Pacific | 1990 | 2002 | APC | 0.268 | 0.156 | 0.38 | <0.001 |
| High-income Asia Pacific | 2002 | 2016 | APC | -0.688 | -0.777 | -0.599 | <0.001 |
| High-income Asia Pacific | 2016 | 2019 | APC | -2.21 | -4.031 | -0.355 | 0.022 |
| High-income Asia Pacific | 2019 | 2021 | APC | 13.875 | 11.514 | 16.287 | <0.001 |
| High-income North America | 1990 | 1996 | APC | 0.334 | -0.088 | 0.758 | 0.113 |
| High-income North America | 1996 | 1999 | APC | 4.679 | 2.143 | 7.279 | 0.001 |
| High-income North America | 1999 | 2011 | APC | -0.407 | -0.576 | -0.238 | <0.001 |
| High-income North America | 2011 | 2015 | APC | -2.496 | -3.757 | -1.218 | 0.001 |
| High-income North America | 2015 | 2019 | APC | 1.746 | 0.43 | 3.08 | 0.013 |
| High-income North America | 2019 | 2021 | APC | 12.467 | 9.479 | 15.536 | <0.001 |
| North Africa and Middle East | 1990 | 2005 | APC | -0.142 | -0.338 | 0.055 | 0.151 |
| North Africa and Middle East | 2005 | 2019 | APC | 0.531 | 0.276 | 0.787 | <0.001 |
| North Africa and Middle East | 2019 | 2021 | APC | 6.89 | 1.799 | 12.236 | 0.01 |
| Oceania | 1990 | 1994 | APC | -0.461 | -0.902 | -0.018 | 0.042 |
| Oceania | 1994 | 2019 | APC | 0.048 | 0.019 | 0.077 | 0.002 |
| Oceania | 2019 | 2021 | APC | 5.356 | 3.678 | 7.062 | <0.001 |
| South Asia | 1990 | 1996 | APC | -1.112 | -1.682 | -0.538 | 0.001 |
| South Asia | 1996 | 2019 | APC | 0.444 | 0.365 | 0.523 | <0.001 |
| South Asia | 2019 | 2021 | APC | 11.59 | 8.027 | 15.271 | <0.001 |
| Southeast Asia | 1990 | 1994 | APC | -0.343 | -0.411 | -0.276 | <0.001 |
| Southeast Asia | 1994 | 2006 | APC | -0.011 | -0.025 | 0.003 | 0.116 |
| Southeast Asia | 2006 | 2010 | APC | 0.342 | 0.242 | 0.442 | <0.001 |
| Southeast Asia | 2010 | 2015 | APC | -0.137 | -0.2 | -0.075 | <0.001 |
| Southeast Asia | 2015 | 2019 | APC | 0.627 | 0.524 | 0.73 | <0.001 |
| Southeast Asia | 2019 | 2021 | APC | 12.242 | 12.02 | 12.463 | <0.001 |
| Southern Latin America | 1990 | 2009 | APC | 0.295 | 0.249 | 0.341 | <0.001 |
| Southern Latin America | 2009 | 2019 | APC | -0.969 | -1.095 | -0.843 | <0.001 |
| Southern Latin America | 2019 | 2021 | APC | 14.881 | 12.854 | 16.945 | <0.001 |
| Southern Sub-Saharan Africa | 1990 | 2019 | APC | 0.017 | -0.001 | 0.035 | 0.065 |
| Southern Sub-Saharan Africa | 2019 | 2021 | APC | 15.926 | 14.635 | 17.232 | <0.001 |
| Tropical Latin America | 1990 | 1995 | APC | -0.099 | -0.44 | 0.244 | 0.548 |
| Tropical Latin America | 1995 | 2003 | APC | 3.565 | 3.356 | 3.774 | <0.001 |
| Tropical Latin America | 2003 | 2010 | APC | 1.071 | 0.823 | 1.32 | <0.001 |
| Tropical Latin America | 2010 | 2015 | APC | -2.955 | -3.413 | -2.496 | <0.001 |
| Tropical Latin America | 2015 | 2019 | APC | 0.225 | -0.517 | 0.972 | 0.529 |
| Tropical Latin America | 2019 | 2021 | APC | 12.4 | 10.736 | 14.089 | <0.001 |
| Western Europe | 1990 | 2012 | APC | 0.291 | 0.222 | 0.36 | <0.001 |
| Western Europe | 2012 | 2019 | APC | -0.67 | -1.157 | -0.182 | 0.009 |
| Western Europe | 2019 | 2021 | APC | 11.738 | 8.472 | 15.101 | <0.001 |
| Western Sub-Saharan Africa | 1990 | 1995 | APC | 0.101 | -0.82 | 1.03 | 0.82 |
| Western Sub-Saharan Africa | 1995 | 2000 | APC | -1.601 | -2.894 | -0.291 | 0.02 |
| Western Sub-Saharan Africa | 2000 | 2005 | APC | 0.023 | -1.286 | 1.348 | 0.971 |
| Western Sub-Saharan Africa | 2005 | 2010 | APC | 1.681 | 0.346 | 3.033 | 0.017 |
| Western Sub-Saharan Africa | 2010 | 2018 | APC | -0.213 | -0.765 | 0.341 | 0.424 |
| Western Sub-Saharan Africa | 2018 | 2021 | APC | 3.514 | 1.382 | 5.69 | 0.003 |

| **Table S12.** Temporal Joinpoint analysis of anxiety disorders age-standardized DALYs rates, 1990-2021 | | | | | | | |
| --- | --- | --- | --- | --- | --- | --- | --- |
| location | Start.Obs | End.Obs | measure | val | lower | upper | P.Value |
| Global | 1990 | 2021 | AAPC | 0.734 | 0.584 | 0.884 | <0.001 |
| High-middle SDI | 1990 | 2021 | AAPC | 0.545 | 0.511 | 0.578 | <0.001 |
| High SDI | 1990 | 2021 | AAPC | 0.81 | 0.593 | 1.028 | <0.001 |
| Low-middle SDI | 1990 | 2021 | AAPC | 0.852 | 0.804 | 0.9 | <0.001 |
| Low SDI | 1990 | 2021 | AAPC | 0.566 | 0.498 | 0.633 | <0.001 |
| Middle SDI | 1990 | 2021 | AAPC | 0.905 | 0.747 | 1.063 | <0.001 |
| Andean Latin America | 1990 | 2021 | AAPC | 1.426 | 1.344 | 1.508 | <0.001 |
| Australasia | 1990 | 2021 | AAPC | 0.3 | 0.156 | 0.445 | <0.001 |
| Caribbean | 1990 | 2021 | AAPC | 0.769 | 0.713 | 0.825 | <0.001 |
| Central Asia | 1990 | 2021 | AAPC | 0.76 | 0.718 | 0.802 | <0.001 |
| Central Europe | 1990 | 2021 | AAPC | 1.025 | 0.968 | 1.082 | <0.001 |
| Central Latin America | 1990 | 2021 | AAPC | 1.325 | 0.828 | 1.825 | <0.001 |
| Central Sub-Saharan Africa | 1990 | 2021 | AAPC | 0.451 | 0.267 | 0.635 | <0.001 |
| East Asia | 1990 | 2021 | AAPC | -0.022 | -0.31 | 0.267 | 0.88 |
| Eastern Europe | 1990 | 2021 | AAPC | 0.948 | 0.896 | 1 | <0.001 |
| Eastern Sub-Saharan Africa | 1990 | 2021 | AAPC | 0.567 | 0.473 | 0.661 | <0.001 |
| High-income Asia Pacific | 1990 | 2021 | AAPC | 0.472 | 0.364 | 0.58 | <0.001 |
| High-income North America | 1990 | 2021 | AAPC | 0.999 | 0.789 | 1.209 | <0.001 |
| North Africa and Middle East | 1990 | 2021 | AAPC | 0.692 | 0.543 | 0.841 | <0.001 |
| Oceania | 1990 | 2021 | AAPC | 0.36 | 0.277 | 0.444 | <0.001 |
| South Asia | 1990 | 2021 | AAPC | 0.826 | 0.747 | 0.905 | <0.001 |
| Southeast Asia | 1990 | 2021 | AAPC | 0.874 | 0.851 | 0.897 | <0.001 |
| Southern Latin America | 1990 | 2021 | AAPC | 0.747 | 0.537 | 0.957 | <0.001 |
| Southern Sub-Saharan Africa | 1990 | 2021 | AAPC | 0.995 | 0.882 | 1.108 | <0.001 |
| Tropical Latin America | 1990 | 2021 | AAPC | 1.855 | 1.644 | 2.066 | <0.001 |
| Western Europe | 1990 | 2021 | AAPC | 0.738 | 0.516 | 0.96 | <0.001 |
| Western Sub-Saharan Africa | 1990 | 2021 | AAPC | 0.314 | -0.001 | 0.629 | 0.051 |
| Global | 1990 | 2002 | APC | 0.319 | 0.19 | 0.45 | <0.001 |
| Global | 2002 | 2019 | APC | -0.189 | -0.272 | -0.106 | <0.001 |
| Global | 2019 | 2021 | APC | 11.653 | 9.232 | 14.128 | <0.001 |
| High-middle SDI | 1990 | 1996 | APC | -0.45 | -0.497 | -0.404 | <0.001 |
| High-middle SDI | 1996 | 2000 | APC | 0.435 | 0.298 | 0.573 | <0.001 |
| High-middle SDI | 2000 | 2005 | APC | -0.664 | -0.749 | -0.579 | <0.001 |
| High-middle SDI | 2005 | 2009 | APC | 0.153 | 0.019 | 0.287 | 0.028 |
| High-middle SDI | 2009 | 2019 | APC | -0.29 | -0.315 | -0.265 | <0.001 |
| High-middle SDI | 2019 | 2021 | APC | 12.43 | 12.108 | 12.754 | <0.001 |
| High SDI | 1990 | 1996 | APC | 0.333 | 0.046 | 0.621 | 0.025 |
| High SDI | 1996 | 1999 | APC | 3.293 | 1.634 | 4.98 | 0.001 |
| High SDI | 1999 | 2015 | APC | -0.859 | -0.927 | -0.79 | <0.001 |
| High SDI | 2015 | 2019 | APC | 1.604 | 0.734 | 2.48 | 0.001 |
| High SDI | 2019 | 2021 | APC | 10.922 | 9.025 | 12.852 | <0.001 |
| Low-middle SDI | 1990 | 1998 | APC | -0.343 | -0.411 | -0.275 | <0.001 |
| Low-middle SDI | 1998 | 2009 | APC | 0.871 | 0.824 | 0.919 | <0.001 |
| Low-middle SDI | 2009 | 2019 | APC | -0.181 | -0.238 | -0.124 | <0.001 |
| Low-middle SDI | 2019 | 2021 | APC | 11.248 | 10.554 | 11.945 | <0.001 |
| Low SDI | 1990 | 2003 | APC | -0.163 | -0.213 | -0.112 | <0.001 |
| Low SDI | 2003 | 2019 | APC | 0.276 | 0.236 | 0.317 | <0.001 |
| Low SDI | 2019 | 2021 | APC | 7.889 | 6.822 | 8.967 | <0.001 |
| Middle SDI | 1990 | 1995 | APC | -0.064 | -0.503 | 0.376 | 0.764 |
| Middle SDI | 1995 | 2002 | APC | 1.097 | 0.767 | 1.428 | <0.001 |
| Middle SDI | 2002 | 2019 | APC | -0.196 | -0.27 | -0.122 | <0.001 |
| Middle SDI | 2019 | 2021 | APC | 12.719 | 10.57 | 14.91 | <0.001 |
| Andean Latin America | 1990 | 2019 | APC | 0.153 | 0.134 | 0.172 | <0.001 |
| Andean Latin America | 2019 | 2021 | APC | 21.807 | 20.26 | 23.374 | <0.001 |
| Australasia | 1990 | 2000 | APC | -0.477 | -0.626 | -0.327 | <0.001 |
| Australasia | 2000 | 2005 | APC | 1.698 | 1.145 | 2.255 | <0.001 |
| Australasia | 2005 | 2018 | APC | -0.263 | -0.374 | -0.151 | <0.001 |
| Australasia | 2018 | 2021 | APC | 3.07 | 1.953 | 4.199 | <0.001 |
| Caribbean | 1990 | 2019 | APC | 0.085 | 0.071 | 0.098 | <0.001 |
| Caribbean | 2019 | 2021 | APC | 11.235 | 10.262 | 12.217 | <0.001 |
| Central Asia | 1990 | 2019 | APC | -0.021 | -0.031 | -0.011 | <0.001 |
| Central Asia | 2019 | 2021 | APC | 12.792 | 12.051 | 13.538 | <0.001 |
| Central Europe | 1990 | 2019 | APC | 0.05 | 0.035 | 0.064 | <0.001 |
| Central Europe | 2019 | 2021 | APC | 16.285 | 15.245 | 17.334 | <0.001 |
| Central Latin America | 1990 | 1995 | APC | 0.03 | -1.557 | 1.643 | 0.969 |
| Central Latin America | 1995 | 2003 | APC | 2.371 | 1.424 | 3.326 | <0.001 |
| Central Latin America | 2003 | 2018 | APC | -0.565 | -0.882 | -0.248 | 0.001 |
| Central Latin America | 2018 | 2021 | APC | 10.668 | 6.705 | 14.779 | <0.001 |
| Central Sub-Saharan Africa | 1990 | 2018 | APC | 0.023 | -0.036 | 0.082 | 0.431 |
| Central Sub-Saharan Africa | 2018 | 2021 | APC | 4.532 | 2.56 | 6.542 | <0.001 |
| East Asia | 1990 | 2001 | APC | 0.276 | 0.103 | 0.449 | 0.003 |
| East Asia | 2001 | 2004 | APC | -2.457 | -4.901 | 0.049 | 0.054 |
| East Asia | 2004 | 2019 | APC | -0.969 | -1.088 | -0.851 | <0.001 |
| East Asia | 2019 | 2021 | APC | 9.613 | 6.882 | 12.415 | <0.001 |
| Eastern Europe | 1990 | 2001 | APC | -0.392 | -0.435 | -0.349 | <0.001 |
| Eastern Europe | 2001 | 2010 | APC | 0.351 | 0.28 | 0.421 | <0.001 |
| Eastern Europe | 2010 | 2019 | APC | -0.01 | -0.08 | 0.06 | 0.766 |
| Eastern Europe | 2019 | 2021 | APC | 16.483 | 15.694 | 17.277 | <0.001 |
| Eastern Sub-Saharan Africa | 1990 | 2001 | APC | -0.043 | -0.111 | 0.026 | 0.207 |
| Eastern Sub-Saharan Africa | 2001 | 2005 | APC | -0.781 | -1.281 | -0.278 | 0.004 |
| Eastern Sub-Saharan Africa | 2005 | 2019 | APC | 0.274 | 0.221 | 0.327 | <0.001 |
| Eastern Sub-Saharan Africa | 2019 | 2021 | APC | 9.034 | 7.91 | 10.169 | <0.001 |
| High-income Asia Pacific | 1990 | 2000 | APC | 0.389 | 0.308 | 0.47 | <0.001 |
| High-income Asia Pacific | 2000 | 2010 | APC | -0.827 | -0.916 | -0.738 | <0.001 |
| High-income Asia Pacific | 2010 | 2015 | APC | 0.534 | 0.221 | 0.848 | 0.002 |
| High-income Asia Pacific | 2015 | 2019 | APC | -2.241 | -2.738 | -1.741 | <0.001 |
| High-income Asia Pacific | 2019 | 2021 | APC | 13.547 | 12.326 | 14.781 | <0.001 |
| High-income North America | 1990 | 1995 | APC | 0.113 | -0.321 | 0.548 | 0.593 |
| High-income North America | 1995 | 2000 | APC | 5.016 | 4.376 | 5.66 | <0.001 |
| High-income North America | 2000 | 2015 | APC | -1.887 | -1.976 | -1.797 | <0.001 |
| High-income North America | 2015 | 2019 | APC | 3.198 | 2.19 | 4.216 | <0.001 |
| High-income North America | 2019 | 2021 | APC | 11.489 | 9.268 | 13.755 | <0.001 |
| North Africa and Middle East | 1990 | 2019 | APC | 0.139 | 0.103 | 0.175 | <0.001 |
| North Africa and Middle East | 2019 | 2021 | APC | 9.064 | 6.533 | 11.655 | <0.001 |
| Oceania | 1990 | 2019 | APC | 0.025 | 0.007 | 0.043 | 0.009 |
| Oceania | 2019 | 2021 | APC | 5.349 | 3.961 | 6.756 | <0.001 |
| South Asia | 1990 | 2000 | APC | -0.989 | -1.069 | -0.909 | <0.001 |
| South Asia | 2000 | 2011 | APC | 1.118 | 1.037 | 1.199 | <0.001 |
| South Asia | 2011 | 2019 | APC | 0.096 | -0.039 | 0.232 | 0.155 |
| South Asia | 2019 | 2021 | APC | 11.873 | 10.744 | 13.013 | <0.001 |
| Southeast Asia | 1990 | 2001 | APC | 0.06 | 0.048 | 0.072 | <0.001 |
| Southeast Asia | 2001 | 2005 | APC | -0.075 | -0.161 | 0.011 | 0.084 |
| Southeast Asia | 2005 | 2016 | APC | 0.231 | 0.217 | 0.244 | <0.001 |
| Southeast Asia | 2016 | 2019 | APC | 0.407 | 0.231 | 0.584 | <0.001 |
| Southeast Asia | 2019 | 2021 | APC | 12.109 | 11.909 | 12.309 | <0.001 |
| Southern Latin America | 1990 | 2019 | APC | -0.21 | -0.258 | -0.163 | <0.001 |
| Southern Latin America | 2019 | 2021 | APC | 15.696 | 11.932 | 19.588 | <0.001 |
| Southern Sub-Saharan Africa | 1990 | 2000 | APC | 0.266 | 0.138 | 0.394 | <0.001 |
| Southern Sub-Saharan Africa | 2000 | 2019 | APC | -0.079 | -0.131 | -0.027 | 0.005 |
| Southern Sub-Saharan Africa | 2019 | 2021 | APC | 15.914 | 14.024 | 17.835 | <0.001 |
| Tropical Latin America | 1990 | 1995 | APC | 0.608 | 0.206 | 1.011 | 0.006 |
| Tropical Latin America | 1995 | 2004 | APC | 5.562 | 5.355 | 5.77 | <0.001 |
| Tropical Latin America | 2004 | 2010 | APC | 0.307 | -0.102 | 0.718 | 0.131 |
| Tropical Latin America | 2010 | 2015 | APC | -4.315 | -4.865 | -3.762 | <0.001 |
| Tropical Latin America | 2015 | 2019 | APC | 0.741 | -0.179 | 1.67 | 0.107 |
| Tropical Latin America | 2019 | 2021 | APC | 11.904 | 9.843 | 14.005 | <0.001 |
| Western Europe | 1990 | 2019 | APC | 0.051 | -0.003 | 0.105 | 0.062 |
| Western Europe | 2019 | 2021 | APC | 11.245 | 7.433 | 15.193 | <0.001 |
| Western Sub-Saharan Africa | 1990 | 2005 | APC | -0.297 | -0.455 | -0.139 | 0.001 |
| Western Sub-Saharan Africa | 2005 | 2009 | APC | 1.549 | -0.338 | 3.471 | 0.103 |
| Western Sub-Saharan Africa | 2009 | 2018 | APC | -0.257 | -0.662 | 0.15 | 0.203 |
| Western Sub-Saharan Africa | 2018 | 2021 | APC | 3.509 | 1.592 | 5.462 | 0.001 |

| **Table S13.** The health-related variables in WCBA across the top 20 and bottom 20 countries and territories in 2021 (sort by 2021 ASIR of depressive disorders) | | | | | |
| --- | --- | --- | --- | --- | --- |
|  | Healthcare resource | |  |  |  |
|  | The Proportion of Male Nursing Personnel(%) | Nursing and Midwifery Personnel Rate (per 10,000) | The Environmental, Social and Governance Index | The Healthcare Index | The Quality of Life Index |
| Top 20 countries and territories | |  |  |  |  |
| Ireland | NA | NA | 150.9 | 52.8 | 20.91 |
| Central African Republic | NA | NA | NA | NA | 71.35 |
| Gambia | NA | NA | NA | NA | 60 |
| Morocco | NA | NA | 104.4 | 45.8 | 49.05 |
| Equatorial Guinea | NA | NA | NA | NA | 61.2 |
| Congo | NA | NA | NA | NA | 69.87 |
| Sweden | NA | NA | 171.4 | 68.8 | 15.7 |
| Angola | NA | NA | NA | NA | 55.68 |
| Iran (Islamic Republic of) | NA | NA | 64.7 | 52.2 | 50.84 |
| Spain | NA | NA | 164.5 | 78.8 | 23.39 |
| Suriname | NA | NA | NA | NA | 42.21 |
| Palestine | NA | NA | NA | NA | NA |
| Tunisia | NA | NA | NA | NA | 38.34 |
| United States of America | NA | 131.5 | 167 | 69 | 34.29 |
| Portugal | NA | NA | 161.9 | 71.9 | 18.68 |
| Lebanon | NA | NA | 94.5 | 63.3 | 50.23 |
| Guyana | NA | NA | NA | NA | 41.88 |
| Lesotho | NA | NA | NA | NA | 55.58 |
| Greece | NA | NA | 129.9 | 57 | 30.45 |
| Greenland | NA | NA | NA | NA | NA |
| Bottom 20 countries and territories | |  |  |  |  |
| Myanmar | NA | NA | NA | NA | 59.27 |
| China | NA | NA | 103.1 | 66.4 | 45.45 |
| Taiwan (Province of China) | NA | NA | 138.8 | 86.4 | 44.41 |
| Lao People's Democratic Republic | NA | NA | NA | NA | 56.93 |
| Democratic People's Republic of Korea | NA | NA | NA | NA | NA |
| Brunei Darussalam | NA | NA | NA | NA | 39.79 |
| American Samoa | NA | NA | NA | NA | NA |
| Colombia | NA | NA | 101.3 | 66.7 | 43.94 |
| Poland | NA | NA | 132.6 | 58.3 | 25.88 |
| Tonga | NA | NA | NA | NA | 44.33 |
| Viet Nam | NA | NA | 88.4 | 58.3 | 44.81 |
| Samoa | NA | NA | NA | NA | 37.2 |
| Indonesia | 25.3 | 40.96 | 90.1 | 60.5 | 47.99 |
| Kiribati | NA | NA | NA | NA | NA |
| Micronesia (Federated States of) | NA | NA | NA | NA | NA |
| Northern Mariana Islands | NA | NA | NA | NA | NA |
| Seychelles | NA | NA | NA | NA | 35.5 |
| Marshall Islands | NA | NA | NA | NA | NA |
| Singapore | NA | NA | 143.8 | 70.9 | 34.85 |
| Timor-Leste | NA | NA | NA | NA | 49.49 |

| **Table S14.** The health-related variables in WCBA across the top 20 and bottom 20 countries and territories in 2021. (sort by 2021 ASIR of anxiety disorders) | | | | | |
| --- | --- | --- | --- | --- | --- |
|  | Healthcare resource | |  |  |  |
|  | The Proportion of Male Nursing Personnel(%) | Nursing and Midwifery Personnel Rate (per 10,000) | The Environmental, Social and Governance Index | The Healthcare Index | The Quality of Life Index |
| Top 20 countries and territories | |  |  |  |  |
| Tunisia | NA | NA | 38.34 | NA | NA |
| Suriname | NA | NA | 42.21 | NA | NA |
| Guyana | NA | NA | 41.88 | NA | NA |
| Argentina | NA | NA | 29.41 | 68.6 | 110.5 |
| Italy | NA | NA | 22.16 | 66.8 | 138.6 |
| Norway | NA | NA | 16.21 | 75.5 | 173.6 |
| France | NA | NA | 22.05 | 81 | 150.7 |
| Uruguay | 16.7 | 72.06 | 25.76 | 67.7 | 124.6 |
| Portugal | NA | NA | 18.68 | 71.9 | 161.9 |
| Chile | NA | 46.01 | 26.98 | 63.7 | 99.9 |
| Iran (Islamic Republic of) | NA | NA | 50.84 | 52.2 | 64.7 |
| Lebanon | NA | NA | 50.23 | 63.3 | 94.5 |
| Ecuador | NA | NA | 37.74 | 68.8 | 118.8 |
| Greenland | NA | NA | NA | NA | NA |
| New Zealand | NA | 114.35 | 18.58 | 73.6 | 175.8 |
| Peru | 11.4 | 26.13 | 41.82 | 56.4 | 83.3 |
| United States of America | NA | 131.5 | 34.29 | 69 | 167 |
| Bolivia (Plurinational State of) | NA | NA | 44.73 | NA | NA |
| Paraguay | 17.7 | 90.3 | 38.69 | NA | NA |
| Brazil | NA | NA | 38.51 | 57.3 | 104.7 |
| Bottom 20 countries and territories |  |  |  |  |  |
| Mongolia | NA | NA | 37.94 | NA | NA |
| Uzbekistan | NA | NA | 40.23 | NA | NA |
| Kazakhstan | NA | NA | 35.49 | 60.1 | 96.4 |
| Niger | NA | NA | 60.29 | NA | NA |
| Mali | NA | NA | 62 | NA | NA |
| Mauritania | NA | 16.05 | 59.26 | NA | NA |
| Kyrgyzstan | NA | NA | 39.31 | NA | NA |
| Burkina Faso | NA | 9.16 | 55.29 | NA | NA |
| Nigeria | NA | 15.64 | 65.65 | 48.9 | 52 |
| Sierra Leone | NA | NA | 64.46 | NA | NA |
| Chad | 64.9 | 1.98 | 69.99 | NA | NA |
| Sao Tome and Principe | NA | NA | 44.98 | NA | NA |
| Cote d'Ivoire | NA | 7.26 | 62.16 | NA | NA |
| Turkmenistan | NA | NA | 41.74 | NA | NA |
| Ghana | 28.3 | 34.2 | 50.96 | NA | NA |
| Benin | NA | 5.82 | 49.75 | NA | NA |
| Cameroon | 40.8 | 1.93 | 61.49 | NA | NA |
| Liberia | NA | 4.92 | 62.75 | NA | NA |
| Togo | NA | 3.97 | 57.95 | NA | NA |
| Guinea-Bissau | 34.4 | 10.53 | 66.43 | NA | NA |

| **Table S15.** Correlation between age-standardized incidence of depressive and anxiety disorders and health-related variables among WCBA in 2021. | | | | | | | |
| --- | --- | --- | --- | --- | --- | --- | --- |
|  | Depression | | |  | Anxiety disorders | | |
|  | r_s_ | p | N |  | r_s_ | p | N |
| The Proportion of Male Nursing Personnel(%) | -0.294 | 0.222 | 19 |  | -0.741 | <0.001 | 19 |
| Nursing and Midwifery Personnel Rate (per 10,000) | 0.385 | 0.019 | 37 |  | 0.723 | <0.001 | 37 |
| The Environmental, Social and Governance Index | -0.120 | 0.140 | 153 |  | 0.418 | <0.001 | 153 |
| The Healthcare Index | 0.183 | 0.122 | 73 |  | 0.256 | 0.029 | 73 |
| The Quality of Life Index | 0.330 | 0.004 | 73 |  | 0.198 | 0.094 | 73 |
