# Supplementary figures and images for: Healthcare Services and Burden of the Top Two Mental Disorders Among Women of Childbearing Age Across 204 Countries and Territories, 1990–2021
Source: Depress Anxiety. 2026 Apr 1;2026:8872094. doi: 10.1155/da/8872094 (PMC13042365; doi:10.1155/da/8872094)

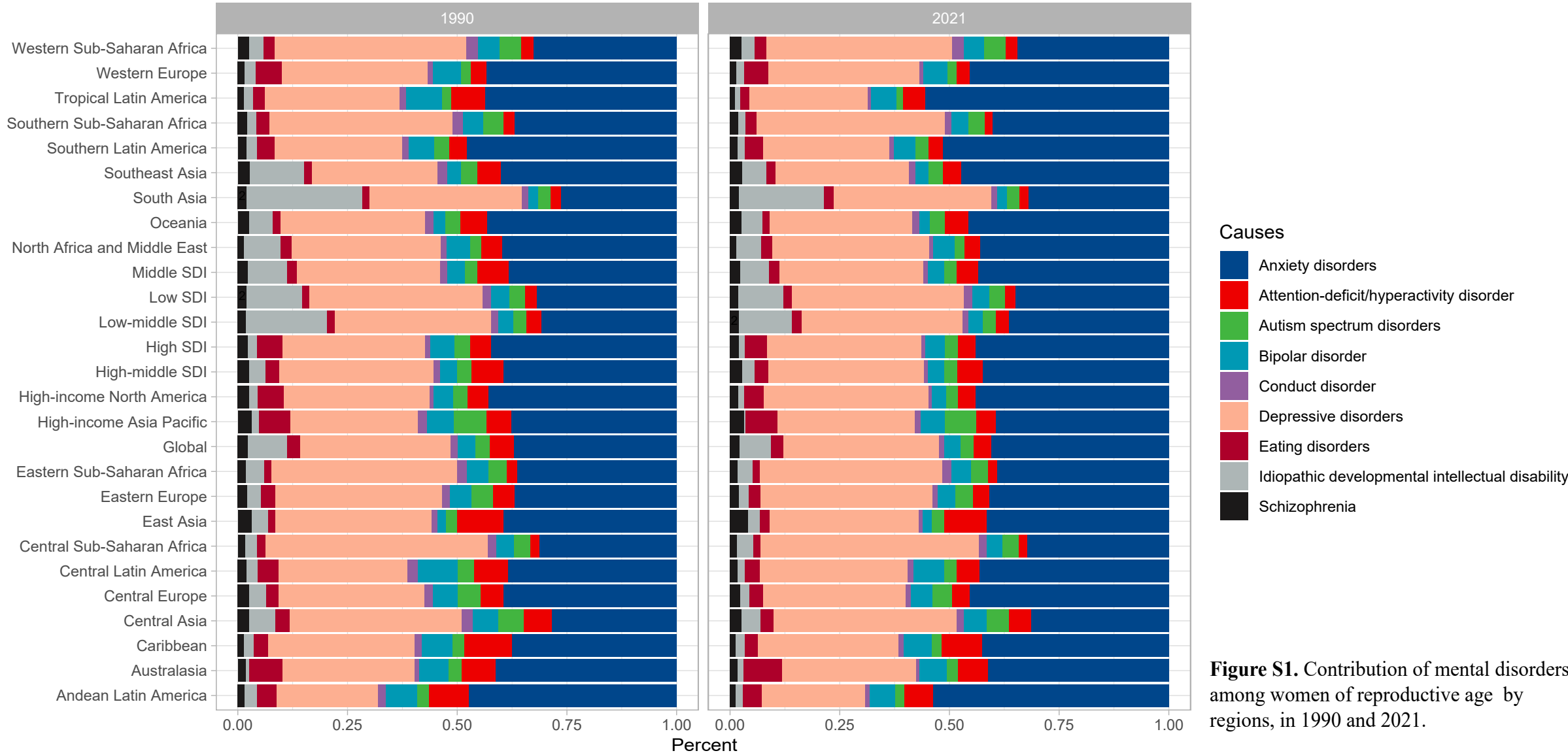

Supplement: Supplementary file 1 — Supporting Information Table S1 and Table S3 provide the information on the disease burden of mental disorders in WCBA at the national level. Table S2 and Table S4 provide additional details on changes in the burden of the top two mental disorders in WCBA in 204 countries. Table S5 and Table S6 provide the information on the global age structure of the top two mental disorders burden among WCBA in 2021. Table S7 and Table S10 provide additional details on temporal joinpoint analysis of the top two mental disorders ASPR from 1990 to 2021. Table S8 and Table S11 provide additional details on temporal joinpoint analysis of the top two mental disorders ASIR from 1990 to 2021. Table S9 and Table S12 provide additional details on temporal joinpoint analysis of the top two mental disorders ASDR from 1990 to 2021. Table S13 and Table S14 provide additional details on the health‐related variables in WCBA across the top 20 and bottom 20 countries and territories in 2021, sorted by 2021 ASIR of the top two mental disorders. Table S15 provides additional details on correlation between ASIR of depression and anxiety disorders and health‐related variables among WCBA in 2021. Figures S1 and S2 provide additional details on contributions and change in burden of mental disorders. [file DA-2026-8872094-s001.zip › Figure S1.pdf]
